# Hepatitis E Seroprevalence and Detection of Genotype 3 Strains in Domestic Pigs from Sierra Leone Collected in 2016 and 2017

**DOI:** 10.3390/v16040558

**Published:** 2024-04-03

**Authors:** Roland Suluku, Juliet Jabaty, Kerstin Fischer, Sandra Diederich, Martin H. Groschup, Martin Eiden

**Affiliations:** 1 Animal Science, Serology and Molecular Laboratory, Njala University, Bo, Sierra Leone; nyasulukuroland2710@gmail.com; 2 Sierra Leone Agricultural Research Institute, Teko Livestock Research Centre, Teko, Sierra Leone; julietjabaty2012@yahoo.com; 3 Institute of Novel and Emerging Infectious Diseases (INNT), Friedrich-Loeffler-Institut, 17493 Greifswald-Insel Riems, Germany; kerstin.fischer@fli.de (K.F.); sandra.diederich@fli.de (S.D.); martin.groschup@fli.de (M.H.G.); 4 Partner Site Hamburg-Lübeck-Borstel-Riems, German Centre for Infection Research (DZIF), 17493 Greifswald-Insel Riems, Germany

**Keywords:** hepatitis E virus, Sierra Leone, pigs, genotype 3

## Abstract

Hepatitis E virus (HEV) is the main cause of acute hepatitis in humans worldwide and is responsible for a large number of outbreaks especially in Africa. Human infections are mainly caused by genotypes 1 and 2 of the genus *Paslahepevirus*, which are exclusively associated with humans. In contrast, viruses of genotypes 3 and 4 are zoonotic and have their main reservoir in domestic and wild pigs, from which they can be transmitted to humans primarily through the consumption of meat products. Both genotypes 3 and 4 are widespread in Europe, Asia, and North America and lead to sporadic cases of hepatitis E. However, there is little information available on the prevalence of these genotypes and possible transmission routes from animal reservoirs to humans in African countries. We therefore analysed 1086 pig sera collected in 2016/2017 in four districts in Sierra Leone for antibodies against HEV using a newly designed in-house ELISA. In addition, the samples were also analysed for HEV RNA by quantitative real-time RT-PCR. The overall seroprevalence in Sierra Leone was low with only 44 positive sera and a prevalence of 4.0%. Two serum pools were RT-PCR-positive and recovered partial sequences clustered into the genotype 3 (HEV-3) of the order *Paslahepevirus*, species *Paslahepevirus balayani*. The results are the first evidence of HEV-3 infection in pigs from Sierra Leone and demonstrate a low circulation of the virus in these animals to date. Further studies should include an examination of humans, especially those with close contact with pigs and porcine products, as well as environmental sampling to evaluate public health effects within the framework of a One Health approach.

## 1. Introduction

Hepatitis E virus (HEV), the only member of the *Hepeviridae* family, is a small, non-enveloped virus with a single-stranded RNA genome of about 7200 bases in length [1]. Infections with HEV can lead to acute liver inflammation in humans that are in most cases moderate and self-limiting with a mortality rate of 0.5–4% [2]. Severe courses of disease leading to fatalities occur mainly in high-risk groups. Pregnant women in particular show severe disease progression of HEV genotype 1 (HEV-1) infections, while severe courses of HEV-3 infections are common in persons with a pre-damaged liver. This genotype also triggers chronic HEV infections in immunocompromised individuals, especially transplant patients, often leading to fatal cirrhosis. In addition to manifestation in the liver, extrahepatic courses of disease have also been described, characterized primarily by neurologic symptoms as found in Guillain–Barré Syndrome, by meningoencephalitis or neuritis [3].

According to the recently updated nomenclature, the *Hepeviridae* family is divided into two subfamilies *Orthohepevirinae* and *Parahepevirinae* with the former again divided into four genera [4]. Genus *Paslahepevirus* species *Paslahepevirus balayani* covers genotypes 1 and 2 (HEV-1/2), which are restricted to humans and are associated with large waterborne epidemics in countries with poor sanitation. This includes HEV-1 in Asia and Africa and HEV-2 in Central America and Central Africa [5]. Throughout Africa, numerous large outbreaks have been reported with more than 10,000 cases in Somalia in 1988, more than 1000 cases in Kenya in 1991 and Sudan in 2004, more than 10,000 cases in Uganda in 2009, and more than 1000 cases in South Sudan in 2013 [6]. This also encompasses outbreaks among refugees and displaced populations in sub-Saharan Africa with more than 30,000 cases of acute HEV and more than 610 deaths during 2010–2020 [7]. The infections were caused in all these cases by the HEV-1 and HEV-2 genotypes. Only a few reports were published that dealt with the zoonotic genotype HEV-3, which also belongs to the *Paslahepevirus balayani* species. This genotype infects both pigs and humans, resulting in sporadic human cases of hepatitis E. In contrast to humans, HEV infection is asymptomatic in pigs [8]. Corresponding case reports came from Egypt in 1992 [9], Mayotte in 2009 [10], Madagascar in 2008/2009 [11], and an HIV-infected patient in Uganda [12]. In addition, HEV-3 isolates could also be detected in wastewater in Tunisia [13].

Up to now, only a few epidemiological studies on HEV infections in pigs have been carried out in African countries. The first findings were reported from Democratic Republic of the Congo [14], Madagascar [11], Nigeria [15], Cameroon [16,17,18], South Africa [19,20,21], Burkina Faso [22], Zambia [23], Ethiopia [24], and Ghana [25]. This is the first study to test pig samples from Sierra Leone for the presence of HEV, providing new insights into the spread of the virus in the country’s pig population.

## 2. Materials and Methods

### 2.1. Sampling

A total of 1086 porcine sera were collected in 4 different districts of Sierra Leone in 2016 and 2017 (Bombali, Moyamba, Port Loko, and Bo District). All available metadata on the pigs’ age, sex, breed, habitat, and housing conditions were recorded and are listed in Supplementary Table S1 (see Supplementary Table S1). Samples were collected according to a Njala University Institutional Review Board protocol (no. IRB00008861/FWA00018924). Sera were heat-inactivated for 30 min at 60 °C before further analysis [26].

### 2.2. Serology

For the in-house ELISA, a bacterial (BL21(DE3) cells)-expressed protein p239-encompassing amino acid sequence 368–606 of the ORF2 capsid protein was used as genotype 3 capsid antigen. The corresponding sequence of a wild boar HEV strain (accession number: KP294371) was cloned into vector pET-19b, which includes coding sequence of an N-terminal His-Tag. Purification of BL21(DE3)-expressed protein was performed according to standard protocols under denaturing conditions in urea using Ni-NTA columns (Qiagen, Germany). The eluted protein was dialysed against 50 mM natrium carbonate buffer pH 9.6 and stored at −20 °C until use. For the ELISA, Maxisorb plates (Nunc, Denmark) were coated with 100 µL recombinant p239 protein diluted to a concentration of 1 µg/mL with 0.05 M natrium carbonate bicarbonate buffer, pH 9.6, and incubated overnight at 4 °C. Afterward, the plates were washed three times with 300 µL washing buffer containing phosphate-buffered saline (PBS), pH 7.2, and 0.1% Tween 20. Then, they were blocked with 200 µL/well 10% skim milk (DIFCO™) in PBS and incubated for 1 h at 37 °C in a moist chamber. Monoclonal antibody (mAb) 6A2, raised against the capsid protein, was used as positive control in a dilution of 1:5000 in PBS. Sampled pig sera (negative control and serum samples) were diluted 1:25 in PBS buffer. A volume of 100 µL of each sample or control sample was added to the plates. After incubation at 37 °C for 1 h in a moist chamber, plates were washed three times with washing buffer. A volume of 100 µL per well of horseradish peroxidase (HRP)-conjugated Protein G (Calbiochem) diluted 1:5000 in PBS buffer was then added, and the plates were incubated again for 1 h. After a final washing step, 100 µL per well of 2,20-azinodiethylbenzothiazoline sulfonic acid (ABTS; Roche, Mannheim, Germany) substrate was added and plates were incubated for 30 min at room temperature in the dark. The reaction was stopped by the addition of 1% sodium dodecyl sulphate (SDS), and the optical density (OD value) was determined at 405 nm. The results were expressed as a relative OD (OD of sample/OD positive control *100).

The ELISA was validated against German pig sera that were pretested with two commercial ELISA (PrioCHECK™ HEV Antibody ELISA; ThermoFisher, Dreiech, Germany) and ID Screen^®^ Hepatitis E Indirect Multi-species ELIISA (ID Vet, Montpellier, France). ROC analysis determined a sensitivity of 96.1% and a specificity of 96.8% with a cut-off of 36.5 (relative OD) for our in-house ELISA (Supplementary Table S2).

### 2.3. RNA Isolation and Molecular Analysis

RNA was extracted by QIAmp Viral Mini Kit (Qiagen GmbH, Hilden, Germany) according to the manufacturer’s manual, subjected in quintuplicate to an HEV-specific quantitative real-time RT-PCR [27], including an adapted β-actin-specific qRT-PCR as internal PCR control [28]. Pooling five samples for viral RNA detection by real-time RT-PCR is a proven strategy to save resources and reduce costs without compromising test sensitivity. The used primers were ACT-1030-F (5′-AGC GCA AGT ACT CCG TGT G-3′), ACT-1135-R (5′-CGG ACT CAT CGT ACT CCT GCT T-3′), and ACT-1081-HEX (HEX- TCG CTG TCC ACC TTC CAG CAG ATG T -BHQ1). For phylogenetic analysis, partial sequences were amplified either targeting the ORF1 region or the RNA-dependent RNA polymerase (RdRp) region using a nested RT-PCR protocol (i) ORF1: primer HEV.ORF1_F1 (5′-CCCAYCAGTTYATWAAGGCTCCTGGC-3′) and HEV.ORF1_R1 (5′-TGCARDGARTANARRGCNAYNCCNGTCTC-3′) followed by second-round primers HEV.ORF1_F2 (5′-AAYTCYGCCYTGG CGAATGCTG TGGTGGT-3′) and HEV.ORF1_R2 (5′-CCVCGRGTNG GRGCRGWRTACCA-3′). RdRp primers HEV.RdRp_F1 (5′-TCGCGCATC ACMTTYTTCCARAA-3′) and HEV.RdRp_R1 (5′-GCCATGTTCCAGA CDGTRTT CCA-3′) and second-round primers HEV.RdRp_F2b (5′-GTGCT CTGTTTGGCCCNTGG TTYMG-3′) and HEV.RdRp_R2 (5′-CCAGGCTCA CCR GARTGYTTCTTCCA-3′). Reverse transcription was carried out with Superscript^®^ III Reverse Transcriptase (Thermo Fisher Scientific Inc., Waltham, MA, USA) and the subsequent nested PCR with Maxima SYBR Green/Fluorescein qPCR Master Mix Kit (Thermo Fisher Scientific Inc., USA). Finally, a melting curve analysis was performed starting with a temperature gradient from 68 to 94 °C in steps of 0.2 °C. Positive samples were identified by melting peaks, and amplicons were subsequently sequenced (Eurofins Genomics, Munich, Germany). Detailed PCR protocols are available from a previous publication [27].

### 2.4. Phylogenetic Analysis

A phylogenetic analysis was performed with Geneious Tree Builder using Neighbor-Joining analysis, and genetic distances were determined with the Tamura–Nei method. Bootstrap values >70 are displayed at the nodes. A phylogenetic analysis was carried out with a 160-nucleotide fragment of the RNA-dependent RNA polymerase (RdRp) as well as with a 270-nucleotide fragment of the methyltransferase (MeT) gene. The sequence of avian hepatitis E virus strain KF511797 was used as outgroup to root the tree.

## 3. Results

Individual sera were analysed with the established in-house ELISA based on an HEV-3 capsid antigen. The serological screening revealed a seroprevalence of 4.0% and 4.1% in pig sera collected in 2016 and 2017 from Sierra Leone, respectively. A summary of the results is depicted in Table 1. In total, 1086 pig serum samples from Sierra Leone were analysed in 2016 (n = 396) as well as in 2017 (n = 690). Samples were collected in more than 50 holdings from four different districts and included different breeds (West African Dwarf, Berkshire, Large White, and Duroc) as well as multiple crossbreeds (Supplementary Table S2).

**Figure 1:**
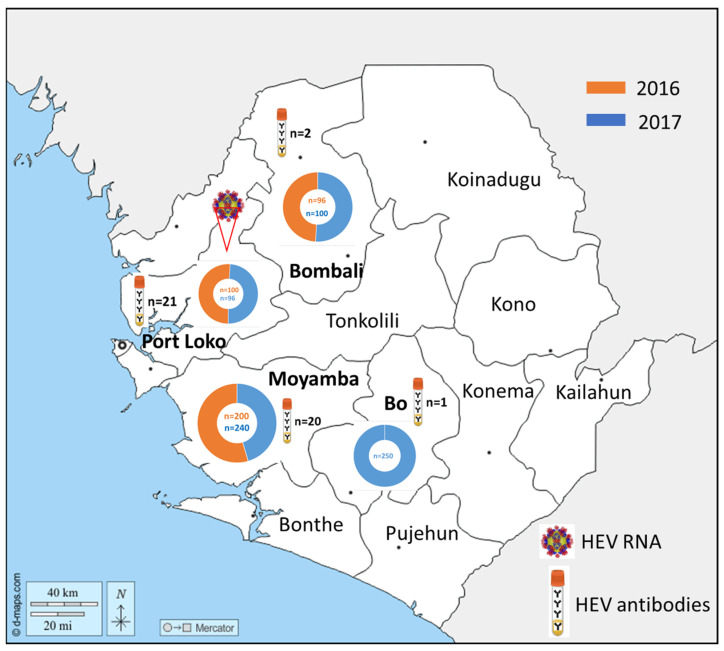
Geographical distribution of pig sera sampled in 2016 (orange coloured) and 2017 (blue coloured) in Sierra Leone. Determined HEV antibody-positive sera and viral RNA are displayed in corresponding symbols (https://d-maps.com/carte.php?num_car=27754&lang=de, accessed on 10 March 2024).

In detail, positive samples for the year 2016 came mostly from Northern/Port Loko (n = 14, prevalence: 14.0%) as well as from South Moyamba (n = 2, 1.0%). In 2017, positive sera were found in the northern region of Bombali (n = 2, 1.9%), Northern/Port Loko (n = 7, 7.3%), Bo (n = 1, 0.4%), Northern/Bombali (n = 2, 1.9%), and South Moyamba (n = 18, 7.5%). The results were summarized and are illustrated on a geographical map of Sierra Leone’s districts (Figure 1).

**Figure 2:**
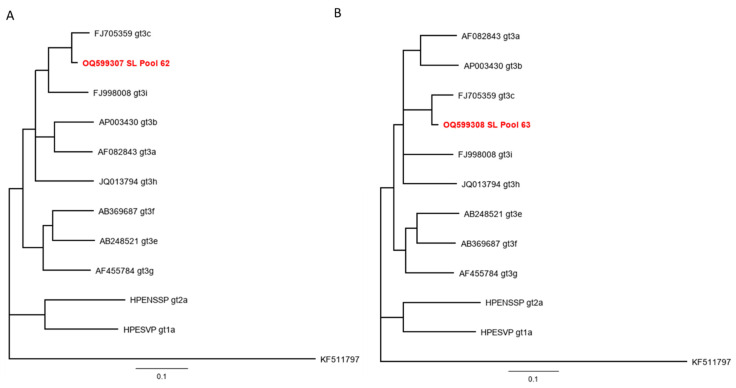
Neighbour-joining phylogenetic tree based on partial RNA-dependent RNA polymerase (**A**) and methyltransferase (**B**) sequences. Red boldface letters indicate pig sequences from serum pool 62 and serum pool 63, respectively. Scale bar indicates mean number of substitutions per site.

Finally, all sera were combined into pools of five and subjected to molecular analysis by an HEV-specific qRT-PCR. Two pools, collected in 2016, from two different farms in Port Loko were found to be positive for HEV (pool 62 and pool 63). From both pools, partial sequences could be recovered either from the RdRp gene (Pool 62) or the methyltransferase (MeT) gene (pool 63). A phylogenetic analysis assigned them both to the HEV-3 sub-genotype 3c (Figure 2). A phylogenetic analysis was carried out with a 160-nucleotide fragment of the RdRp (Figure 2A) as well as with a 270-nucleotide fragment of the MeT gene (Figure 2B).

## 4. Discussion

Pig farming is an important basis of livelihood in many parts of Africa, especially in rural communities. It also serves as a source of income and thus helps to reduce poverty. By addressing these factors, pig production has the potential to boost the real per capita income of sub-Saharan African countries [29]. However, with the intensification of global animal production, the interfaces for transmission between species and the reservoir population for zoonotic viruses have increased [30]. HEV in particular is an exemplary zoonosis at the human–animal–environment interface that can be addressed through the One Health approach, which recognizes the close link among humans, animals, and ecosystem health [31]. Because the majority of HEV studies conducted in Africa to date have focused on HEV infections in humans with HEV-1 and HEV-2, surveillance should also address HEV genotype 3 with pigs as the main reservoir host, as well as other susceptible animals.

Our investigation provides the first data on HEV infection in pigs in Sierra Leone so far, indicating low seroprevalence rates of about 4.0%. This is in contrast to other African countries that recorded significantly higher prevalences including 43.2% in Cameroon [17]; 47.4% in Zambia [23]; 32.8% [32], 55.6% [15], 57.5% [33], and 65.7% [34] in Nigeria; 77.5% in Ghana [25]; and up to 80.7% in Burkina Faso [22]. The large differences can be attributed to the collection regimen of the samples analysed. In our study, the samples came from a variety of different farms, usually small holdings, which may explain the low overall prevalence. Due to the exceptional high susceptibility of pigs to HEV, with a minimum infectious dose of 24 IU RNA/ml [35], a single entry can infect a few animals or a large herd in a very short time. This could lead to an overestimation of positivity in a study, even if only few or single farms were HEV positive. For instance, a study conducted in Nigeria examined pigs from a sole farm and revealed a seroprevalence rate of approximately 97%. [36]. The study design for such surveys must therefore be defined very precisely in advance.

Only few pig-derived HEV sequences have been reported so far throughout Africa. They exclusively belong to HEV-3 genotypes: In DR Congo, genotype 3c [14]; in Cameroon, an undefined subtype [16]; probably genotype 3e in Nigeria [15]; subtype 3h in Ghana [25]; and subtype 3c in South Africa [21]. In addition, viral RNA was detected in pork products in Cape Town, which clustered to subtype 3e [20]. The same subtype was isolated from a patient in Cape Town, who suffered from acute liver failure [37]. Another study from Burkina Faso showed that anti-HEV prevalence among pork butchers was significantly higher compared to the general population suggesting close and frequent contact with infected pork products [38]. A similar observation was reported in Accra, Ghana, regarding pig handlers with an increased anti-HEV prevalence and elevated serum ALT and AST levels [39]. These findings generally demonstrate the potential risk posed by the pig-to-human transmission of HEV by strains circulating in pigs and humans. As there are only a few studies on HEV-3 so far, it has not yet been possible to obtain a comprehensive overview of the prevalence of this genotype in the African population. This may also be due to the fact that most of the infections with HEV-3 are clinically inapparent in healthy people.

## 5. Conclusions

In summary, our study underlines the importance of continuous surveillance and monitoring of HEV in both animal and human populations to prevent and control potential outbreaks according to the One Health concept. Overall, these findings serve as a foundation for future investigations and inform targeted interventions to mitigate the risks associated with HEV transmission in Sierra Leone and beyond.

## Figures and Tables

**Table 1:** Results of the serological investigations of pig sera collected in Sierra Leone.

Country	Year	Total No. of Animals	Results of ELISA			Prevalence
			# Positive	# Incl.	# Negative	
Sierra Leone	2016	396	16	2	378	4.0%
	2017	690	28	2	671	4.1%
**Total**		**1086**	**44**	**4**	**1049**	**4.0%**

Legend. Incl.: inconclusive.

## Data Availability

The sequences of the ORFV whole genomes obtained during the present study are openly available in the GenBank nucleotide sequence database under the accession numbers OQ599307-OQ599308.

## Supplementary Materials

The following supporting information can be downloaded at: https://www.mdpi.com/article/10.3390/v16040558/s1, Table S1: Sampling data; Table S2: Validation data Hepatitis E Virus ELISA.
