# Supplementary material for: Hepatitis E Seroprevalence and Detection of Genotype 3 Strains in Domestic Pigs from Sierra Leone Collected in 2016 and 2017
Source: Viruses. 2024 Apr 3;16(4):558. doi: 10.3390/v16040558 (PMC11054517; doi:10.3390/v16040558)

[illegible]	Designation FLI	Serum-Pool Nr	Findings (PCR)	OD405	Finding	Region	District	Chiefdom	Sampling Unit/Town/Village	Herd/Flock/Farm	Animal Species	Breed	Date of sample collection	Habitat	Housing/stable/Pasture /Stationary	History of migration	Gender/M/F	Age in month
[illegible]P/MOY/[illegible]01	SLE/ 1/16 SW			0,11	neg	South	Moyamba	Kori	University Farm, Njala Campus	Flock	Swine	Large White	13.12.2016	Grassland	Stationary	Bo/Guinea/Moyamba	F	7
[illegible]02	SLE/ 2/16 SW			0,15	neg	South	Moyamba	Kori	University Farm, Njala Campus	Flock	Swine	Large White	13.12.2016	Grassland	Stationary	Bo/Guinea/Moyamba	F	7
SLP/MOY/003	SLE/ 3/16 SW	Pool 1	neg	0,11	neg	South	Moyamba	Kori	University Farm, Njala Campus	Flock	Swine	Large White	13.12.2016	Grassland	Stationary	Bo/Guinea/Moyamba	F	7
SLP/MOY/004	SLE/ 4/16 SW			0,17	neg	South	Moyamba	Kori	University Farm, Njala Campus	Flock	Swine	Large White	13.12.2016	Grassland	Stationary	Bo/Guinea/Moyamba	M	12
SLP/MOY/005	SLE/ 5/16 SW			0,15	neg	South	Moyamba	Kori	University Farm, Njala Campus	Flock	Swine	Large White	13.12.2016	Grassland	Stationary	Bo/Guinea/Moyamba	M	12
SLP/MOY/006	SLE/ 6/16 SW			0,18	neg	South	Moyamba	Kori	University Farm, Njala Campus	Flock	Swine	Large White	13.12.2016	Grassland	Stationary	Bo/Guinea/Moyamba	F	12
SLP/MOY/007	SLE/ 7/16 SW			0,11	neg	South	Moyamba	Kori	University Farm, Njala Campus	Flock	Swine	Large White	13.12.2016	Grassland	Stationary	Bo/Guinea/Moyamba	F	12
SLP/MOY/008	SLE/ 8/16 SW	Pool 2	neg	0,13	neg	South	Moyamba	Kori	University Farm, Njala Campus	Flock	Swine	Large White	13.12.2016	Grassland	Stationary	Bo/Guinea/Moyamba	F	6
SLP/MOY/009	SLE/ 9/16 SW			0,18	neg	South	Moyamba	Kori	University Farm, Njala Campus	Flock	Swine	Large White	13.12.2016	Grassland	Stationary	Bo/Guinea/Moyamba	F	6
SLP/MOY/010	SLE/ 10/16 SW			0,13	neg	South	Moyamba	Kori	University Farm, Njala Campus	Flock	Swine	Large White	13.12.2016	Grassland	Stationary	Bo/Guinea/Moyamba	F	6
SLP/MOY/011	SLE/ 11/16 SW			0,12	neg	South	Moyamba	Kori	University Farm, Njala Campus	Flock	Swine	Large White	13.12.2016	Grassland	Stationary	Bo/Guinea/Moyamba	F	9
SLP/MOY/012	SLE/ 12/16 SW			0,20	neg	South	Moyamba	Kori	University Farm, Njala Campus	Flock	Swine	Large White	13.12.2016	Grassland	Stationary	Bo/Guinea/Moyamba	M	9
SLP/MOY/013	SLE/ 13/16 SW	Pool 3	neg	0,11	neg	South	Moyamba	Kori	University Farm, Njala Campus	Flock	Swine	Large White	13.12.2016	Grassland	Stationary	Bo/Guinea/Moyamba	M	9
SLP/MOY/014	SLE/ 14/16 SW			0,15	neg	South	Moyamba	Dasse	Domboma	Flock	Swine	West Africa Dwarf	13.12.2016	Farm Bush	Stationary	Mano	M	24
SLP/MOY/015	SLE/ 15/16 SW			0,20	neg	South	Moyamba	Dasse	Domboma	Flock	Swine	West Africa Dwarf	13.12.2016	Farm Bush	Stationary	Mano	M	7
SLP/MOY/016	SLE/ 16/16 SW			0,17	neg	South	Moyamba	Dasse	Domboma	Flock	Swine	West Africa Dwarf	13.12.2016	Farm Bush	Stationary	Mano	F	18
SLP/MOY/017	SLE/ 17/16 SW			0,17	neg	South	Moyamba	Dasse	Domboma	Flock	Swine	West Africa Dwarf	13.12.2016	Farm Bush	Stationary	Mano	F	12
SLP/MOY/018	SLE/ 18/16 SW	Pool 4	neg	0,16	neg	South	Moyamba	Dasse	Domboma	Flock	Swine	West Africa Dwarf	13.12.2016	Farm Bush	Stationary	Mano	F	36
SLP/MOY/019	SLE/ 19/16 SW			0,11	neg	South	Moyamba	Dasse	Domboma	Flock	Swine	West Africa Dwarf	13.12.2016	Farm Bush	Stationary	Mano	F	12
SLP/MOY/020	SLE/ 20/16 SW			0,15	neg	South	Moyamba	Dasse	Domboma	Flock	Swine	West Africa Dwarf	13.12.2016	Farm Bush	Stationary	Mano	F	9
SLP/MOY/021	SLE/ 21/16 SW			0,12	neg	South	Moyamba	Kowa	Kaiyehun	Farm	Swine	West Africa Dwarf	15.12.2016	Farm Bush	Stationary	Njama	F	24
SLP/MOY/022	SLE/ 22/16 SW			0,22	neg	South	Moyamba	Kowa	Kaiyehun	Farm	Swine	West Africa Dwarf	15.12.2016	Farm Bush	Stationary	Njama	F	24
SLP/MOY/023	SLE/ 23/16 SW	Pool 5	neg	0,20	neg	South	Moyamba	Kowa	Kaiyehun	Farm	Swine	West Africa Dwarf	15.12.2016	Farm Bush	Stationary	Njama	M	12
SLP/MOY/024	SLE/ 24/16 SW			0,17	neg	South	Moyamba	Kowa	Kaiyehun	Farm	Swine	West Africa Dwarf	15.12.2016	Farm Bush	Stationary	Njama	F	18
SLP/MOY/025	SLE/ 25/16 SW			0,17	neg	South	Moyamba	Kowa	Kaiyehun	Farm	Swine	West Africa Dwarf	15.12.2016	Farm Bush	Stationary	Njama	M	18
SLP/MOY/026	SLE/ 26/16 SW			0,16	neg	South	Moyamba	Kowa	Kaiyehun	Farm	Swine	West Africa Dwarf	15.12.2016	Farm Bush	Stationary	Njama	F	19
SLP/MOY/027	SLE/ 27/16 SW			0,11	neg	South	Moyamba	Kowa	Kaiyehun	Flock	Swine	West Africa Dwarf	15.12.2016	Farm Bush	Stationary	Njama	M	24
SLP/MOY/028	SLE/ 28/16 SW	Pool 6	neg	0,15	neg	South	Moyamba	Kowa	Kaiyehun	Flock	Swine	West Africa Dwarf	15.12.2016	Farm Bush	Stationary	Njama	F	18
SLP/MOY/029	SLE/ 29/16 SW			0,12	neg	South	Moyamba	Kowa	Kaiyehun	Flock	Swine	West Africa Dwarf	15.12.2016	Farm Bush	Stationary	Njama	F	12
SLP/MOY/030	SLE/ 30/16 SW			0,22	neg	South	Moyamba	Kowa	Kaiyehun	Flock	Swine	West Africa Dwarf	15.12.2016	Farm Bush	Stationary	Njama	F	12
SLP/MOY/031	SLE/ 31/16 SW			0,17	neg	South	Moyamba	Kowa	Kaiyehun	Flock	Swine	West Africa Dwarf	15.12.2016	Farm Bush	Stationary	Njama	F	24
SLP/MOY/032	SLE/ 32/16 SW			0,14	neg	South	Moyamba	Kowa	Kaiyehun	Flock	Swine	West Africa Dwarf	15.12.2016	Farm Bush	Stationary	Njama	M	36
SLP/MOY/033	SLE/ 33/16 SW	Pool 7	neg	0,14	neg	South	Moyamba	Kowa	Kaiyehun	Flock	Swine	West Africa Dwarf	15.12.2016	Farm Bush	Stationary	Njama	M	36
SLP/MOY/034	SLE/ 34/16 SW			0,12	neg	South	Moyamba	Kowa	Shenge	Flock	Swine	West Africa Dwarf	15.12.2016	Farm Bush	Stationary	Njama	M	9
SLP/MOY/035	SLE/ 35/16 SW			0,13	neg	South	Moyamba	Kowa	Shenge	Flock	Swine	West Africa Dwarf	15.12.2016	Farm Bush	Stationary	Njama	F	12
SLP/MOY/036	SLE/ 36/16 SW			0,14	neg	South	Moyamba	Kowa	Shenge	Flock	Swine	West Africa Dwarf	15.12.2016	Farm Bush	Stationary	Njama	F	18
SLP/MOY/037	SLE/ 37/16 SW			0,15	neg	South	Moyamba	Kowa	Shenge	Flock	Swine	West Africa Dwarf	15.12.2016	Farm Bush	Stationary	Njama	F	17
SLP/MOY/038	SLE/ 38/16 SW	Pool 8	neg	0,16	neg	South	Moyamba	Kowa	Shenge	Flock	Swine	West Africa Dwarf	15.12.2016	Farm Bush	Stationary	Njama	M	36
SLP/MOY/039	SLE/ 39/16 SW			0,20	neg	South	Moyamba	Kowa	Shenge	Flock	Swine	West Africa Dwarf	15.12.2016	Farm Bush	Stationary	Njama	F	18
SLP/MOY/040	SLE/ 40/16 SW			0,10	neg	South	Moyamba	Kowa	Shenge	Flock	Swine	West Africa Dwarf	15.12.2016	Farm Bush	Stationary	Njama	M	18
SLP/MOY/041	SLE/ 41/16 SW			0,14	neg	South	Moyamba	Kowa	Shenge	Flock	Swine	West Africa Dwarf	15.12.2016	Farm Bush	Stationary	Njama	M	18
SLP/MOY/042	SLE/ 42/16 SW			0,16	neg	South	Moyamba	Kowa	Shenge	Flock	Swine	West Africa Dwarf	15.12.2016	Farm Bush	Stationary	Njama	F	18
SLP/MOY/043	SLE/ 43/16 SW	Pool 9	neg	0,09	neg	South	Moyamba	Kowa	Bateima	Flock	Swine	West Africa Dwarf	15.12.2016	Farm Bush	Stationary	Bo/Moyamba District	F	14
SLP/MOY/044	SLE/ 44/16 SW			0,11	neg	South	Moyamba	Kowa	Bateima	Flock	Swine	West Africa Dwarf	15.12.2016	Farm Bush	Stationary	Bo/Moyamba District	F	14
SLP/MOY/045	SLE/ 45/16 SW			0,15	neg	South	Moyamba	Kowa	Bateima	Flock	Swine	West Africa Dwarf	15.12.2016	Farm Bush	Stationary	Bo/Moyamba District	M	18
SLP/MOY/046	SLE/ 46/16 SW			0,15	neg	South	Moyamba	Kowa	Bateima	Flock	Swine	West Africa Dwarf	15.12.2016	Farm Bush	Stationary	Bo/Moyamba District	M	18
SLP/MOY/047	SLE/ 47/16 SW			0,13	neg	South	Moyamba	Kowa	Bateima	Flock	Swine	West Africa Dwarf	15.12.2016	Farm Bush	Stationary	Bo/Moyamba District	F	18
SLP/MOY/048	SLE/ 48/16 SW	Pool 10	neg	0,13	neg	South	Moyamba	Kowa	Bateima	Flock	Swine	West Africa Dwarf	15.12.2016	Farm Bush	Stationary	Bo/Moyamba District	F	36
SLP/MOY/049	SLE/ 49/16 SW			0,10	neg	South	Moyamba	Kowa	Bateima	Flock	Swine	West Africa Dwarf	15.12.2016	Farm Bush	Stationary	Bo/Moyamba District	F	24
SLP/MOY/050	SLE/ 50/16 SW			0,09	neg	South	Moyamba	Kowa	Wonde	Flock	Swine	Duroc	15.12.2016	Farm Bush	Stationary	Bateima/Njama	M	36
SLP/MOY/051	SLE/ 51/16 SW			0,08	neg	South	Moyamba	Kowa	Wonde	Flock	Swine	Duroc	15.12.2016	Farm Bush	Stationary	Bateima/Njama	F	18
SLP/MOY/052	SLE/ 52/16 SW			0,09	neg	South	Moyamba	Kowa	Wonde	Flock	Swine	Large White	15.12.2016	Farm Bush	Stationary	Bateima/Njama	F	24
SLP/MOY/053	SLE/ 53/16 SW	Pool 11	neg	0,20	neg	South	Moyamba	Kowa	Wonde	Flock	Swine	Large White	15.12.2016	Farm Bush	Stationary	Bateima/Njama	F	14
SLP/MOY/054	SLE/ 54/16 SW			0,12	neg	South	Moyamba	Kowa	Wonde	Flock	Swine	Large White	15.12.2016	Farm Bush	Stationary	Bateima/Njama	F	24
SLP/MOY/055	SLE/ 55/16 SW			0,16	neg	South	Moyamba	Kowa	Wonde	Flock	Swine	Large White	15.12.2016	Farm Bush	Stationary	Bateima/Njama	F	18
SLP/MOY/056	SLE/ 56/16 SW			0,11	neg	South	Moyamba	Kowa	Wonde	Flock	Swine	West Africa Dwarf	15.12.2016	Farm Bush	Stationary	Bateima/Njama	M	18
SLP/MOY/057	SLE/ 57/16 SW			0,10	neg	South	Moyamba	Kowa	Wonde	Flock	Swine	West Africa Dwarf	15.12.2016	Farm Bush	Stationary	Bateima/Njama	F	24
SLP/MOY/058	SLE/ 58/16 SW	Pool 12	neg	0,12	neg	South	Moyamba	Kowa	Wonde	Flock	Swine	West Africa Dwarf	15.12.2016	Farm Bush	Stationary	Bateima/Njama	F	36
SLP/MOY/059	SLE/ 59/16 SW			0,09	neg	South	Moyamba	Kowa	Wonde	Flock	Swine	West Africa Dwarf	15.12.2016	Farm Bush	Stationary	Bateima/Njama	F	12
SLP/MOY/060	SLE/ 60/16 SW			0,15	neg	South	Moyamba	Kowa	Wonde	Flock	Swine	West Africa Dwarf	15.12.2016	Farm Bush	Stationary	Bateima/Njama	F	12
SLP/MOY/061	SLE/ 61/16 SW			0,11	neg	South	Moyamba	Kowa	Wonde	Flock	Swine	West Africa Dwarf	15.12.2016	Farm Bush	Stationary	Bateima/Njama	F	12
SLP/MOY/062	SLE/ 62/16 SW			0,17	neg	South	Moyamba	Kowa	Wonde	Flock	Swine	West Africa Dwarf	15.12.2016	Farm Bush	Stationary	Bateima/Njama	F	24
SLP/MOY/063	SLE/ 63/16 SW	Pool 13	neg	0,14	neg	South	Moyamba	Kowa	Wonde	Flock	Swine	West Africa Dwarf	15.12.2016	Farm Bush	Stationary	Bateima/Njama	F	24
SLP/MOY/064	SLE/ 64/16 SW			0,14	neg	South	Moyamba	Kowa	Wonde	Flock	Swine	West Africa Dwarf	15.12.2016	Farm Bush	Stationary	Bateima/Njama	F	20
SLP/MOY/065	SLE/ 65/16 SW			0,13	neg	South	Moyamba	Kowa	Wonde	Flock	Swine	West Africa Dwarf	15.12.2016	Farm Bush	Stationary	Bateima/Njama	F	24
SLP/MOY/066	SLE/ 66/16 SW			0,14	neg	South	Moyamba	Fakunya	Mboama Johnson	Flock	Swine	Large White	16.12.2016	Farm Bush	Stationary	Fadehun	M	18
SLP/MOY/067	SLE/ 67/16 SW			0,09	neg	South	Moyamba	Fakunya	Mboama Johnson	Flock	Swine	Large White	16.12.2016	Farm Bush	Stationary	Fadehun	M	18
SLP/MOY/068	SLE/ 68/16 SW	Pool 14	neg	0,12	neg	South	Moyamba	Fakunya	Mboama Johnson	Flock	Swine	Large White	16.12.2016	Farm Bush	Stationary	Fadehun	M	12
SLP/MOY/069	SLE/ 69/16 SW			0,20	neg	South	Moyamba	Fakunya	Mboama Johnson	Flock	Swine	Large White	16.12.2016	Farm Bush	Stationary	Fadehun	M	12
SLP/MOY/070	SLE/ 70/16 SW			0,20	neg	South	Moyamba	Fakunya	Mboama Johnson	Flock	Swine	Large White	16.12.2016	Farm Bush	Stationary	Fadehun	M	12
SLP/MOY/071	SLE/ 71/16 SW			0,13	neg	South	Moyamba	Fakunya	Mboama Johnson	Flock	Swine	Large White	16.12.2016	Farm Bush	Stationary	Fadehun	M	24
SLP/MOY/072	SLE/ 72/16 SW			0,15	neg	South	Moyamba	Fakunya	Mboama Johnson	Flock	Swine	West Africa Dwarf	16.12.2016	Farm Bush	Stationary	Fadehun	F	24
SLP/MOY/073	SLE/ 73/16 SW	Pool 15	neg	0,14	neg	South	Moyamba	Fakunya	Mboama Johnson	Flock	Swine	West Africa Dwarf	16.12.2016	Farm Bush	Stationary	Fadehun	F	36
SLP/MOY/074	SLE/ 74/16 SW			0,14	neg	South	Moyamba	Fakunya	Mboama Johnson	Flock	Swine	West Africa Dwarf	16.12.2016	Farm Bush	Stationary	Fadehun	F	36
SLP/MOY/075	SLE/ 75/16 SW			0,15	neg	South	Moyamba	Fakunya	Mboama Johnson	Flock	Swine	West Africa Dwarf	16.12.2016	Farm Bush	Stationary	Fadehun	F	18
SLP/MOY/076	SLE/ 76/16 SW			0,12	neg	South	Moyamba	Fakunya	Mogbetu	Flock	Swine	Large White	16.12.2016	Farm Bush	Stationary	Mboama Johnson	F	9
SLP/MOY/077	SLE/ 77/16 SW			0,10	neg	South	Moyamba	Fakunya	Mogbetu	Flock	Swine	Large White	16.12.2016	Farm Bush	Stationary	Mboama Johnson	M	18
SLP/MOY/078	SLE/ 78/16 SW	Pool 16	Pool 16	0,16	neg	South	Moyamba	Fakunya	Mogbetu	Flock	Swine	Large White	16.12.2016	Farm Bush	Stationary	Mboama Johnson	F	24
SLP/MOY/079	SLE/ 79/16 SW			0,18	neg	South	Moyamba	Fakunya	Mogbetu	Flock	Swine	West Africa Dwarf	16.12.2016	Farm Bush	Stationary	Mboama Johnson	M	12
SLP/MOY/080	SLE/ 80/16 SW			0,19	neg	South	Moyamba	Fakunya	Mogbetu	Flock	Swine	West Africa Dwarf	16.12.2016	Farm Bush	Stationary	Mboama Johnson	F	12
SLP/MOY/081	SLE/ 81/16 SW			0,12	neg	South	Moyamba	Fakunya	Mogbetu	Flock	Swine	West Africa Dwarf	16.12.2016	Farm Bush	Stationary	Mboama Johnson	F	12
SLP/MOY/082	SLE/ 82/16 SW			0,13	neg	South	Moyamba	Fakunya	Mogbetu	Flock	Swine	Large White	16.12.2016	Farm Bush	Stationary	Mboama Johnson	F	12
SLP/MOY/083	SLE/ 83/16 SW	Pool 17	neg	0,15	neg	South	Moyamba	Fakunya	Falaba	Flock	Swine	Large White	16.12.2016	Farm Bush	Stationary	falaba	M	36
SLP/MOY/084	SLE/ 84/16 SW			0,14	neg	South	Moyamba	Fakunya	Falaba	Flock	Swine	Large White	16.12.2016	Farm Bush	Stationary	Gbolima	F	36
SLP/MOY/085	SLE/ 85/16 SW			0,19	neg	South	Moyamba	Fakunya	Falaba	Flock	Swine	Large White	16.12.2016	Farm Bush	Stationary	Gbolima	F	24
SLP/MOY/086	SLE/ 86/16 SW			0,41	neg	South	Moyamba	Fakunya	Falaba	Flock	Swine	West Africa Dwarf	16.12.2016	Farm Bush	Stationary	Gbolima	F	24
SLP/MOY/087	SLE/ 87/16 SW			0,14	neg	South	Moyamba	Fakunya	Falaba	Flock	Swine	West Africa Dwarf	16.12.2016	Farm Bush	Stationary	Gbolima	F	24
SLP/MOY/088	SLE/ 88/16 SW	Pool 18	neg	0,24	neg	South	Moyamba	Fakunya	Falaba	Flock	Swine	West Africa Dwarf	16.12.2016	Farm Bush	Stationary	Gbolima	F	18
SLP/MOY/089	SLE/ 89/16 SW			0,11	neg	South	Moyamba	Fakunya	Falaba	Flock	Swine	West Africa Dwarf	16.12.2016	Farm Bush	Stationary	Gbolima	F	18
SLP/MOY/090	SLE/ 90/16 SW			0,41	neg	South	Moyamba	Fakunya	Falaba	Flock	Swine	West Africa Dwarf	16.12.2016	Farm Bush	Stationary	Gbolima	F	18
SLP/MOY/091	SLE/ 91/16 SW			0,15	neg	South	Moyamba	Fakunya	Falaba	Flock	Swine	West Africa Dwarf	16.12.2016	Farm Bush	Stationary	Gbolima	F	18
SLP/MOY/092	SLE/ 92/16 SW			0,21	neg	South	Moyamba	Fakunya	Falaba	Flock	Swine	Large White	16.12.2016	Farm Bush	Stationary	Gondama	F	24
SLP/MOY/093	SLE/ 93/16 SW	Pool 19	neg	0,24	neg	South	Moyamba	Fakunya	Falaba	Flock	Swine	Large White	16.12.2016	Farm Bush	Stationary	Gondama	F	24
SLP/MOY/094	SLE/ 94/16 SW			0,31	neg	South	Moyamba	Fakunya	Falaba	Flock	Swine	Large White	16.12.2016	Farm Bush	Stationary	same	F	24

SLP/MOY/095	SLE/ 95/16 SW			0,16	neg	South	Moyamba	Fakunya	Falaba	Flock	Swine	Large White	16.12.2016	Farm Bush	Stationary	same	F	24
SLP/MOY/096	SLE/ 96/16 SW			0,19	neg	South	Moyamba	Fakunya	Falaba	Flock	Swine	West Africa Dwarf	16.12.2016	Farm Bush	Stationary	same	F	18
SLP/MOY/097	SLE/ 97/16 SW			0,35	neg	South	Moyamba	Fakunya	Falaba	Flock	Swine	West Africa Dwarf	16.12.2016	Farm Bush	Stationary	Kpamgbamam	F	18
SLP/MOY/098	SLE/ 98/16 SW	Pool 20	neg	0,17	neg	South	Moyamba	Fakunya	Falaba	Flock	Swine	Large White	16.12.2016	Farm Bush	Stationary	Kpamgbamam	F	24
SLP/MOY/099	SLE/ 99/16 SW			0,12	neg	South	Moyamba	Fakunya	Falaba	Flock	Swine	Large White	16.12.2016	Farm Bush	Stationary	Kpamgbama	F	36
SLP/MOY/100	SLE/ 100/16 SW			0,14	neg	South	Moyamba	Kayamba	Korgbotima	Flock	Swine	Large White	17.12.2016	Farm Bush	Stationary	Mokassi	F	12
SLP/MOY/101	SLE/ 101/16 SW			0,19	neg	South	Moyamba	Kayamba	Korgbotima	Flock	Swine	Large White	17.12.2016	Farm Bush	Stationary	Mokassi	F	12
SLP/MOY/102	SLE/ 102/16 SW			0,21	neg	South	Moyamba	Kayamba	Korgbotima	Flock	Swine	Large White	17.12.2016	Farm Bush	Stationary	Mokassi	F	14
SLP/MOY/103	SLE/ 103/16 SW	Pool 21	neg	0,33	neg	South	Moyamba	Kayamba	Korgbotima	Flock	Swine	Large White	17.12.2016	Farm Bush	Stationary	Mokassi	F	18
SLP/MOY/104	SLE/ 104/16 SW			0,12	neg	South	Moyamba	Kayamba	Korgbotima	Flock	Swine	Large White	17.12.2016	Farm Bush	Stationary	Mokassi	F	18
SLP/MOY/105	SLE/ 105/16 SW			0,13	neg	South	Moyamba	Kayamba	Korgbotima	Flock	Swine	Large White	17.12.2016	Farm Bush	Stationary	Mokassi	F	18
SLP/MOY/106	SLE/ 106/16 SW			0,13	neg	South	Moyamba	Kayamba	Korgbotima	Flock	Swine	Large White	17.12.2016	Farm Bush	Stationary	Mokassi	F	24
SLP/MOY/107	SLE/ 107/16 SW			0,12	neg	South	Moyamba	Kayamba	Korgbotima	Flock	Swine	Large White	17.12.2016	Farm Bush	Stationary	Mokassi	F	24
SLP/MOY/108	SLE/ 108/16 SW	Pool 22	neg	0,15	neg	South	Moyamba	Kayamba	Korgbotima	Flock	Swine	Large White	17.12.2016	Farm Bush	Stationary	Mokassi	F	36
SLP/MOY/109	SLE/ 109/16 SW			0,12	neg	South	Moyamba	Kayamba	Korgbotima	Flock	Swine	Large White	17.12.2016	Farm Bush	Stationary	Mokassi	F	18
SLP/MOY/110	SLE/ 110/16 SW			0,18	neg	South	Moyamba	Kayamba	Gondama Kpangbama	Flock	Swine	West Africa Dwarf	18.12.2016	Farm Bush	Stationary	permanent	F	18
SLP/MOY/111	SLE/ 111/16 SW			0,09	neg	South	Moyamba	Kayamba	Gondama Kpangbama	Flock	Swine	West Africa Dwarf	18.12.2016	Farm Bush	Stationary	permanent	F	16
SLP/MOY/112	SLE/ 112/16 SW			0,46	inkl	South	Moyamba	Kayamba	Gondama Kpangbama	Flock	Swine	West Africa Dwarf	18.12.2016	Farm Bush	Stationary	permanent	M	24
SLP/MOY/113	SLE/ 113/16 SW	Pool 23	neg	0,11	neg	South	Moyamba	Kayamba	Gondama Kpangbama	Flock	Swine	West Africa Dwarf	18.12.2016	Farm Bush	Stationary	permanent	F	36
SLP/MOY/114	SLE/ 114/16 SW			0,12	neg	South	Moyamba	Kayamba	Gondama Kpangbama	Flock	Swine	West Africa Dwarf	18.12.2016	Farm Bush	Stationary	permanent	F	36
SLP/MOY/115	SLE/ 115/16 SW			0,08	neg	South	Moyamba	Kayamba	Gondama Kpangbama	Flock	Swine	West Africa Dwarf	18.12.2016	Farm Bush	Stationary	permanent	F	18
SLP/MOY/116	SLE/ 116/16 SW			0,11	neg	South	Moyamba	Kayamba	Gondama Kpangbama	Flock	Swine	West Africa Dwarf	18.12.2016	Farm Bush	Stationary	permanent	F	18
SLP/MOY/117	SLE/ 117/16 SW			0,09	neg	South	Moyamba	Kayamba	Gondama Kpangbama	Flock	Swine	West Africa Dwarf	18.12.2016	Farm Bush	Stationary	permanent	F	18
SLP/MOY/118	SLE/ 118/16 SW	Pool 24	neg	0,14	neg	South	Moyamba	Kayamba	Gondama Kpangbama	Flock	Swine	West Africa Dwarf	18.12.2016	Farm Bush	Stationary	permanent	F	24
SLP/MOY/119	SLE/ 119/16 SW			0,15	neg	South	Moyamba	Kayamba	Gondama Kpangbama	Flock	Swine	West Africa Dwarf	18.12.2016	Farm Bush	Stationary	permanent	M	24
SLP/MOY/120	SLE/ 120/16 SW			0,14	neg	South	Moyamba	Kayamba	Gondama Kpangbama	Flock	Swine	West Africa Dwarf	18.12.2016	Farm Bush	Stationary	permanent	F	24
SLP/MOY/121	SLE/ 121/16 SW			0,73	pos	South	Moyamba	Kayamba	Gondama Kpangbama	Flock	Swine	West Africa Dwarf	18.12.2016	Farm Bush	Stationary	permanent	F	24
SLP/MOY/122	SLE/ 122/16 SW			0,13	neg	South	Moyamba	Kayamba	Gondama Kpangbama	Flock	Swine	West Africa Dwarf	18.12.2016	Farm Bush	Stationary	permanent	F	18
SLP/MOY/123	SLE/ 123/16 SW	Pool 25	neg	0,11	neg	South	Moyamba	Kayamba	Gondama Kpangbama	Flock	Swine	West Africa Dwarf	18.12.2016	Farm Bush	Stationary	permanent	F	18
SLP/MOY/124	SLE/ 124/16 SW			0,08	neg	South	Moyamba	Kayamba	Gondama Kpangbama	Flock	Swine	West Africa Dwarf	18.12.2016	Farm Bush	Stationary	permanent	F	18
SLP/MOY/125	SLE/ 125/16 SW			0,12	neg	South	Moyamba	Kayamba	Gondama Kpangbama	Flock	Swine	West Africa Dwarf	18.12.2016	Farm Bush	Stationary	Kpamgbamam	F	18
SLP/MOY/126	SLE/ 126/16 SW			0,13	neg	South	Moyamba	Kayamba	Gondama Kpangbama	Flock	Swine	West Africa Dwarf	18.12.2016	Farm Bush	Stationary	Kpamgbamam	F	18
SLP/MOY/127	SLE/ 127/16 SW			0,12	neg	South	Moyamba	Kayamba	Gondama Kpangbama	Flock	Swine	West Africa Dwarf	18.12.2016	Farm Bush	Stationary	Kpamgbamam	F	18
SLP/MOY/128	SLE/ 128/16 SW	Pool 26	neg	0,54	pos	South	Moyamba	Kayamba	Gondama Kpangbama	Flock	Swine	West Africa Dwarf	18.12.2016	Farm Bush	Stationary	Kpamgbamam	F	36
SLP/MOY/129	SLE/ 129/16 SW			0,44	neg	South	Moyamba	Kayamba	Gondama Kpangbama	Flock	Swine	West Africa Dwarf	18.12.2016	Farm Bush	Stationary	Kpamgbamam	F	24
SLP/MOY/130	SLE/ 130/16 SW			0,11	neg	South	Moyamba	kori	Taiama	Flock	Swine	Large White	19.12.2016	Farm Bush	Stationary	permanent	F	24
SLP/MOY/131	SLE/ 131/16 SW			0,11	neg	South	Moyamba	kori	Taiama	Flock	Swine	Large White	19.12.2016	Farm Bush	Stationary	permanent	F	12
SLP/MOY/132	SLE/ 132/16 SW			0,16	neg	South	Moyamba	kori	Taiama	Flock	Swine	Large White	19.12.2016	Farm Bush	Stationary	permanent	F	9
SLP/MOY/133	SLE/ 133/16 SW	Pool 27	neg	0,09	neg	South	Moyamba	kori	Taiama	Flock	Swine	Large White	19.12.2016	Farm Bush	Stationary	permanent	F	10
SLP/MOY/134	SLE/ 134/16 SW			0,10	neg	South	Moyamba	kori	Taiama	Flock	Swine	Large White	19.12.2016	Farm Bush	Stationary	permanent	F	7
SLP/MOY/135	SLE/ 135/16 SW			0,16	neg	South	Moyamba	kori	Taiama	Flock	Swine	Large White	19.12.2016	Farm Bush	Stationary	permanent	F	6
SLP/MOY/136	SLE/ 136/16 SW			0,17	neg	South	Moyamba	kori	Taiama	Flock	Swine	Large White	19.12.2016	Farm Bush	Stationary	permanent	F	9
SLP/MOY/137	SLE/ 137/16 SW			0,20	neg	South	Moyamba	kori	Taiama	Flock	Swine	Large White	19.12.2016	Farm Bush	Stationary	permanent	F	9
SLP/MOY/138	SLE/ 138/16 SW	Pool 28	neg	0,10	neg	South	Moyamba	kori	Taiama	Flock	Swine	Large White	19.12.2016	Farm Bush	Stationary	permanent	F	9
SLP/MOY/139	SLE/ 139/16 SW			0,09	neg	South	Moyamba	kori	Taiama	Flock	Swine	Large White	19.12.2016	Farm Bush	Stationary	permanent	F	9
SLP/MOY/140	SLE/ 140/16 SW			0,20	neg	South	Moyamba	kori	Taiama	Flock	Swine	Large White	19.12.2016	Farm Bush	Stationary	permanent	F	7
SLP/MOY/141	SLE/ 141/16 SW			0,10	neg	South	Moyamba	kori	Taiama	Flock	Swine	Large White	19.12.2016	Farm Bush	Stationary	permanent	M	7
SLP/MOY/142	SLE/ 142/16 SW			0,21	neg	South	Moyamba	kori	Taiama	Flock	Swine	Large White	19.12.2016	Farm Bush	Stationary	permanent	M	7
SLP/MOY/143	SLE/ 143/16 SW	Pool 29	neg	0,14	neg	South	Moyamba	kori	Taiama	Flock	Swine	Large White	19.12.2016	Farm Bush	Stationary	permanent	F	7
SLP/MOY/144	SLE/ 144/16 SW			0,24	neg	South	Moyamba	kori	Taiama	Flock	Swine	Large White	19.12.2016	Farm Bush	Stationary	permanent	F	7
SLP/MOY/145	SLE/ 145/16 SW			0,10	neg	South	Moyamba	kori	Taiama	Flock	Swine	Large White	19.12.2016	Farm Bush	Stationary	permanent	F	7
SLP/MOY/146	SLE/ 146/16 SW			0,10	neg	South	Moyamba	kori	Taiama	Flock	Swine	Large White	19.12.2016	Farm Bush	Stationary	permanent	M	7
SLP/MOY/147	SLE/ 147/16 SW			0,13	neg	South	Moyamba	kori	Taiama	Flock	Swine	Large White	19.12.2016	Farm Bush	Stationary	permanent	F	18
SLP/MOY/148	SLE/ 148/16 SW	Pool 30	neg	0,10	neg	South	Moyamba	kori	Taiama	Flock	Swine	Large White	19.12.2016	Farm Bush	Stationary	permanent	M	9
SLP/MOY/149	SLE/ 149/16 SW			0,12	neg	South	Moyamba	kori	Taiama	Flock	Swine	Large White	19.12.2016	Farm Bush	Stationary	permanent	F	9
SLP/MOY/150	SLE/ 150/16 SW			0,13	neg	South	Moyamba	kori	Taiama	Flock	Swine	Large White	19.12.2016	Farm Bush	Stationary	permanent	M	9
SLP/MOY/151	SLE/ 151/16 SW			0,13	neg	South	Moyamba	kori	Taiama	Flock	Swine	Large White	19.12.2016	Farm Bush	Stationary	permanent	F	7
SLP/MOY/152	SLE/ 152/16 SW			0,11	neg	South	Moyamba	kori	Taiama	Flock	Swine	Large White	19.12.2016	Farm Bush	Stationary	permanent	F	7
SLP/MOY/153	SLE/ 153/16 SW	Pool 31	neg	0,11	neg	South	Moyamba	kori	Taiama	Flock	Swine	Large White	19.12.2016	Farm Bush	Stationary	permanent	F	7
SLP/MOY/154	SLE/ 154/16 SW			0,08	neg	South	Moyamba	kori	Taiama	Flock	Swine	Large White	19.12.2016	Farm Bush	Stationary	permanent	F	7
SLP/MOY/155	SLE/ 155/16 SW			0,09	neg	South	Moyamba	kori	Taiama	Flock	Swine	Large White	19.12.2016	Farm Bush	Stationary	permanent	M	7
SLP/MOY/156	SLE/ 156/16 SW			0,10	neg	South	Moyamba	kori	Taiama	Flock	Swine	Large White	19.12.2016	Farm Bush	Stationary	permanent	M	9
SLP/MOY/157	SLE/ 157/16 SW			0,11	neg	South	Moyamba	kori	Taiama	Flock	Swine	Large White	19.12.2016	Farm Bush	Stationary	permanent	M	9
SLP/MOY/158	SLE/ 158/16 SW	Pool 32	neg	0,10	neg	South	Moyamba	kori	Taiama	Flock	Swine	Large White	19.12.2016	Farm Bush	Stationary	permanent	M	9
SLP/MOY/159	SLE/ 159/16 SW			0,08	neg	South	Moyamba	kori	Taiama	Flock	Swine	Large White	19.12.2016	Farm Bush	Stationary	permanent	M	9
SLP/MOY/160	SLE/ 160/16 SW			0,13	neg	South	Moyamba	kori	Taiama	Flock	Swine	Large White	19.12.2016	Farm Bush	Stationary	permanent	M	9
SLP/MOY/161	SLE/ 161/16 SW			0,09	neg	South	Moyamba	kori	Taiama	Flock	Swine	Large White	19.12.2016	Farm Bush	Stationary	permanent	F	18
SLP/MOY/162	SLE/ 162/16 SW			0,09	neg	South	Moyamba	kori	Taiama	Flock	Swine	Large White	19.12.2016	Farm Bush	Stationary	permanent	F	18
SLP/MOY/163	SLE/ 163/16 SW	Pool 33	neg	0,09	neg	South	Moyamba	kori	Taiama	Flock	Swine	Large White	19.12.2016	Farm Bush	Stationary	permanent	F	18
SLP/MOY/164	SLE/ 164/16 SW			0,09	neg	South	Moyamba	kori	Taiama	Flock	Swine	Large White	19.12.2016	Farm Bush	Stationary	permanent	F	36
SLP/MOY/165	SLE/ 165/16 SW			0,09	neg	South	Moyamba	kori	Taiama	Flock	Swine	Large White	19.12.2016	Farm Bush	Stationary	permanent	F	36
SLP/MOY/166	SLE/ 166/16 SW			0,13	neg	South	Moyamba	kori	Taiama	Flock	Swine	Large White	19.12.2016	Farm Bush	Stationary	permanent	F	6
SLP/MOY/167	SLE/ 167/16 SW			0,13	neg	South	Moyamba	kori	Taiama	Flock	Swine	Large White	19.12.2016	Farm Bush	Stationary	permanent	F	6
SLP/MOY/168	SLE/ 168/16 SW	Pool 34	neg	0,13	neg	South	Moyamba	kori	Taiama	Flock	Swine	Duroc	19.12.2016	Farm Bush	Stationary	permanent	F	6
SLP/MOY/169	SLE/ 169/16 SW			0,13	neg	South	Moyamba	kori	Taiama	Flock	Swine	Duroc	19.12.2016	Farm Bush	Stationary	permanent	F	36
SLP/MOY/170	SLE/ 170/16 SW			0,09	neg	South	Moyamba	kori	Taiama	Flock	Swine	Large White	19.12.2016	Farm Bush	Stationary	permanent	F	36
SLP/MOY/171	SLE/ 171/16 SW			0,09	neg	South	Moyamba	kori	Taiama	Flock	Swine	Large White	20.12.2016	Farm Bush	Stationary	permanent	M	36
SLP/MOY/172	SLE/ 172/16 SW			0,09	neg	South	Moyamba	kori	Taiama	Flock	Swine	Large White	20.12.2016	Farm Bush	Stationary	permanent	M	8
SLP/MOY/173	SLE/ 173/16 SW	Pool 35	neg	0,10	neg	South	Moyamba	kori	Taiama	Flock	Swine	Large White	20.12.2016	Farm Bush	Stationary	permanent	M	8
SLP/MOY/174	SLE/ 174/16 SW			0,17	neg	South	Moyamba	kori	Taiama	Flock	Swine	Large White	20.12.2016	Farm Bush	Stationary	permanent	F	8
SLP/MOY/175	SLE/ 175/16 SW			0,09	neg	South	Moyamba	kori	Taiama	Flock	Swine	West Africa Dwarf	20.12.2016	Farm Bush	Stationary	permanent	F	8
SLP/MOY/176	SLE/ 176/16 SW			0,11	neg	South	Moyamba	kori	Taiama	Flock	Swine	West Africa Dwarf	20.12.2016	Farm Bush	Stationary	permanent	M	8
SLP/MOY/177	SLE/ 177/16 SW			0,10	neg	South	Moyamba	kori	Taiama	Flock	Swine	West Africa Dwarf	20.12.2016	Farm Bush	Stationary	permanent	M	9
SLP/MOY/178	SLE/ 178/16 SW	Pool 36	neg	0,10	neg	South	Moyamba	kori	Taiama	Flock	Swine	West Africa Dwarf	20.12.2016	Farm Bush	Stationary	permanent	F	11
SLP/MOY/179	SLE/ 179/16 SW			0,09	neg	South	Moyamba	kori	Taiama	Flock	Swine	West Africa Dwarf	20.12.2016	Farm Bush	Stationary	permanent	F	11
SLP/MOY/180	SLE/ 180/16 SW			0,10	neg	South	Moyamba	kori	Taiama	Flock	Swine	West Africa Dwarf	20.12.2016	Farm Bush	Stationary	permanent	F	11
SLP/MOY/181	SLE/ 181/16 SW			0,11	neg	South	Moyamba	kori	Taiama	Flock	Swine	West Africa Dwarf	20.12.2016	Farm Bush	Stationary	permanent	F	12
SLP/MOY/182	SLE/ 182/16 SW			0,17	neg	South	Moyamba	kori	Taiama	Flock	Swine	West Africa Dwarf	20.12.2016	Farm Bush	Stationary	permanent	M	24
SLP/MOY/183	SLE/ 183/16 SW	Pool 37	neg	0,14	neg	South	Moyamba	kori	Taiama	Flock	Swine	West Africa Dwarf	20.12.2016	Farm Bush	Stationary	permanent	F	24
SLP/MOY/184	SLE/ 184/16 SW			0,09	neg	South	Moyamba	kori	Taiama	Flock	Swine	West Africa Dwarf	20.12.2016	Farm Bush	Stationary	permanent	F	10
SLP/MOY/185	SLE/ 185/16 SW			0,09	neg	South	Moyamba	kori	Taiama	Flock	Swine	West Africa Dwarf	20.12.2016	Farm Bush	Stationary	permanent	M	10
SLP/MOY/186	SLE/ 186/16 SW			0,09	neg	South	Moyamba	kori	Taiama	Flock	Swine	West Africa Dwarf	20.12.2016	Farm Bush	Stationary	permanent	M	18
SLP/MOY/187	SLE/ 187/16 SW			0,11	neg	South	Moyamba	kori	Taiama	Flock	Swine	West Africa Dwarf	20.12.2016	Farm Bush	Stationary	permanent	F	18
SLP/MOY/188	SLE/ 188/16 SW	Pool 38	neg	0,12	neg	South	Moyamba	kori	Taiama	Flock	Swine	West Africa Dwarf	20.12.2016	Farm Bush	Stationary	permanent	F	7
SLP/MOY/189	SLE/ 189/16 SW			0,11	neg	South	Moyamba	kori	Taiama	Flock	Swine	West Africa Dwarf	20.12.2016	Farm Bush	Stationary	permanent	F	7
SLP/MOY/190	SLE/ 190/16 SW			0,15	neg	South	Moyamba	kori	Taiama	Flock	Swine	West Africa Dwarf	20.12.2016	Farm Bush	Stationary	permanent	F	7
SLP/MOY/191	SLE/ 191/16 SW			0,26	neg	South	Moyamba	kori	Taiama	Flock	Swine	West Africa Dwarf	20.12.2016	Farm Bush	Stationary	permanent	F	7

SLP/MOY/192	SLE/ 192/16 SW			0,15	neg	South	Moyamba	kori	Taiama	Flock	Swine	West Africa Dwarf	20.12.2016	Farm Bush	Stationary	permanent	M	7
SLP/MOY/193	SLE/ 193/16 SW	Pool 39	neg	0,15	neg	South	Moyamba	kori	Taiama	Flock	Swine	Duroc	20.12.2016	Farm Bush	Stationary	permanent	M	7
SLP/MOY/194	SLE/ 194/16 SW			0,23	neg	South	Moyamba	kori	Taiama	Flock	Swine	Duroc	20.12.2016	Farm Bush	Stationary	permanent	M	7
SLP/MOY/195	SLE/ 195/16 SW			0,16	neg	South	Moyamba	kori	Taiama	Flock	Swine	Duroc	20.12.2016	Farm Bush	Stationary	permanent	M	7
SLP/MOY/196	SLE/ 196/16 SW			0,23	neg	South	Moyamba	kori	Taiama	Flock	Swine	Duroc	20.12.2016	Farm Bush	Stationary	permanent	M	7
SLP/MOY/197	SLE/ 197/16 SW			0,16	neg	South	Moyamba	kori	Taiama	Flock	Swine	Large White	20.12.2016	Farm Bush	Stationary	permanent	F	18
SLP/MOY/198	SLE/ 198/16 SW	Pool 40	neg	0,16	neg	South	Moyamba	kori	Taiama	Flock	Swine	Large White	20.12.2016	Farm Bush	Stationary	permanent	M	18
SLP/MOY/199	SLE/ 199/16 SW			0,21	neg	South	Moyamba	kori	Taiama	Flock	Swine	Large White	20.12.2016	Farm Bush	Stationary	permanent	F	18
SLP/MOY/200	SLE/ 200/16 SW			0,12	neg		Moyamba				Swine		27.12.2016				F	
1	SLE/ 201/16 SW			0,12	neg	Northern	Bombali	Makarie Gbanti	Panlap Village	pig farm	Swine	Exotic / Multiple crosses	19.12.2016	Farm bush near community	Stationary	Freetown	F	Adult (36)
2	SLE/ 202/16 SW			0,10	neg	Northern	Bombali	Makarie Gbanti	Panlap Village	pig farm	Swine	Duroc	19.12.2016	Farm bush near community	Stationary	Freetown	M	Grower (12)
3	SLE/ 203/16 SW	Pool 41	neg	0,09	neg	Northern	Bombali	Makarie Gbanti	Panlap Village	pig farm	Swine	Duroc	19.12.2016	Farm bush near community	Stationary	Freetown	F	Adult
4	SLE/ 204/16 SW			0,11	neg	Northern	Bombali	Makarie Gbanti	Panlap Village	pig farm	Swine	Duroc	19.12.2016	Farm bush near community	Stationary	Freetown	F	Gilt (8)
5	SLE/ 205/16 SW			0,17	neg	Northern	Bombali	Makarie Gbanti	Panlap Village	pig farm	Swine	Duroc	19.12.2016	Farm bush near community	Stationary	Freetown	F	Adult (36+)
6	SLE/ 206/16 SW			0,13	neg	Northern	Bombali	Makarie Gbanti	Panlap Village	pig farm	Swine	Duroc	19.12.2016	Farm bush near community	Stationary	Freetown	F	Adult
7	SLE/ 207/16 SW			0,10	neg	Northern	Bombali	Makarie Gbanti	Panlap Village	pig farm	Swine	Duroc	19.12.2016	Farm bush near community	Stationary	Freetown	F	Adult
8	SLE/ 208/16 SW	Pool 42	neg	0,16	neg	Northern	Bombali	Makarie Gbanti	Panlap Village	pig farm	Swine	Duroc	19.12.2016	Farm bush near community	Stationary	Freetown	F	Adult
9	SLE/ 209/16 SW			0,10	neg	Northern	Bombali	Makarie Gbanti	Panlap Village	pig farm	Swine	Large White Cross	19.12.2016	Farm bush near community	Stationary	Freetown	F	Adult
10	SLE/ 210/16 SW			0,09	neg	Northern	Bombali	Makarie Gbanti	Panlap Village	pig farm	Swine	Large White Cross	19.12.2016	Farm bush near community	Stationary	Freetown	M	Adult
11	SLE/ 211/16 SW			0,10	neg	Northern	Bombali	Makarie Gbanti	Panlap Village	pig farm	Swine	Large White Cross	19.12.2016	Farm bush near community	Stationary	Freetown	M	Adult
12	SLE/ 212/16 SW			0,21	neg	Northern	Bombali	Makarie Gbanti	Panlap Village	pig farm	Swine	Exotic Large White	19.12.2016	Farm bush near community	Stationary	Makeni town	F	Adult
13	SLE/ 213/16 SW	Pool 43	neg	0,09	neg	Northern	Bombali	Makarie Gbanti	Panlap Village	pig farm	Swine	Exotic Large White	19.12.2016	Farm bush near community	Stationary	Makeni town	F	Gilt
14	SLE/ 214/16 SW			0,13	neg	Northern	Bombali	Makarie Gbanti	Panlap Village	pig farm	Swine	Exotic Large White	19.12.2016	Farm bush near community	Stationary	Makeni town	F	Gilt
15	SLE/ 215/16 SW			0,20	neg	Northern	Bombali	Makarie Gbanti	Panlap Village	pig farm	Swine	Exotic Large White	19.12.2016	Farm bush near community	Stationary	Bo town	F	Adult
16	SLE/ 216/16 SW	Pool 44	neg	0,12	neg	Northern	Bombali	Makarie Gbanti	Panlap Village	pig farm	Swine	Exotic Large White	19.12.2016	Farm bush near community	Stationary	Bo town	M	Adult
17	SLE/ 217/16 SW			0,14	neg	Northern	Bombali	Makarie Gbanti	Panlap Village	pig farm	Swine	Exotic Large White	19.12.2016	Farm bush near community	Stationary	Bo town	F	Adult
18	SLE/ 218/16 SW			0,10	neg	Northern	Bombali	Sanda Tandaren	Rogbin Village	Pig farm	Swine	West African Dwarf(WAD)	20.12.2016	Farm bush near community	Stationary	brac	F	Adult
19	SLE/ 219/16 SW	Pool 44	neg	0,10	neg	Northern	Bombali	Sanda Tandaren	Rogbin Village	Pig farm	Swine	West African Dwarf(WAD)	20.12.2016	Farm bush near community	Stationary	brac	F	Adult
20	SLE/ 220/16 SW			0,18	neg	Northern	Bombali	Sanda Tandaren	Rogbin Village	Pig farm	Swine	West African Dwarf(WAD)	20.12.2016	Farm bush near community	Stationary	brac	M	Adult
21	SLE/ 221/16 SW			0,10	neg	Northern	Bombali	Sanda Tandaren	Rogbin Village	Pig farm	Swine	West African Dwarf(WAD)	20.12.2016	Farm bush near community	Stationary	brac	F	Gilt
22	SLE/ 222/16 SW			0,11	neg	Northern	Bombali	Sanda Tandaren	Rogbin Village	Pig farm	Swine	West African Dwarf(WAD)	20.12.2016	Farm bush near community	Stationary	brac	M	Grower
23	SLE/ 223/16 SW	Pool 45	neg	0,15	neg	Northern	Bombali	Sanda Tandaren	Rogbin Village	Pig farm	Swine	West African Dwarf(WAD)	20.12.2016	Farm bush near community	Stationary	brac	F	Adult
24	SLE/ 224/16 SW			0,09	neg	Northern	Bombali	Sanda Tandaren	Rogbin Village	Pig farm	Swine	West African Dwarf(WAD)	20.12.2016	Farm bush near community	Stationary	brac	F	Adult
25	SLE/ 225/16 SW			0,10	neg	Northern	Bombali	Sanda Tandaren	Rogbin Village	Pig farm	Swine	West African Dwarf(WAD)	20.12.2016	Farm bush near community	Stationary	brac	M	Adult
26	SLE/ 226/16 SW			0,20	neg	Northern	Bombali	Sanda Tandaren	Rogbin Village	Pig farm	Swine	Exotic Cross Breed	20.12.2016	Farm bush near community	Stationary	Lungi	M	Adult
27	SLE/ 227/16 SW			0,13	neg	Northern	Bombali	Sanda Tandaren	Rogbin Village	Pig farm	Swine	Large White Cross	20.12.2016	Farm bush near community	Stationary	Lungi	F	Adult
28	SLE/ 228/16 SW	Pool 46	neg	0,26	neg	Northern	Bombali	Sanda Tandaren	Rogbin Village	Pig farm	Swine	Large White Cross	20.12.2016	Farm bush near community	Stationary	Lungi	F	Adult
29	SLE/ 229/16 SW			0,34	neg	Northern	Bombali	Sanda Tandaren	Rogbin Village	Pig farm	Swine	Exotic Large White	20.12.2016	Farm bush near community	Stationary	Lungi	F	Adult
30	SLE/ 230/16 SW			0,19	neg	Northern	Bombali	Sanda Tandaren	Rogbin Village	Pig farm	Swine	Exotic Large White	20.12.2016	Farm bush near community	Stationary	Lungi	M	Adult
31	SLE/ 231/16 SW			0,12	neg	Northern	Bombali	Sanda Tandaren	Rogbin Village	Pig farm	Swine	Exotic Large White	20.12.2016	Farm bush near community	Stationary	Lungi	M	Adult
32	SLE/ 232/16 SW			0,22	neg	Northern	Bombali	Sanda Tandaren	Rogbin Village	Pig farm	Swine	Exotic Large White	20.12.2016	Farm bush near community	Stationary	Lungi	F	Adult
33	SLE/ 233/16 SW	Pool 47	neg	0,36	neg	Northern	Bombali	Sanda Tandaren	Rogbin Village	Pig farm	Swine	Exotic Large White	20.12.2016	Farm bush near community	Stationary	Lungi	F	Adult
34	SLE/ 234/16 SW			0,24	neg	Northern	Bombali	Sanda Tandaren	Rogbin Village	Pig farm	Swine	Duroc	20.12.2016	Farm bush near community	Stationary	Lungi	M	Adult
35	SLE/ 235/16 SW			0,20	neg	Northern	Bombali	Sanda Tandaren	Rogbin Village	Pig farm	Swine	Duroc	20.12.2016	Farm bush near community	Stationary	Lungi	F	Adult
**36**	SLE/ 236/16 SW			0,11	neg	Northern	Bombali	Gbanti Kamaranka	Kamaranka	Pig farm	Swine	West African Dwarf	20.12.2016	Community	Stationary	Kamalo	F	Adult
**37**	SLE/ 237/16 SW			0,09	neg	Northern	Bombali	Gbanti Kamaranka	Kamaranka	Pig farm	Swine	West African Dwarf	21.12.2016	Community	Stationary	Kamalo	M	Adult
**38**	SLE/ 238/16 SW	Pool 48	neg	0,14	neg	Northern	Bombali	Gbanti Kamaranka	Kamaranka	Pig farm	Swine	West African Dwarf	22.12.2016	Community	Stationary	Kamalo	F	Adult
**39**	SLE/ 239/16 SW			0,14	neg	Northern	Bombali	Gbanti Kamaranka	Kamaranka	Pig farm	Swine	West African Dwarf	23.12.2016	Community	Stationary	Kamalo	F	Adult
**40**	SLE/ 240/16 SW			0,09	neg	Northern	Bombali	Gbanti Kamaranka	Kamaranka	Pig farm	Swine	West African Dwarf	24.12.2016	Community	Stationary	Kamalo	F	Adult
**41**	SLE/ 241/16 SW					Northern	Bombali	Gbanti Kamaranka	Kamaranka	Pig farm	Swine	West African Dwarf	25.12.2016	Community	Stationary	Kamalo	M	Adult
**42**	SLE/ 242/16 SW			0,11	neg	Northern	Bombali	Gbanti Kamaranka	Kamaranka	Pig farm	Swine	West African Dwarf	26.12.2016	Community	Stationary	Kamalo	M	Adult
**43**	SLE/ 243/16 SW	Pool 49	neg	0,10	neg	Northern	Bombali	Gbanti Kamaranka	Kamaranka	Pig farm	Swine	West African Dwarf	27.12.2016	Community	Stationary	Kamalo	F	Adult
**44**	SLE/ 244/16 SW			0,10	neg	Northern	Bombali	Gbanti Kamaranka	Kamaranka	Pig farm	Swine	West African Dwarf	28.12.2016	Community	Stationary	Kamalo	M	Adult
**45**	SLE/ 245/16 SW			0,12	neg	Northern	Bombali	Gbanti Kamaranka	Kamaranka	Pig farm	Swine	West African Dwarf	29.12.2016	Community	Stationary	Kamalo	F	Adult
**46**	SLE/ 246/16 SW					Northern	Bombali	Gbanti Kamaranka	Kamaranka	Pig farm	Swine	West African Dwarf	30.12.2016	Community	Stationary	Kamalo	F	Adult
**47**	SLE/ 247/16 SW			0,11	neg	Northern	Bombali	Gbanti Kamaranka	Kamaranka	Pig farm	Swine	West African Dwarf	31.12.2016	Community	Stationary	Kamalo	F	Adult
**48**	SLE/ 248/16 SW	Pool 50	neg	0,12	neg	Northern	Bombali	Gbanti Kamaranka	Kamaranka	Pig farm	Swine	West African Dwarf	01.01.2017	Community	Stationary	Kamalo	F	Adult
**49**	SLE/ 249/16 SW			0,10	neg	Northern	Bombali	Gbanti Kamaranka	Kamaranka	Pig farm	Swine	West African Dwarf	02.01.2017	Community	Stationary	Kamalo	F	Adult
50	SLE/ 250/16 SW			0,09	neg	Northern	Port Loko	Buya Romende	Foredugu Village	Pig farm	Swine	Back Shire and Large White Cross	21.12.2016	Community	Stationary	Muraldo Farm	F	Adult
51	SLE/ 251/16 SW			0,09	neg	Northern	Port Loko	Buya Romende	Foredugu Village	Pig farm	Swine	Back Shire and Large White Cross	21.12.2016	Community	Stationary	Muraldo Farm	F	Adult
52	SLE/ 252/16 SW			0,10	neg	Northern	Port Loko	Buya Romende	Foredugu Village	Pig farm	Swine	Back Shire and Large White Cross	21.12.2016	Community	Stationary	Muraldo Farm	F	Adult
53	SLE/ 253/16 SW	Pool 51	neg	0,21	neg	Northern	Port Loko	Buya Romende	Foredugu Village	Pig farm	Swine	Back Shire and Large White Cross	21.12.2016	Community	Stationary	Muraldo Farm	F	Adult
54	SLE/ 254/16 SW			0,09	neg	Northern	Port Loko	Buya Romende	Foredugu Village	Pig farm	Swine	Back Shire and Large White Cross	21.12.2016	Community	Stationary	Muraldo Farm	M	Adult
55	SLE/ 255/16 SW			0,11	neg	Northern	Port Loko	Buya Romende	Foredugu Village	Pig farm	Swine	Back Shire and Large White Cross	21.12.2016	Community	Stationary	Muraldo Farm	F	Adult
56	SLE/ 256/16 SW			0,13	neg	Northern	Port Loko	Buya Romende	Foredugu Village	Pig farm	Swine	Back Shire and Large White Cross	21.12.2016	Community	Stationary	Muraldo Farm	M	Adult
57	SLE/ 257/16 SW			0,14	neg	Northern	Port Loko	Buya Romende	Foredugu Village	Pig farm	Swine	Back Shire and Large White Cross	21.12.2016	Community	Stationary	Muraldo Farm	M	Adult
58	SLE/ 258/16 SW	Pool 52	neg	0,09	neg	Northern	Port Loko	Buya Romende	Foredugu Village	Pig farm	Swine	Back Shire and Large White Cross	21.12.2016	Community	Stationary	Muraldo Farm	M	Adult
59	SLE/ 259/16 SW			0,13	neg	Northern	Port Loko	Buya Romende	Foredugu Village	Pig farm	Swine	Back Shire and Large White Cross	21.12.2016	Community	Stationary	Muraldo Farm	F	Adult
60	SLE/ 260/16 SW			0,12	neg	Northern	Port Loko	Buya Romende	Foredugu Village	Pig farm	Swine	Back Shire and Large White Cross	21.12.2016	Community	Stationary	Muraldo Farm	F	Adult
61	SLE/ 261/16 SW			0,10	neg	Northern	Port Loko	Buya Romende	Foredugu Village	Pig farm	Swine	Back Shire and Large White Cross	21.12.2016	Community	Stationary	Muraldo Farm	F	Adult
62	SLE/ 262/16 SW			0,10	neg	Northern	Port Loko	Buya Romende	Foredugu Village	Pig farm	Swine	Back Shire and Large White Cross	21.12.2016	Community	Stationary	Muraldo Farm	F	Grower
63	SLE/ 263/16 SW	Pool 53	neg	0,11	neg	Northern	Port Loko	Buya Romende	Foredugu Village	Pig farm	Swine	Back Shire and Large White Cross	21.12.2016	Community	Stationary	Muraldo Farm	M	Adult
64	SLE/ 264/16 SW			0,14	neg	Northern	Port Loko	Buya Romende	Foredugu Village	Pig farm	Swine	Back Shire and Large White Cross	21.12.2016	Community	Stationary	Muraldo Farm	M	Adult
65	SLE/ 265/16 SW			0,09	neg	Northern	Port Loko	Buya Romende	Foredugu Village	Pig farm	Swine	Back Shire and Large White Cross	21.12.2016	Community	Stationary	Muraldo Farm	M	Adult
66	SLE/ 266/16 SW			0,17	neg	Northern	Port Loko	Buya Romende	Foredugu Village	Pig farm	Swine	Durock Large White Cross	21.12.2016	Farm Bush	Stationary	Bumbuna	F	Grower
67	SLE/ 267/16 SW			0,17	neg	Northern	Port Loko	Buya Romende	Foredugu Village	Pig farm	Swine	Durock Large White Cross	21.12.2016	Secondary Forest	Stationary	Bumbuna	F	Adult
68	SLE/ 268/16 SW	Pool 54	neg	0,14	neg	Northern	Port Loko	Buya Romende	Foredugu Village	Pig farm	Swine	Durock Large White Cross	21.12.2016	Secondary Forest	Stationary	Bumbuna	F	1RR
69	SLE/ 269/16 SW			0,11	neg	Northern	Port Loko	Buya Romende	Foredugu Village	Pig farm	Swine	Back Shire and Large White Cross	21.12.2016	Secondary Forest	Stationary	Bumbuna	F	Adult
70	SLE/ 270/16 SW			0,11	neg	Northern	Port Loko	Buya Romende	Foredugu Village	Pig farm	Swine	Durock Large White Cross	21.12.2016	Secondary Forest	Stationary	BRAC	F	Adult
71	SLE/ 271/16 SW			0,12	neg	Northern	Port Loko	Buya Romende	Foredugu Village	Pig farm	Swine	Durock Large White Cross	21.12.2016	Secondary Forest	Stationary	BRAC	F	Grower
72	SLE/ 272/16 SW			0,10	neg	Northern	Port Loko	Buya Romende	Foredugu Village	Pig farm	Swine	Durock Large White Cross	21.12.2016	Secondary Forest	Stationary	BRAC	F	Grower
73	SLE/ 273/16 SW	Pool 55	neg	0,10	neg	Northern	Port Loko	Buya Romende	Foredugu Village	Pig farm	Swine	Durock Large White Cross	21.12.2016	Secondary Forest	Stationary	Muraldo Farm	M	Grower
74	SLE/ 274/16 SW			0,13	neg	Northern	Port Loko	Buya Romende	Foredugu Village	Pig farm	Swine	Durock Large White Cross	21.12.2016	Secondary Forest	Stationary	Muraldo Farm	M	Grower
75	SLE/ 275/16 SW			0,09	neg	Northern	Port Loko	Marampa	Lunsar Town (Old Town)	Pig farm	Swine	Back Shire and Large White Cross	21.12.2016	Inland Valley (Semi Intensive	Stationary	Muraldo Farm	F	Adult
76	SLE/ 276/16 SW			n/a	n/a	Northern	Port Loko	Marampa	Lunsar Town (Old Town)	Pig farm	Swine	Back Shire and Large White Cross	21.12.2016	Inland Valley (Semi Intensive	Stationary	Muraldo Farm	M	Adult
77	SLE/ 277/16 SW			0,09	neg	Northern	Port Loko	Marampa	Lunsar Town (Old Town)	Pig farm	Swine	Back Shire and Large White Cross	21.12.2016	Inland Valley (Semi Intensive	Stationary	Muraldo Farm	M	Adult
78	SLE/ 278/16 SW	Pool 56	neg	0,09	neg	Northern	Port Loko	Marampa	Lunsar Town (Old Town)	Pig farm	Swine	Back Shire and Large White Cross	21.12.2016	Inland Valley (Semi Intensive	Stationary	Muraldo Farm	F	Adult
79	SLE/ 279/16 SW			0,10	neg	Northern	Port Loko	Marampa	Lunsar Town (Old Town)	Pig farm	Swine	Back Shire and Large White Cross	21.12.2016	Inland Valley (Semi Intensive	Stationary	Muraldo Farm	F	Adult
80	SLE/ 280/16 SW			0,10	neg	Northern	Port Loko	Marampa	Lunsar Town (Old Town)	Pig farm	Swine	Back Shire and Large White Cross	21.12.2016	Inland Valley (Semi Intensive	Stationary	Muraldo Farm	F	Adult
81	SLE/ 281/16 SW			0,11	neg	Northern	Port Loko	Marampa	Lunsar Town (Old Town)	Pig farm	Swine	Durock	21.12.2016	Inland Valley (Semi Intensive	Stationary	Muraldo Farm	F	Adult
82	SLE/ 282/16 SW			0,20	neg	Northern	Port Loko	Marampa	Lunsar Town (Old Town)	Pig farm	Swine	Large White	21.12.2016	Inland Valley (Semi Intensive	Stationary	Muraldo Farm	F	Adult
83	SLE/ 283/16 SW	Pool 57	neg	0,11	neg	Northern	Port Loko	Marampa	Lunsar Town (Old Town)	Pig farm	Swine	Large White	21.12.2016	Inland Valley (Semi Intensive	Stationary	Muraldo Farm	F	Adult
84	SLE/ 284/16 SW			0,09	neg	Northern	Port Loko	Marampa	Lunsar Town (Old Town)	Pig farm	Swine	Large White	21.12.2016	Inland Valley (Semi Intensive	Stationary	Muraldo Farm	F	Adult
85	SLE/ 285/16 SW			0,10	neg	Northern	Port Loko	Marampa	Lunsar Town (Old Town)	Pig farm	Swine	Large White	21.12.2016	Inland Valley (Semi Intensive	Stationary	Muraldo Farm	F	Adult
86	SLE/ 286/16 SW			0,13	neg	Northern	Port Loko	Marampa	Lunsar Town (Old Town)	Pig farm	Swine	Large White	21.12.2016	Inland Valley (Semi Intensive	Stationary	Muraldo Farm	F	Adult
87	SLE/ 287/16 SW			0,16	neg	Northern	Port Loko	Marampa	Lunsar Town (Old Town)	Pig farm	Swine	Large White	21.12.2016	Inland Valley (Semi Intensive	Stationary	Muraldo Farm	F	Adult
88	SLE/ 288/16 SW	Pool 58	neg	0,10	neg	Northern	Port Loko	Marampa	Lunsar Town (Old Town)	Pig farm	Swine	Large White	21.12.2016	Inland Valley (Semi Intensive	Stationary	BRAC	F	Gilt

89	SLE/ 289/16 SW			0,15	neg	Northern	Port Loko	Marampa	Lunsar Town (Old Town)	Pig farm	Swine	Large White	21.12.2016	Inland Valley (Semi Intensive	Stationary	BRAC	F	Grower
90	SLE/ 290/16 SW			0,11	neg	Northern	Port Loko	Marampa	Lunsar Town (Old Town)	Pig farm	Swine	Large White	21.12.2016	Inland Valley (Semi Intensive	Stationary	BRAC	F	Grower
91	SLE/ 291/16 SW			0,14	neg	Northern	Port Loko	Marampa	Lunsar Town (Old Town)	Pig farm	Swine	Large White	21.12.2016	Inland Valley (Semi Intensive	Stationary	BRAC	F	Gilt
92	SLE/ 292/16 SW			0,08	neg	Northern	Port Loko	Marampa	Lunsar Town (Old Town)	Pig farm	Swine	Back Shire	21.12.2016	Community	Stationary	BRAC	F	Adult
93	SLE/ 293/16 SW	Pool 59	neg	0,09	neg	Northern	Port Loko	Marampa	Lunsar Town (Old Town)	Pig farm	Swine	Back Shire	21.12.2016	Community	Stationary	BRAC	M	Adult
94	SLE/ 294/16 SW			0,08	neg	Northern	Port Loko	Marampa	Lunsar Town (Old Town)	Pig farm	Swine	Large White	21.12.2016	Community	Stationary	BRAC	F	Gilt
95	SLE/ 295/16 SW			0,08	neg	Northern	Port Loko	Marampa	Lunsar Town (Old Town)	Pig farm	Swine	Large White	21.12.2016	Community	Stationary	BRAC	F	Gilt
96	SLE/ 296/16 SW			0,08	neg	Northern	Port Loko	Marampa	Lunsar Town (Old Town)	Pig farm	Swine	Large White Durock	21.12.2016	Community	Stationary	BRAC	F	Adult
97	SLE/ 297/16 SW			0,12	neg	Northern	Port Loko	Marampa	Lunsar Town (Old Town)	Pig farm	Swine	Large White	21.12.2016	Community	Stationary	Muraldo Farm	F	Adult
98	SLE/ 298/16 SW	Pool 60	neg	0,09	neg	Northern	Port Loko	Marampa	Lunsar Town (Old Town)	Pig farm	Swine	Large White	21.12.2016	Community	Stationary	Muraldo Farm	M	Adult
99	SLE/ 299/16 SW			0,09	neg	Northern	Port Loko	Marampa	Lunsar Town (Old Town)	Pig farm	Swine	Large White	21.12.2016	Community	Stationary	Muraldo Farm	M	Adult
100	SLE/ 300/16 SW			0,10	neg	Northern	Port Loko	Marampa	Lunsar Town (Old Town)	Pig farm	Swine	Large White	21.12.2016	Community	Stationary	Muraldo Farm	F	Adult
101	SLE/ 301/16 SW			0,10	neg	Northern	Port Loko	Marampa	Lunsar Town (Old Town)	Pig farm	Swine	Large White	21.12.2016	Community	Stationary	Muraldo Farm	F	Adult
102	SLE/ 302/16 SW			3,88	neg	Northern	Port Loko	Marampa	Lunsar Town (Old Town)	Pig farm	Swine	Large White	21.12.2016	Community	Stationary	Muraldo Farm	F	Adult
103	SLE/ 303/16 SW	Pool 61	neg	0,08	neg	Northern	Port Loko	Marampa	Lunsar Road Port Loko Town	pig farm	Swine	West African Dwarf	22.12.2016	Community	Free Range	Binkolo	F	Adult
104	SLE/ 304/16 SW			4,00	pos	Northern	Port Loko	Marampa	Lunsar Road Port Loko Town	pig farm	Swine	West African Dwarf	22.12.2016	Community	Free Range	Binkolo	F	Adult
105	SLE/ 305/16 SW			4,00	pos	Northern	Port Loko	Marampa	Lunsar Road Port Loko Town	pig farm	Swine	West African Dwarf	22.12.2016	Community	Free Range	Binkolo	F	Adult
106	SLE/ 306/16 SW			3,14	pos	Northern	Port Loko	Marampa	Lunsar Road Port Loko Town	pig farm	Swine	West African Dwarf	22.12.2016	Community	Free Range	Binkolo	M	Adult
107	SLE/ 307/16 SW			4,00	pos	Northern	Port Loko	Marampa	Lunsar Road Port Loko Town	pig farm	Swine	West African Dwarf	22.12.2016	Community	Free Range	Binkolo	F	Adult
108	SLE/ 308/16 SW	Pool 62	pos	3,95	pos	Northern	Port Loko	Marampa	Lunsar Road Port Loko Town	pig farm	Swine	West African Dwarf	22.12.2016	Community	Free Range	Binkolo	F	Adult
109	SLE/ 309/16 SW			4,00	pos	Northern	Port Loko	Marampa	Lunsar Road Port Loko Town	pig farm	Swine	West African Dwarf	22.12.2016	Community	Free Range	Binkolo	F	Adult
110	SLE/ 310/16 SW			4,00	pos	Northern	Port Loko	Marampa	Lunsar Road Port Loko Town	pig farm	Swine	West African Dwarf	22.12.2016	Community	Free Range	Binkolo	F	Gilt
111	SLE/ 311/16 SW			3,99	pos	Northern	Port Loko	Marampa	Lunsar Road Port Loko Town	pig farm	Swine	West African Dwarf	22.12.2016	Community	Free Range	Binkolo	M	Grower
112	SLE/ 312/16 SW			0,22	neg	Northern	Port Loko	Marampa	Lunsar Road Port Loko Town	pig farm	Swine	West African Dwarf	22.12.2016	Community	Free Range	Binkolo	F	Gilt
113	SLE/ 313/16 SW	Pool 63	pos	4,00	pos	Northern	Port Loko	Marampa	Lunsar Road Port Loko Town	pig farm	Swine	West African Dwarf	22.12.2016	Community	Free Range	Binkolo	F	Gilt
114	SLE/ 314/16 SW			0,75	pos	Northern	Port Loko	Marampa	Lunsar Road Port Loko Town	pig farm	Swine	West African Dwarf	22.12.2016	Community	Free Range	Freetown	F	Gilt
115	SLE/ 315/16 SW			0,16	neg	Northern	Port Loko	Marampa	Lunsar Road Port Loko Town	pig farm	Swine	Durock	22.12.2016	Community	Free Range	Freetown	M	Adult
116	SLE/ 316/16 SW			0,10	neg	Northern	Port Loko	Marampa	Lunsar Road Port Loko Town	pig farm	Swine	Large White	22.12.2016	Community	Free Range	Freetown	M	Adult
117	SLE/ 317/16 SW			0,17	neg	Northern	Port Loko	Marampa	Lunsar Road Port Loko Town	pig farm	Swine	Large White	22.12.2016	Community	Free Range	Freetown	F	Adult
118	SLE/ 318/16 SW	Pool 64	neg	4,00	pos	Northern	Port Loko	Marampa	Lunsar Road Port Loko Town	pig farm	Swine	Large White	22.12.2016	Community	Free Range	Freetown	F	Adult
119	SLE/ 319/16 SW			4,00	pos	Northern	Port Loko	Marampa	Lunsar Road Port Loko Town	pig farm	Swine	Durock	22.12.2016	Community	Free Range	Freetown	M	Adult
120	SLE/ 320/16 SW			0,11	neg	Northern	Port Loko	Marampa	Lunsar Road Port Loko Town	pig farm	Swine	Durock	22.12.2016	Community	Free Range	Freetown	M	Adult
121	SLE/ 321/16 SW			0,12	neg	Northern	Port Loko	Marampa	Lunsar Road Port Loko Town	pig farm	Swine	Durock	22.12.2016	Community	Free Range	Freetown	M	Adult
122	SLE/ 322/16 SW			3,63	pos	Northern	Port Loko	Marampa	Lunsar Road Port Loko Town	pig farm	Swine	Durock	22.12.2016	Community	Free Range	Freetown	F	Adult
123	SLE/ 323/16 SW	Pool 65	neg	0,98	pos	Northern	Port Loko	Marampa	Lunsar Town (Old Town)	Pig Farm	Swine	Large White	22.12.2016	Community	Stationary	Pepel	F	Adult
124	SLE/ 324/16 SW			0,23	neg	Northern	Port Loko	Marampa	Lunsar Town (Old Town)	Pig Farm	Swine	Large White	22.12.2016	Community	Stationary	Pepel	F	Adult
125	SLE/ 325/16 SW			0,09	neg	Northern	Port Loko	Marampa	Lunsar Town (Old Town)	Pig Farm	Swine	Large White	22.12.2016	Community	Stationary	Fanya Farm	M	Grower
126	SLE/ 326/16 SW			0,10	neg	Northern	Port Loko	Marampa	Lunsar Town (Old Town)	Pig Farm	Swine	Large White	22.12.2016	Community	Stationary	Fanya Farm	F	Gilt
127	SLE/ 327/16 SW			0,09	neg	Northern	Port Loko	Marampa	Lunsar Town (Old Town)	Pig Farm	Swine	Large White	22.12.2016	Community	Stationary	Fanya Farm	F	Gilt
128	SLE/ 328/16 SW	Pool 66	neg	0,12	neg	Northern	Port Loko	Marampa	Lunsar Town (Old Town)	Pig Farm	Swine	Large White	22.12.2016	Community	Stationary	Fanya Farm	F	Gilt
129	SLE/ 329/16 SW			0,12	neg	Northern	Port Loko	Marampa	Lunsar Town (Old Town)	Pig Farm	Swine	Large White	22.12.2016	Community	Stationary	Fanya Farm	F	Gilt
130	SLE/ 330/16 SW			0,12	neg	Northern	Port Loko	Marampa	Lunsar Town (Old Town)	Pig Farm	Swine	Large White	22.12.2016	Community	Stationary	Fanya Farm	M	Grower
131	SLE/ 331/16 SW			0,21	neg	Northern	Port Loko	Marampa	Lunsar Town (Old Town)	Pig Farm	Swine	Large White	22.12.2016	Community	Stationary	Fanya Farm	F	Adult
132	SLE/ 332/16 SW			0,09	neg	Northern	Port Loko	Marampa	Lunsar Town (Old Town)	Pig Farm	Swine	Large White	22.12.2016	Community	Stationary	Fanya Farm	F	Gilt
133	SLE/ 333/16 SW	Pool 67	neg	0,09	neg	Northern	Port Loko	Marampa	Lunsar Town (Old Town)	Pig Farm	Swine	Large White	22.12.2016	Community	Stationary	Fanya Farm	M	Grower
134	SLE/ 334/16 SW			0,09	neg	Northern	Port Loko	Marampa	Lunsar Town (Old Town)	Pig Farm	Swine	Large White	22.12.2016	Community	Stationary	Fanya Farm	M	Adult
135	SLE/ 335/16 SW			0,09	neg	Northern	Port Loko	Marampa	Lunsar Town (Old Town)	Pig Farm	Swine	Large White	22.12.2016	Community	Stationary	Fanya Farm	F	Adult
136	SLE/ 336/16 SW			0,10	neg	Northern	Port Loko	Marampa	Lunsar Town (Old Town)	Pig Farm	Swine	Large White	22.12.2016	Community	Stationary	Fanya Farm	M	Adult
137	SLE/ 337/16 SW			0,15	neg	Northern	Port Loko	Marampa	Lunsar Town (Old Town)	Pig Farm	Swine	Large White	22.12.2016	Community	Stationary	Fanya Farm	F	Gilt
138	SLE/ 338/16 SW	Pool 68	neg	0,12	neg	Northern	Port Loko	Marampa	Lunsar Town (Old Town)	Pig Farm	Swine	Large White	22.12.2016	Community	Stationary	Fanya Farm	F	Gilt
139	SLE/ 339/16 SW			0,15	neg	Northern	Port Loko	Marampa	Lunsar Town (Old Town)	Pig Farm	Swine	Large White	22.12.2016	Community	Stationary	Fanya Farm	F	Gilt
140	SLE/ 340/16 SW			0,11	neg	Northern	Port Loko	Marampa	Lunsar Town (Old Town)	Pig Farm	Swine	Large White	22.12.2016	Community	Stationary	Fanya Farm	F	Gilt
141	SLE/ 341/16 SW			0,12	neg	Northern	Port Loko	Marampa	Lunsar Town (Old Town)	Pig Farm	Swine	Large White	22.12.2016	Community	Stationary	Fanya Farm	M	Adult
142	SLE/ 342/16 SW			0,11	neg	Northern	Port Loko	Marampa	Lunsar Town (Old Town)	Pig Farm	Swine	Large White	22.12.2016	Community	Stationary	Fanya Farm	M	Grower
143	SLE/ 343/16 SW	Pool 69	neg	0,14	neg	Northern	Port Loko	Marampa	Lunsar Town (Old Town)	Pig Farm	Swine	Large White	22.12.2016	Community	Stationary	Brac	F	Gilt
144	SLE/ 344/16 SW			0,14	neg	Northern	Port Loko	Marampa	Lunsar Town (Old Town)	Pig Farm	Swine	Large White	22.12.2016	Community	Stationary	Brac	M	Grower
145	SLE/ 345/16 SW			0,12	neg	Northern	Port Loko	Marampa	Lunsar Town (Old Town)	Pig Farm	Swine	Large White	22.12.2016	Community	Stationary	Brac	F	Gilt
146	SLE/ 346/16 SW			0,11	neg	Northern	Port Loko	Marampa	Lunsar Town (Old Town)	Pig Farm	Swine	Large White	22.12.2016	Community	Stationary	Brac	F	Adult
147	SLE/ 347/16 SW			0,13	neg	Northern	Port Loko	Marampa	Lunsar Town (Old Town)	Pig Farm	Swine	Large White	22.12.2016	Community	Stationary	Brac	F	Adult
148	SLE/ 348/16 SW	Pool 70	neg	0,13	neg	Northern	Port Loko	Marampa	Lunsar Town (Old Town)	Pig Farm	Swine	Large White	22.12.2016	Community	Stationary	Brac	F	Adult
149	SLE/ 349/16 SW			0,14	neg	Northern	Port Loko	Marampa	Lunsar Town (Old Town)	Pig Farm	Swine	Large White	22.12.2016	Community	Stationary	Brac	F	Gilt
150	SLE/ 350/16 SW			0,10	neg	Northern	Port Loko	Marampa	Lunsar Town (Old Town)	Pig Farm	Swine	Large White	22.12.2016	Community	Stationary	Brac	M	Adult
151	SLE/ 351/16 SW					Northern	Port Loko	Marampa	Lunsar Town (Old Town)	Pig Farm	Swine	Large White	22.12.2016	Community	Stationary	Brac	F	Adult
152	SLE/ 352/16 SW			0,10	neg	Northern	Port Loko	Marampa	Lunsar Town (Old Town)	Pig Farm	Swine	Large White	22.12.2016	Community	Stationary	Brac	M	Adult
153	SLE/ 353/16 SW	Pool 71	neg	0,18	neg	Northern	Bombali	Sheborah	New York	Pig Farm	Swine	Cross of Duroc & Backshire	23.12.2016	Farm bush	Stationary	Makeni town	M	Grower
154	SLE/ 354/16 SW			0,14	neg	Northern	Bombali	Sheborah	New York	Pig Farm	Swine	"	23.12.2016	Farm bush near community	Stationary	Makeni town	M	Grower
155	SLE/ 355/16 SW			0,14	neg	Northern	Bombali	Sheborah	New York	Pig Farm	Swine	"	23.12.2016	Farm bush near community	Stationary	Makeni town	M	Adult
156	SLE/ 356/16 SW			0,10	neg	Northern	Bombali	Sheborah	New York	Pig Farm	Swine	"	23.12.2016	Farm bush near community	Stationary	Makeni town	F	Adult
157	SLE/ 357/16 SW			0,10	neg	Northern	Bombali	Sheborah	New York	Pig Farm	Swine	"	23.12.2016	Farm bush near community	Stationary	Makeni town	F	Gilt
158	SLE/ 358/16 SW	Pool 72	neg	0,20	neg	Northern	Bombali	Sheborah	New York	Pig Farm	Swine	"	23.12.2016	Farm bush near community	Stationary	Makeni town	F	Gilt
159	SLE/ 359/16 SW			0,19	neg	Northern	Bombali	Sheborah	New York	Pig Farm	Swine	"	23.12.2016	Farm bush near community	Stationary	Makeni town	F	Adult
160	SLE/ 360/16 SW			0,17	neg	Northern	Bombali	Sheborah	New York	Pig Farm	Swine	"	23.12.2016	Farm bush near community	Stationary	Makeni town	M	Adult
161	SLE/ 361/16 SW			0,12	neg	Northern	Bombali	Sheborah	New York	Pig Farm	Swine	"	23.12.2016	Farm bush near community	Stationary	Makeni town	M	Adult
162	SLE/ 362/16 SW			0,11	neg	Northern	Bombali	Sheborah	New York	Pig Farm	Swine	"	23.12.2016	Farm bush near community	Stationary	Makeni town	F	Adult
163	SLE/ 363/16 SW	Pool 73	neg	0,14	neg	Northern	Bombali	Sheborah	New York	Pig Farm	Swine	"	23.12.2016	Farm bush near community	Stationary	Makeni town	F	Adult
164	SLE/ 364/16 SW			0,11	neg	Northern	Bombali	Sheborah	New York	Pig Farm	Swine	Multiple Crosses	23.12.2016	Farm bush	Stationary	Waterloo	F	Adult
165	SLE/ 365/16 SW			0,09	neg	Northern	Bombali	Sheborah	New York	Pig Farm	Swine	Multiple Crosses	23.12.2016	Farm bush	Stationary	Waterloo	F	Adult
166	SLE/ 366/16 SW			0,10	neg	Northern	Bombali	Sheborah	New York	Pig Farm	Swine	Multiple Crosses	23.12.2016	Farm bush	Stationary	Waterloo	F	Adult
167	SLE/ 367/16 SW			0,09	neg	Northern	Bombali	Sheborah	New York	Pig Farm	Swine	Multiple Crosses	23.12.2016	Farm bush	Stationary	Waterloo	F	Adult
168	SLE/ 368/16 SW	Pool 74	Pool 74	0,10	neg	Northern	Bombali	Sheborah	New York	Pig Farm	Swine	Multiple Crosses	23.12.2016	Farm bush	Stationary	Waterloo	M	Adult
169	SLE/ 369/16 SW			0,11	neg	Northern	Bombali	Sheborah	New York	Pig Farm	Swine	Multiple Crosses	23.12.2016	Farm bush	Stationary	Waterloo	F	Adult
170	SLE/ 370/16 SW			0,13	neg	Northern	Bombali	Sheborah	New York	Pig Farm	Swine	Multiple Crosses	23.12.2016	Farm bush	Stationary	Waterloo	F	Gilt
171	SLE/ 371/16 SW			0,18	neg	Northern	Bombali	Sheborah	New York	Pig Farm	Swine	Multiple Crosses	23.12.2016	Farm bush	Stationary	Waterloo	F	Adult
172	SLE/ 372/16 SW			0,14	neg	Northern	Bombali	Sheborah	New York	Pig Farm	Swine	Multiple Crosses	23.12.2016	Farm bush	Stationary	Waterloo	F	Adult
173	SLE/ 373/16 SW	Pool 75	neg	0,11	neg	Northern	Bombali	Sheborah	New York	Pig Farm	Swine	Multiple Crosses	23.12.2016	Farm bush	Stationary	Waterloo	F	Adult
174	SLE/ 374/16 SW			0,14	neg	Northern	Bombali	Sheborah	New York	Pig Farm	Swine	Multiple Crosses	23.12.2016	Farm bush	Stationary	Waterloo	F	Adult
175	SLE/ 375/16 SW			0,11	neg	Northern	Bombali	Sheborah	New York	Pig Farm	Swine	Multiple Crosses	23.12.2016	Farm bush	Stationary	Waterloo	F	Gilt
176	SLE/ 376/16 SW			0,15	neg	Northern	Bombali	Sheborah	New York	Pig Farm	Swine	Multiple Crosses	23.12.2016	Farm bush	Stationary	Waterloo	F	Adult
177	SLE/ 377/16 SW			0,12	neg	Northern	Bombali	Sheborah	New York	Pig Farm	Swine	Multiple Crosses	23.12.2016	Farm bush	Stationary	Waterloo	F	Adult
178	SLE/ 378/16 SW	Pool 76	neg	0,12	neg	Northern	Bombali	Sheborah	New York	Pig Farm	Swine	Multiple Crosses	23.12.2016	Farm bush	Stationary	Waterloo	F	Adult
179	SLE/ 379/16 SW			0,16	neg	Northern	Bombali	Sheborah	New York	Pig Farm	Swine	Multiple Crosses	23.12.2016	Farm bush	Stationary	Waterloo	F	Adult
180	SLE/ 380/16 SW			n/a	n/a	Northern	Bombali	Sheborah	New York	Pig Farm	Swine	Multiple Crosses	23.12.2016	Farm bush	Stationary	Waterloo	F	Adult
181	SLE/ 381/16 SW			0,10	neg	Northern	Bombali	Sheborah	New York	Pig Farm	Swine	Multiple Crosses	23.12.2016	Farm bush	Stationary	Waterloo	M	Adult
182	SLE/ 382/16 SW			0,10	neg	Northern	Bombali	Sheborah	New York	Pig Farm	Swine	Multiple Crosses	23.12.2016	Farm bush	Stationary	Waterloo	F	Adult
183	SLE/ 383/16 SW	Pool 77	neg	0,09	neg	Northern	Bombali	Sheborah	New York	Pig Farm	Swine	Multiple Crosses	23.12.2016	Farm bush	Stationary	Waterloo	M	Adult
184	SLE/ 384/16 SW			0,09	neg	Northern	Bombali	Sheborah	New York	Pig Farm	Swine	Multiple Crosses	23.12.2016	Farm bush	Stationary	Waterloo	F	Adult
185	SLE/ 385/16 SW			0,10	neg	Northern	Bombali	Sheborah	New York	Pig Farm	Swine	Multiple Crosses	23.12.2016	Farm bush	Stationary	Waterloo	F	Adult

186	SLE/ 386/16 SW			0,11	neg	Northern	Bombali	Sheborah	New York	Pig Farm	Swine	Multiple Crosses	23.12.2016	Farm bush	Stationary	Waterloo	M	Adult
187	SLE/ 387/16 SW			0,11	neg	Northern	Bombali	Sheborah	New York	Pig Farm	Swine	Multiple Crosses	23.12.2016	Farm bush	Stationary	Waterloo	F	Adult
188	SLE/ 388/16 SW	Pool 78	neg	0,13	neg	Northern	Bombali	Sheborah	New York	Pig Farm	Swine	Multiple Crosses	23.12.2016	Farm bush	Stationary	Waterloo	F	Adult
189	SLE/ 389/16 SW			0,10	neg	Northern	Bombali	Sheborah	New York	Pig Farm	Swine	Multiple Crosses	23.12.2016	Farm bush	Stationary	Waterloo	F	Adult
190	SLE/ 390/16 SW			0,12	neg	Northern	Bombali	Sheborah	New York	Pig Farm	Swine	Multiple Crosses	23.12.2016	Farm bush	Stationary	Waterloo	F	Adult
191	SLE/ 391/16 SW			0,09	neg	Northern	Bombali	Sheborah	New York	Pig Farm	Swine	Multiple Crosses	23.12.2016	Farm bush	Stationary	Waterloo	F	Adult
192	SLE/ 392/16 SW			0,10	neg	Northern	Bombali	Sheborah	New York	Pig Farm	Swine	Multiple Crosses	23.12.2016	Farm bush	Stationary	Waterloo	F	Adult
193	SLE/ 393/16 SW	Pool 79	neg	0,09	neg	Bombali/ Northern Region	Pig Farm	Sheborah	New York	Pig Farm	Swine	Multiple Crosses	24.12.2016	Community	Stationary	Waterloo	F	Adult
194	SLE/ 394/16 SW			0,09	neg	Bombali/ Northern Region	Pig Farm	Sheborah	New York	Pig Farm	Swine	Multiple Crosses	24.12.2016	Community	Stationary	Waterloo	F	Adult
195	SLE/ 395/16 SW			0,13	neg	Bombali/ Northern Region	Pig Farm	Sheborah	New York	Pig Farm	Swine	Multiple Crosses	24.12.2016	Community	Stationary	Waterloo	F	Adult
196	SLE/ 396/16 SW			0,14	neg	Bombali/ Northern Region	Pig Farm	Sheborah	New York	Pig Farm	Swine	Multiple Crosses	24.12.2016	Community	Stationary	Waterloo	F	Adult
197	SLE/ 397/16 SW			0,12	neg	Bombali/ Northern Region	Pig Farm	Sheborah	New York	Pig Farm	Swine	Multiple Crosses	24.12.2016	Community	Stationary	Waterloo	F	Adult
198	SLE/ 398/16 SW	Pool 80	neg	0,09	neg	Bombali/ Northern Region	Pig Farm	Sheborah	New York	Pig Farm	Swine	Multiple Crosses	24.12.2016	Community	Stationary	Waterloo	M	Grower
199	SLE/ 399/16 SW			0,08	neg	Bombali/ Northern Region	Pig Farm	Sheborah	New York	Pig Farm	Swine	Multiple Crosses	24.12.2016	Community	Stationary	Waterloo	F	Grower
200	SLE/ 400/16 SW			0,07	neg	Bombali/ Northern Region	Pig Farm	Sheborah	New York	Pig Farm	Swine	Multiple Crosses	24.12.2016	Community	Stationary	Waterloo	M	Grower

2017

Africa No.	Designation FLI	Serum-Pool Nr	Findings (PCR)	OD405	Finding	***Region/ District***	***Herd /Flock/ Farm***	***Animal Species***	***Breed***	***Habitat Description***	***Management System***	***Housing, Stable,Pasture, Stationary,Nomadic***	***History of Migration***	***Gender Male/Female***	***Age (Months)***	***Type of Specimen***
1	SLE/901/17 SW			0,82	pos	Northern region	Pig Farm	Pig	West Africa Duwarf	Community close to Farmbush	Intensive Care	Nomadic	Lungi Town	Male	Adult (1yr+)	Serum
2	SLE/902/17 SW			0,91	pos	Northern region	Pig Farm	Pig	West Africa Duwarf	Community close to Farmbush	" "	Nomadic	Lungi Town	Female	Adult (1yr+)	Serum
3	SLE/903/17 SW	Pool 101	neg	0,13	neg	Northern region	Pig Farm	Pig	West Africa Duwarf	Community close to Farmbush	" "	Nomadic	Freetown	Male	Adult (1yr)	Serum
4	SLE/904/17 SW			0,12	neg	Northern region	Pig Farm	Pig	West Africa Duwarf	Community close to Farmbush	" "	Nomadic	Freetown	Male	Adult (1yr)	Serum
5	SLE/905/17 SW			0,17	neg	Northern region	Pig Farm	Pig	West Africa Duwarf	Community close to Farmbush	" "	Nomadic	Freetown	Male	Adult (1yr)	Serum
6	SLE/906/17 SW			0,11	neg	Northern region	Pig Farm	Pig	West Africa Duwarf	Community close to Farmbush	" "	Nomadic	Freetown	Male	Adult (2yrs+)	Serum
7	SLE/907/17 SW			0,14	neg	Northern region	Pig Farm	Pig	West Africa Duwarf	Community close to Farmbush	" "	Nomadic	Freetown	Male	Adult	Serum
8	SLE/908/17 SW	Pool 102	neg	0,12	neg	Northern region	Pig Farm	Pig	West Africa Duwarf	Community close to Farmbush	" "	Nomadic	Freetown	Male	Grower 6-7Months	Serum
9	SLE/909/17 SW			0,14	neg	Northern region	Pig Farm	Pig	West Africa Duwarf	Community close to Farmbush	" "	Nomadic	Freetown	Female	Gilt 8months	Serum
10	SLE/910/17 SW			0,11	neg	Northern region	Pig Farm	Pig	West Africa Duwarf	Community close to Farmbush	" "	Nomadic	Freetown	Female	Gilt 8months	Serum
11	SLE/911/17 SW			0,20	neg	Northern/PortLoko	Pig farm	Pig	Improved	Community close tosecondary forest	Free range	Nomadic	Freetown	Male	7-8 months	Serum
12	SLE/912/17 SW			0,11	neg	Northern/PortLoko	Pig farm	Pig	Improved	Community close tosecondary forest	"	Nomadic	Freetown	Female	1yr+	Serum
13	SLE/913/17 SW	Pool 103	neg	0,13	neg	Northern/PortLoko	Pig farm	Pig	Improved	Community close tosecondary forest	"	Nomadic	Freetown	Female	Adult	Serum
14	SLE/914/17 SW			0,13	neg	Northern/PortLoko	Pig farm	Pig	Improved	Community close tosecondary forest	"	Nomadic	Freetown	Male	1 yr	Serum
15	SLE/915/17 SW			0,17	neg	Northern/PortLoko	Pig farm	Pig	Improved	Community close tosecondary forest	"	Nomadic	Freetown	Male	3yrs	Serum
16	SLE/916/17 SW			0,09	neg	Northern/PortLoko	Pig farm	Pig	Improved	Community close tosecondary forest	"	Nomadic	Freetown	Female	2yrs	Serum
17	SLE/917/17 SW			0,12	neg	Northern/PortLoko	Pig farm	Pig	Improved	Community close tosecondary forest	"	Nomadic	Freetown	Male	1 yr	Serum
18	SLE/918/17 SW	Pool 104	neg	0,10	neg	Northern/PortLoko	Pig farm	Pig	Improved	Community close tosecondary forest	"	Nomadic	Freetown	Female	3yrs	Serum
19	SLE/919/17 SW			0,08	neg	Northern/PortLoko	Pig farm	Pig	West African Dwarf(WAD)	Community close tosecondary forest	"	Nomadic	Freetown	Male	8 Months	Serum
20	SLE/920/17 SW			0,11	neg	Northern/PortLoko	Pig farm	Pig	West African Dwarf(WAD)	Community close tosecondary forest	"	Nomadic	Freetown	Male	9 Months	Serum
21	SLE/921/17 SW			0,11	neg	Northern/PortLoko	Pig farm	Pig	West African Dwarf(WAD)	Community close tosecondary forest	"	Nomadic	Freetown	Male	Boar (3 yrs)	Serum
22	SLE/922/17 SW			0,09	neg	Northern/PortLoko	Pig farm	Pig	West African Dwarf(WAD)	Community close tosecondary forest	"	Nomadic	Freetown	Male	Boar (3 yrs)	Serum
23	SLE/923/17 SW	Pool 105	neg	0,11	neg	Northern/PortLoko	Pig farm	Pig	Crosses	Community close tosecondary forest	"	Nomadic	Freetown	Male	Boar (3yrs +)	Serum
24	SLE/924/17 SW			0,18	neg	Northern/PortLoko	Pig farm	Pig	Crosses	Community close tosecondary forest	"	Nomadic	Freetown	Female	3 yrs	Serum
25	SLE/925/17 SW			0,37	pos	Northern/PortLoko	Pig farm	Pig	Crosses	Community close tosecondary forest	"	Nomadic	Freetown	Female	3 yrs	Serum
26	SLE/926/17 SW			0,12	neg	Northern/PortLoko	Pig farm	Pig	Crosses	Community close tosecondary forest	"	Nomadic	Freetown	Male	Boar 4 yrs	Serum
27	SLE/927/17 SW			0,11	neg	Northern/PortLoko	Pig farm	Pig	Crosses	Community close tosecondary forest	"	Nomadic	Freetown	Female	3 yrs	Serum
28	SLE/928/17 SW	Pool 106	neg	0,13	neg	Northern/PortLoko	Pig farm	Pig	Crosses	Community close tosecondary forest	"	Nomadic	Freetown	Male	3 yrs	Serum
29	SLE/929/17 SW			0,11	neg	Northern/PortLoko	Pig farm	Pig	Crosses	Community close tosecondary forest	"	Nomadic	Freetown	Female	2 yrs	Serum
30	SLE/930/17 SW			0,10	neg	Northern/PortLoko	Pig farm	Pig	Crosses	Community close tosecondary forest	"	Nomadic	Freetown	Male	1 yrs +	Serum
31	SLE/931/17 SW			0,10	neg	Northern/PortLoko	Pig farm	Swine	Large White	Community close to farm bush	Intensive Care	Housing	Freetown	Male	10 Months	Serum
32	SLE/932/17 SW			0,12	neg	Northern/PortLoko	Pig farm	Swine	Large White	Community close to farm bush	Intensive Care	Housing	Freetown	Male	8 Months	Serum
33	SLE/933/17 SW	Pool 107	neg	0,12	neg	Northern/PortLoko	Pig farm	Swine	Large White	Community close to farm bush	Intensive Care	Housing	Freetown	Male	9 Months	Serum
34	SLE/934/17 SW			0,11	neg	Northern/PortLoko	Pig farm	Swine	Large White	Community close to farm bush	Intensive Care	Housing	Freetown	Male	10 Months	Serum
35	SLE/935/17 SW			0,10	neg	Northern/PortLoko	Pig farm	Swine	Large White	Community close to farm bush	Intensive Care	Housing	Freetown	Male	8 Months	Serum
36	SLE/936/17 SW			0,12	neg	Northern/PortLoko	Pig farm	Swine	Large White	Community close to farm bush	Intensive Care	Housing	Freetown	Male	7 Months	Serum
37	SLE/937/17 SW			0,15	neg	Northern/PortLoko	Pig farm	Swine	Large White-Duroc	Community close to farm bush	Intensive Care	Housing	Freetown	Male	7 Months	Serum
38	SLE/938/17 SW	Pool 108	neg	0,09	neg	Northern/PortLoko	Pig farm	Swine	Large White-Duroc	Community close to farm bush	Intensive Care	Housing	Freetown	Male	9 Months	Serum
39	SLE/939/17 SW			0,15	neg	Northern/PortLoko	Pig farm	Swine	Large White-Duroc	Community close to farm bush	Intensive Care	Housing	Freetown	Male	9 Months	Serum
40	SLE/940/17 SW			0,13	neg	Northern/PortLoko	Pig farm	Swine	Landrace-WAD	Community close to farm bush	Intensive Care	Housing	Freetown	Male	1 yr +	Serum
41	SLE/941/17 SW			0,12	neg	Northern/PortLoko	Pig farm	Swine	Large White-Duroc	Community close to farm bush	Intensive Care	Housing	Freetown	Male	10 Months	Serum
42	SLE/942/17 SW			0,13	neg	Northern/PortLoko	Pig farm	Swine	Large White-Duroc	Community close to farm bush	Intensive Care	Housing	Lunsar	Male	10 Months	Serum
43	SLE/943/17 SW	Pool 109	neg	0,12	neg	Northern/PortLoko	Pig farm	Swine	Large White-Duroc	Community close to farm bush	Intensive Care	Housing	Lunsar	Male	1yr+	Serum
44	SLE/944/17 SW			0,12	neg	Northern/PortLoko	Pig farm	Swine	Large White-Duroc	Community close to farm bush	Intensive Care	Housing	Lunsar	Male	1yr+	Serum
45	SLE/945/17 SW			0,99	pos	Northern/PortLoko	Pig farm	Swine	Large White-Duroc	Community close to farm bush	Intensive Care	Housing	Lunsar	Male	1yr+	Serum
46	SLE/946/17 SW			0,13	neg	Northern/PortLoko	Pig farm	Swine	Large White-Duroc	Community close to farm bush	Intensive Care	Housing	Lunsar	Male	1yr+	Serum
47	SLE/947/17 SW			0,11	neg	Northern/PortLoko	Pig farm	Swine	Large White-Duroc	Community close to farm bush	Intensive Care	Housing	Lunsar	Male	1yr+	Serum
48	SLE/948/17 SW	Pool 110	neg	0,23	neg	Northern/PortLoko	Pig farm	Swine	Large White-WAD	Community close to farm bush	Intensive Care	Housing	Lunsar	Male	1yr+	Serum
49	SLE/949/17 SW			0,16	neg	Northern/PortLoko	Pig farm	Swine	Large White-WAD	Community close to farm bush	Intensive Care	Housing	Lunsar	Male	8 Months	Serum
50	SLE/950/17 SW			0,11	neg	Northern/PortLoko	Pig farm	Swine	Large White-WAD	Community close to farm bush	Intensive Care	Housing	Lunsar	Male	8 Months	Serum
51	SLE/951/17 SW			0,09	neg	Northern/PortLoko	Pig farm	Swine	Large White-WAD	Community close to farm bush	Intensive Care	Housing	Lungi Town	Male	8 Months	Serum
52	SLE/952/17 SW			0,09	neg	Northern/PortLoko	Pig farm	Swine	Large White-WAD	Community close to farm bush	Intensive Care	Housing	Lungi Town	Male	8Months	Serum
53	SLE/953/17 SW	Pool 111	neg	0,16	neg	Northern/PortLoko	Pig farm	Swine	Large White-WAD	Community close to farm bush	Intensive Care	Housing	Lungi Town	Female	6-7 Months	Serum
54	SLE/954/17 SW			0,13	neg	Northern/PortLoko	Pig farm	Swine	Large White-WAD	Community close to farm bush	Intensive Care	Housing	Lungi Town	Male	10 Months	Serum
55	SLE/955/17 SW			0,11	neg	Northern/PortLoko	Pig farm	Swine	Large White-WAD	Community close to farm bush	Intensive Care	Housing	Lungi Town	Female	2 yrs +	Serum
56	SLE/956/17 SW			0,11	neg	Northern/PortLoko	Pig farm	Swine	Large White-WAD	Community close to farm bush	Intensive Care	Housing	Lungi Town	Male	1 yr +	Serum
57	SLE/957/17 SW			0,14	neg	Northern/PortLoko	Pig farm	Swine	Large White-Duroc	Community close to farm bush	Intensive Care	Housing	Lungi Town	Male	1 yr +	Serum
58	SLE/958/17 SW	Pool 112	neg	0,12	neg	Northern/PortLoko	Pig farm	Swine	Large White-Duroc	Community close to farm bush	Intensive Care	Housing	Lungi Town	Male	1yr+	Serum
59	SLE/959/17 SW			0,10	neg	Northern/PortLoko	Pig farm	Swine	Large White-Duroc	Community close to farm bush	Intensive Care	Housing	Lungi Town	Female	Gilt 5-6Months	Serum
60	SLE/960/17 SW			0,10	neg	Northern/PortLoko	Pig farm	Swine	Large White-Duroc	Community close to farm bush	Intensive Care	Housing	Lungi Town	Female	Gilt 5-6Months	Serum
61	SLE/961/17 SW			0,08	neg	Northern/PortLoko	Pig farm	Swine	Large White-Duroc	Community close to farm bush	Intensive Care	Housing	Lungi Town	Male	Grower	Serum
62	SLE/962/17 SW			0,09	neg	Northern/PortLoko	Pig farm	Swine	Large White-Duroc	Community close to farm bush	Intensive Care	Housing	Lungi Town	Female	Gilt 5-6Months	Serum
63	SLE/963/17 SW	Pool 113	neg	0,11	neg	Northern/PortLoko	Pig farm	Swine	Large White-Duroc	Community close to farm bush	Intensive Care	Housing	Lungi Town	Female	Gilt 5-6Months	Serum
64	SLE/964/17 SW			0,13	neg	Northern/PortLoko	Pig farm	Swine	Large White-Duroc	Community close to farm bush	Intensive Care	Housing	Lungi Town	Female	Gilt 5-6Months	Serum
65	SLE/965/17 SW			0,11	neg	Northern/PortLoko	Pig farm	Swine	Large White-Duroc	Community close to farm bush	Intensive Care	Housing	Lungi Town	Female	Gilt 5-6Months	Serum
66	SLE/966/17 SW			0,09	neg							Housing	Lungi Town	Male	1 yr +	Serum
67	SLE/967/17 SW			0,08	neg							Housing	Lungi Town	Female	2 yrs +	Serum
68	SLE/968/17 SW	Pool 114	neg	0,09	neg							Housing	Lungi Town	Male	1 yr +	Serum
69	SLE/969/17 SW			0,09	neg							Housing	Lungi Town	Male	1 yr +	Serum
70	SLE/970/17 SW			0,09	neg							Housing	Lungi Town	Female	3 yrs+	Serum
71	SLE/971/17 SW			0,11	neg	Northern/PortLoko	Pig farm	Swine	West Africa Duwarf	Community close to farm bush	Semi Intensive Care	Nomadic	Lungi Town	Female	6 Months	Serum
72	SLE/972/17 SW			0,12	neg	Northern/PortLoko	Pig farm	Swine	West Africa Duwarf	Community close to farm bush	"	Nomadic	Lungi Town	Male	12Months	Serum
73	SLE/973/17 SW	Pool 115	neg	0,11	neg	Northern/PortLoko	Pig farm	Swine	West Africa Duwarf	Community close to farm bush	"	Nomadic	Lungi Town	Male	2yrs +	Serum
74	SLE/974/17 SW			0,10	neg	Northern/PortLoko	Pig farm	Swine	West Africa Duwarf	Community close to farm bush	"	Nomadic	Lungi Town	Female	3 yrs +	Serum
75	SLE/975/17 SW			0,11	neg	Northern/PortLoko	Pig farm	Swine	West Africa Duwarf	Community close to farm bush	"	Nomadic	Lungi Town	Female	Guilt	Serum
76	SLE/976/17 SW			0,11	neg	Northern/PortLoko	Pig farm	Swine	West Africa Duwarf	Community close to farm bush	"	Nomadic	Portloko Town	Female	6-8 Months	Serum

77	SLE/977/17 SW			0,09	neg	Northern/PortLoko	Pig farm	Swine	West Africa Duwarf	Community close to farm bush	"	Nomadic	Portloko Town	Female	6 Months	Serum
78	SLE/978/17 SW	Pool 116	neg	0,09	neg	Northern/PortLoko	Pig farm	Swine	West Africa Duwarf	Community close to farm bush	"	Nomadic	Portloko Town	Female	1 yr	Serum
79	SLE/979/17 SW			0,11	neg	Northern/PortLoko	Pig farm	Swine	West Africa Duwarf	Community close to farm bush	"	Nomadic	Portloko Town	Male	2 yrs +	Serum
80	SLE/980/17 SW			0,12	neg	Northern/Portloko	Pig farm	Swine	West African Duwarf	Community close to farm bush	Semi Intensive Care	Nomadic	Lunsar town	Male	Adult (1yr)	Serum
81	SLE/981/17 SW			0,11	neg	Northern/Portloko	Pig farm	Swine	West African Duwarf	Community close to farm bush	Semi Intensive Care	Nomadic	Lunsar town	Female	1n yr +	Serum
82	SLE/982/17 SW			0,13	neg	Northern/Portloko	Pig farm	Swine	West African Duwarf	Community close to farm bush	Semi Intensive Care	Nomadic	Lunsar town	Male	8 Months	Serum
83	SLE/983/17 SW	Pool 117	neg	0,12	neg	Northern/Portloko	Pig farm	Swine	West African Duwarf	Community close to farm bush	Semi Intensive Care	Nomadic	Lunsar town	Male	8 Months	Serum
84	SLE/984/17 SW			0,12	neg	Northern/Portloko	Pig farm	Swine	West African Duwarf	Community close to farm bush	Semi Intensive Care	Nomadic	Lunsar town	Female	Gilt (7 Months)	Serum
85	SLE/985/17 SW			0,10	neg	Northern/Portloko	Pig farm	Swine	West African Duwarf	Community close to farm bush	Semi Intensive Care	Nomadic	Lunsar town	Male	7 Months	Serum
86	SLE/986/17 SW			0,10	neg	Northern/Portloko	Pig farm	Swine	West African Duwarf	Community close to farm bush	Semi Intensive Care	Nomadic	Lunsar town	Male	Adult (1yr +)	Serum
87	SLE/987/17 SW			0,14	neg	Northern/Portloko	Pig farm	Swine	West African Duwarf	Community close to farm bush	Semi Intensive Care	Nomadic	Lunsar town	Female	2 yrs +	Serum
88	SLE/988/17 SW	Pool 118	neg	0,14	neg	Northern/Portloko	Pig farm	Swine	West African Duwarf	Community close to farm bush	Semi Intensive Care	Nomadic	Lunsar town	Female	2 yrs +	Serum
89	SLE/989/17 SW			0,26	neg	Northern/Portloko	Pig farm	Swine	West African Duwarf	Community close to farm bush	Semi Intensive Care	Nomadic	Lunsar town	Female	2 yrs	Serum
90	SLE/990/17 SW			0,12	neg	Northern/Portloko	Pig farm	Swine	West African Duwarf	Community close to farm bush	Semi Intensive Care	Nomadic	Lunsar town	Female	3 yrs +	Serum
91	SLE/991/17 SW			0,27	neg	Northern/Portloko	Pig farm	Swine	West African Duwarf	Community close to farm bush	Semi Intensive Care	Nomadic	Lunsar town	Female	10 Months	Serum
92	SLE/992/17 SW			0,82	pos	Northern/Portloko	Pig farm	Swine	West African Duwarf	Community close to farm bush	Semi Intensive Care	Nomadic	Lunsar town	Female	3 yrs	Serum
93	SLE/993/17 SW	Pool 119	neg	0,34	pos	Northern/Portloko	Pig farm	Swine	West African Duwarf	Community close to farm bush	Semi Intensive Care	Nomadic	Lunsar town	Female	1 yr +	Serum
94	SLE/994/17 SW			0,24	neg	Northern/Portloko	Pig farm	Swine	West African Duwarf	Community close to farm bush	Semi Intensive Care	Nomadic	Lunsar town	Male	6 Months	Serum
95	SLE/995/17 SW			0,12	neg	Northern/Portloko	Pig farm	Swine	West African Duwarf	Community close to farm bush	Semi Intensive Care	Nomadic	Lunsar town	Female	1 yr +	Serum
96	SLE/996/17 SW			0,88	pos	Northern/Portloko	Pig farm	Swine	West African Duwarf	Community close to farm bush	Semi Intensive Care	Nomadic	Lunsar town	Female	1 yr +	Serum
97	SLE/997/17 SW			0,28	inkl	Northern/Bombali	Pig farm	Swine	West African Duwarf	Community close to farm bush	Semi Intensive Care	Nomadic	Kamaranka Town	Male	6 Months	Serum
98	SLE/998/17 SW	Pool 120	neg	0,22	neg	Northern/Bombali	Pig farm	Swine	West African Duwarf	Community close to farm bush	Semi Intensive Care	Nomadic	Kamaranka Town	Male	6 Months	Serum
99	SLE/999/17 SW			0,44	pos	Northern/Bombali	Pig farm	Swine	West African Duwarf	Community close to farm bush	Semi Intensive Care	Nomadic	Kamaranka Town	Female	6 Months	Serum
100	SLE/1000/17 SW			0,22	neg	Northern/Bombali	Pig farm	Swine	West African Duwarf	Community close to farm bush	Semi Intensive Care	Nomadic	Kamaranka Town	Female	6 Months	Serum
101	SLE/1001/17 SW			0,12	neg	Northern/Bombali	Pig farm	Swine	West African Duwarf	Community close to farm bush	Semi Intensive Care	Nomadic	Kamaranka Town	Female	2 yrs +	Serum
102	SLE/1002/17 SW			0,14	neg	Northern/Bombali	Pig farm	Swine	West African Duwarf	Community close to farm bush	Semi Intensive Care	Nomadic	Kamaranka Town	Female	1 yr +	Serum
103	SLE/1003/17 SW	Pool 121	neg	0,13	neg	Northern/Bombali	Pig farm	Swine	West African Duwarf	Community close to farm bush	Semi Intensive Care	Nomadic	Kamaranka Town	Male	1 yr +	Serum
104	SLE/1004/17 SW			0,14	neg	Northern/Bombali	Pig farm	Swine	West African Duwarf	Community close to farm bush	Semi Intensive Care	Nomadic	Kamaranka Town	Male	1 yr +	Serum
105	SLE/1005/17 SW			0,19	neg	Northern/Bombali	Pig farm	Swine	West African Duwarf	Community close to farm bush	Semi Intensive Care	Nomadic	Kamaranka Town	Female	1 yr +	Serum
106	SLE/1006/17 SW			0,17	neg	Northern/Bombali	Pig farm	Swine	West African Duwarf	Community close to farm bush	Semi Intensive Care	Nomadic	Kamaranka Town	Female	1 yr +	Serum
107	SLE/1007/17 SW			0,17	neg	Northern/Bombali	Pig farm	Swine	West African Duwarf	Community close to farm bush	Semi Intensive Care	Nomadic	Freetown	Female	8 Months	Serum
108	SLE/1008/17 SW	Pool 122	neg	0,19	neg	Northern/Bombali	Pig farm	Swine	West African Duwarf	Community close to farm bush	Semi Intensive Care	Nomadic	Freetown	Male	8 Months	Serum
109	SLE/1009/17 SW			0,21	neg	Northern/Bombali	Pig farm	Swine	West African Duwarf	Community close to farm bush	Semi Intensive Care	Nomadic	Freetown	Female	8 Months	Serum
110	SLE/1010/17 SW			0,13	neg	Northern/Bombali	Pig farm	Swine	West African Duwarf	Community close to farm bush	Semi Intensive Care	Nomadic	Freetown	Male	6 Months	Serum
111	SLE/1011/17 SW			0,15	neg	Northern/Bombali	Pig farm	Swine	West African Duwarf	Community close to farm bush	Semi Intensive Care	Nomadic	Freetown	Female	8 Months	Serum
112	SLE/1012/17 SW			0,25	neg	Northern/Bombali	Pig farm	Swine	West African Duwarf	Community close to farm bush	Semi Intensive Care	Nomadic	Freetown	Female	8 Months	Serum
113	SLE/1013/17 SW	Pool 123	neg	0,15	neg	Northern/Bombali	Pig farm	Swine	West African Duwarf	Community close to farm bush	Semi Intensive Care	Nomadic	Freetown	Male	6 Months +	Serum
114	SLE/1014/17 SW			0,13	neg	Northern/Bombali	Pig farm	Swine	West African Duwarf	Community close to farm bush	Semi Intensive Care	Nomadic	Freetown	Female	3yrs	Serum
115	SLE/1015/17 SW			0,20	neg	Northern/Bombali	Pig farm	Swine	West African Duwarf	Community close to farm bush	Semi Intensive Care	Nomadic	Freetown	Female	2 yrs +	Serum
116	SLE/1016/17 SW			0,19	neg	Northern/Bombali	Pig farm	Swine	West African Duwarf	Community close to farm bush	Semi Intensive Care	Nomadic	Freetown	Female	2 yrs+	Serum
117	SLE/1017/17 SW			0,17	neg	Northern/Bombali	Pig farm	Swine	Large white	Community close to farm bush	Semi Intensive Care	Nomadic	Freetown	Female	1 yr +	Serum
118	SLE/1018/17 SW	Pool 124	neg	0,19	neg	Northern/Bombali	Pig farm	Swine	West African Duwarf	Community close to farm bush	Semi Intensive Care	Nomadic	Freetown	Male	8 Months	Serum
119	SLE/1019/17 SW			0,12	neg	Northern/Bombali	Pig farm	Swine	West African Duwarf	Community close to farm bush	Semi Intensive Care	Nomadic	Freetown	Male	8 Months	Serum
120	SLE/1020/17 SW			0,16	neg	Northern/Bombali	Pig farm	Swine	West African Duwarf	Community close to farm bush	Semi Intensive Care	Nomadic	Freetown	Female	1 yr +	Serum
121	SLE/1021/17 SW			0,11	neg	Northern/Bombali	Pig farm	Swine	Large white	Community close to farm bush	Semi Intensive Care	Nomadic	Freetown	Male	8 Months	Serum
122	SLE/1022/17 SW			0,12	neg	Northern/Bombali	Pig farm	Swine	West African Duwarf	Community close to farm bush	Semi Intensive Care	Nomadic	Freetown	Male	8 Months	Serum
123	SLE/1023/17 SW	Pool 125	neg	0,16	neg	Northern/Bombali	Pig farm	Swine	West African Duwarf	Community close to farm bush	Semi Intensive Care	Nomadic	Freetown	Female	1 yr +	Serum
124	SLE/1024/17 SW			0,13	neg	Northern/Bombali	Pig farm	Swine	West African Duwarf	Community close to farm bush	Semi Intensive Care	Nomadic	Freetown	Male	8 Months	Serum
125	SLE/1025/17 SW			0,14	neg	Northern/Bombali	Pig farm	Swine	West African Duwarf	Community close to farm bush	Semi Intensive Care	Nomadic	Freetown	Female	3 yrs +	Serum
126	SLE/1026/17 SW			0,15	neg	Northern/Bombali	Pig farm	Swine	West African Duwarf	Community close to farm bush	Semi Intensive Care	Nomadic	Freetown	Male	2 yrs+	Serum
127	SLE/1027/17 SW			0,11	neg	Northern/Bombali	Pig farm	Swine	West African Duwarf	Community close to farm bush	Semi Intensive Care	Nomadic	Freetown	Female	1 yr +	Serum
128	SLE/1028/17 SW	Pool 126	neg	0,19	neg	Northern/Bombali	Pig farm	Swine	West African Duwarf	Community close to farm bush	Semi Intensive Care	Nomadic	Freetown	Male	10 Months	Serum
129	SLE/1029/17 SW			0,11	neg	Northern/Bombali	Pig farm	Swine	Large white	Community close to farm bush	Semi Intensive Care	Nomadic	Freetown	Male	10 Months	Serum
130	SLE/1030/17 SW			0,13	neg	Northern/Bombali	Pig farm	Swine	West African Duwarf	Community close to farm bush	Semi Intensive Care	Nomadic	Freetown	Female	2 yrs +	Serum
131	SLE/1031/17 SW			0,15	neg	Northern/Bombali	Pig farm	Swine	West African Duwarf	Community close to farm bush	Semi Intensive Care	Nomadic	Freetown	Female	8 Months	Serum
132	SLE/1032/17 SW			0,16	neg	Northern/Bombali	Pig farm	Swine	West African Duwarf	Community close to farm bush	Semi Intensive Care	Nomadic	Freetown	Male	5 yrs	Serum
133	SLE/1033/17 SW	Pool 127	neg	0,11	neg	Northern/Bombali	Pig farm	Swine	West African Duwarf	Community close to farm bush	Semi Intensive Care	Nomadic	Freetown	Male	8 Months	Serum
134	SLE/1034/17 SW			0,10	neg	Northern/Bombali	Pig farm	Swine	West African Duwarf	Community close to farm bush	Semi Intensive Care	Nomadic	Freetown	Male	8 Months	Serum
135	SLE/1035/17 SW			0,11	neg	Northern/Bombali	Pig farm	Swine	West African Duwarf	Community close to farm bush	Semi Intensive Care	Nomadic	Freetown	Male	8 Months	Serum
136	SLE/1036/17 SW			0,12	neg	Northern/Bombali	Pig farm	Swine	West African Duwarf	Community close to farm bush	Semi Intensive Care	Nomadic	Freetown	Male	7 Months	Serum
137	SLE/1037/17 SW			0,09	neg	Northern/Bombali	Pig farm	Swine	West African Duwarf	Community close to farm bush	Semi Intensive Care	Nomadic	Freetown	Male	7 Months	Serum
138	SLE/1038/17 SW	Pool 128	neg	0,19	neg	Northern/Bombali	Pig farm	Swine	West African Duwarf	Community close to farm bush	Semi Intensive Care	Nomadic	Teko Village	Female	7 Months	Serum
139	SLE/1039/17 SW			0,16	neg	Northern/Bombali	Pig farm	Swine	West African Duwarf	Community close to farm bush	Semi Intensive Care	Nomadic	Teko Village	Female	7 Months	Serum
140	SLE/1040/17 SW			0,10	neg	Northern/Bombali	Pig farm	Swine	West African Duwarf	Community close to farm bush	Semi Intensive Care	Nomadic	Teko Village	Male	7 Months	Serum
141	SLE/1041/17 SW			0,09	neg	Northern/Bombali	Pig farm	Swine	West African Duwarf	Community close to farm bush	Semi Intensive Care	Nomadic	Teko Village	Male	7 Months	Serum
142	SLE/1042/17 SW			0,12	neg	Northern/Bombali	Pig farm	Swine	West African Duwarf	Community close to farm bush	Semi Intensive Care	Nomadic	Teko Village	Male	7 Months	Serum
143	SLE/1043/17 SW	Pool 129	neg	0,10	neg	Northern/Bombali	Pig farm	Swine	West African Duwarf	Community close to farm bush	Semi Intensive Care	Nomadic	Teko Village	Male	7 Months	Serum
144	SLE/1044/17 SW			0,14	neg	Northern/Bombali	Pig farm	Swine	West African Duwarf	Community close to farm bush	Semi Intensive Care	Nomadic	Teko Village	Female	9 months	Serum
145	SLE/1045/17 SW			0,09	neg	Northern/Bombali	Pig farm	Swine	West African Duwarf	Community close to farm bush	Semi Intensive Care	Nomadic	Teko Village	Male	2 yrs	Serum
146	SLE/1046/17 SW			0,16	neg	Northern/Bombali	Pig farm	Swine	West African Duwarf	Community close to farm bush	Semi Intensive Care	Nomadic	Teko Village	Female	3 yrs	Serum
147	SLE/1047/17 SW			0,16	neg	Northern/Bombali	Pig farm	Swine	Duroc Largewhite	Community close to farm bush	Semi Intensive Care	Nomadic	Teko Village	Female	2 yrs	Serum
148	SLE/1048/17 SW	Pool 130	neg	0,11	neg	Northern/Bombali	Pig farm	Swine	Duroc Largewhite	Community close to farm bush	Semi Intensive Care	Nomadic	Teko Village	Female	10 Months	Serum
149	SLE/1049/17 SW			0,13	neg	Northern/Bombali	Pig farm	Swine	Duroc Largewhite	Community close to farm bush	Semi Intensive Care	Nomadic	Teko Village	Male	2 yrs	Serum
150	SLE/1050/17 SW			0,09	neg	Northern/Bombali	Pig farm	Swine	Duroc Largewhite	Community close to farm bush	Semi Intensive Care	Nomadic	Teko Village	Female	1 yr	Serum
151	SLE/1051/17 SW			0,32	pos	Northern/Bombali	Pig farm	Swine	West African Duwarf	Community close to farm bush	Semi Intensive Care	Nomadic	Makeni Town	Male	1 yr +	Serum
152	SLE/1052/17 SW			0,15	neg	Northern/Bombali	Pig farm	Swine	West African Duwarf	Community close to farm bush	Semi Intensive Care	Nomadic	Makeni Town	Female	1 yr +	Serum
153	SLE/1053/17 SW	Pool 131	neg	0,11	neg	Northern/Bombali	Pig farm	Swine	West African Duwarf	Community close to farm bush	Semi Intensive Care	Nomadic	Makeni Town	Female	8 Months	Serum
154	SLE/1054/17 SW			0,15	neg	Northern/Bombali	Pig farm	Swine	West African Duwarf	Community close to farm bush	Semi Intensive Care	Nomadic	Makeni Town	Female	1 yr +	Serum
155	SLE/1055/17 SW			0,12	neg	Northern/Bombali	Pig farm	Swine	West African Duwarf	Community close to farm bush	Semi Intensive Care	Nomadic	Makeni Town	Female	8 Months	Serum
156	SLE/1056/17 SW			0,13	neg	Northern/Bombali	Pig farm	Swine	West African Duwarf	Community close to farm bush	Semi Intensive Care	Nomadic	Makeni Town	Male	8 Months	Serum
157	SLE/1057/17 SW			0,12	neg	Northern/Bombali	Pig farm	Swine	West African Duwarf	Community close to farm bush	Semi Intensive Care	Nomadic	Makeni Town	Male	3 yrs +	Serum
158	SLE/1058/17 SW	Pool 132	neg	0,14	neg	Northern/Bombali	Pig farm	Swine	West African Duwarf	Community close to farm bush	Semi Intensive Care	Nomadic	Makeni Town	Female	2 yrs	Serum
159	SLE/1059/17 SW			0,10	neg	Northern/Bombali	Pig farm	Swine	West African Duwarf	Community close to farm bush	Semi Intensive Care	Nomadic	Makeni Town	Female	Gilt	Serum
160	SLE/1060/17 SW			0,15	neg	Northern/Bombali	Pig farm	Swine	West African Duwarf	Community close to farm bush	Semi Intensive Care	Nomadic	Makeni Town	Female	Gilt	Serum
161	SLE/1061/17 SW			0,10	neg	Northern/Bombali	Pig farm	Swine	West African Duwarf	Community close to farm bush	Semi Intensive Care	Nomadic	Makeni Town	Female	Gilt	Serum
162	SLE/1062/17 SW			0,13	neg	Northern/Bombali	Pig farm	Swine	West African Duwarf	Community close to farm bush	Free range	Nomadic	Makeni Town	Female	Gilt	Serum
163	SLE/1063/17 SW	Pool 133	neg	0,14	neg	Northern/Bombali	Pig farm	Swine	West African Duwarf	Community close to farm bush	Free range	Nomadic	Makeni Town	Female	Gilt	Serum
164	SLE/1064/17 SW			0,11	neg	Northern/Bombali	Pig farm	Swine	West African Duwarf	Community close to farm bush	Free range	Nomadic	Makeni Town	Male	Grower	Serum
165	SLE/1065/17 SW			0,14	neg	Northern/Bombali	Pig farm	Swine	West African Duwarf	Community close to farm bush	Free range	Nomadic	Makeni Town	Male	Grower	Serum
166	SLE/1066/17 SW			0,15	neg	Northern/Bombali	Pig farm	Swine	West African Duwarf	Community close to farm bush	Free range	Nomadic	Makeni Town	Male	Grower	Serum
167	SLE/1067/17 SW			0,09	neg	Northern/Bombali	Pig farm	Swine	West African Duwarf	Community close to farm bush	Free range	Nomadic	Makeni Town	Female	Adult	Serum
168	SLE/1068/17 SW	Pool 134	neg	0,17	neg	Northern/Bombali	Pig farm	Swine	West African Duwarf	Community close to farm bush	Free range	Nomadic	Makeni Town	Female	Adult	Serum
169	SLE/1069/17 SW			0,11	neg	Northern/Bombali	Pig farm	Swine	West African Duwarf	Community close to farm bush	Free range	Nomadic	Makeni Town	Female	Gilt	Serum
170	SLE/1070/17 SW			0,15	neg	Northern/Bombali	Pig farm	Swine	West African Duwarf	Community close to farm bush	Free range	Nomadic	Makeni Town	Female	Gilt	Serum
171	SLE/1071/17 SW			0,13	neg	Northern/Bombali	Pig farm	Swine	West African Duwarf	Community close to farm bush	Free range	Nomadic	Congo Town	Female	Gilt	Serum
172	SLE/1072/17 SW			0,14	neg	Northern/Bombali	Pig farm	Swine	West African Duwarf	Community close to farm bush	Free range	Nomadic	Congo Town	Male	Grower	Serum
173	SLE/1073/17 SW	Pool 135	neg	0,11	neg	Northern/Bombali	Pig farm	Swine	West African Duwarf	Community close to farm bush	Free range	Nomadic	Congo Town	Male	Grower	Serum

174	SLE/1074/17 SW			0,09	neg	Northern/Bombali	Pig farm	Swine	West African Duwarf	Community close to farm bush	Free range	Nomadic	Congo Town	Male	Grower	Serum
175	SLE/1075/17 SW			0,12	neg	Northern/Bombali	Pig farm	Swine	West African Duwarf	Community close to farm bush	Free range	Nomadic	Congo Town	Male	Grower	Serum
176	SLE/1076/17 SW			0,08	neg	Northern/Bombali	Pig farm	Swine	West African Duwarf	Community close to farm bush	Free range	Nomadic	Congo Town	Male	Grower	Serum
177	SLE/1077/17 SW			0,10	neg	Northern/Bombali	Pig farm	Swine	West African Duwarf	Community close to farm bush	Free range	Nomadic	Congo Town	Male	Grower	Serum
178	SLE/1078/17 SW	Pool 136	neg	0,15	neg	Northern/Bombali	Pig farm	Swine	West African Duwarf	Community close to farm bush	Semi Intensive Care	Nomadic	Congo Town	Male	Grower	Serum
179	SLE/1079/17 SW			0,11	neg	Northern/Bombali	Pig farm	Swine	West African Duwarf	Community close to farm bush	Semi Intensive Care	Nomadic	Congo Town	Male	Grower	Serum
180	SLE/1080/17 SW			0,12	neg	Northern/Bombali	Pig farm	Swine	West African Duwarf	Community close to farm bush	Semi Intensive Care	Nomadic	Congo Town	Male	Grower	Serum
181	SLE/1081/17 SW			0,17	neg	Northern/Bombali	Pig farm	Swine	West African Duwarf	Community close to farm bush	Semi Intensive Care	Nomadic	Congo Town	Male	Grower	Serum
182	SLE/1082/17 SW			0,12	neg	Northern/Bombali	Pig farm	Swine	West African Duwarf	Community close to farm bush	Semi Intensive Care	Nomadic	Congo Town	Female	Adult	Serum
183	SLE/1083/17 SW	Pool 137	neg	0,14	neg	Northern/Bombali	Pig farm	Swine	West African Duwarf	Community close to farm bush	Semi Intensive Care	Nomadic	Congo Town	Male	Boar	Serum
184	SLE/1084/17 SW			0,14	neg	Northern/Bombali	Pig farm	Swine	West African Duwarf	Community close to farm bush	Semi Intensive Care	Nomadic	Congo Town	Male	Boar	Serum
185	SLE/1085/17 SW			0,27	neg	Northern/Bombali	Pig farm	Swine	West African Duwarf	Community close to farm bush	Semi Intensive Care	Nomadic	Teko village	Male	Grower	Serum
186	SLE/1086/17 SW			0,11	neg	Northern/Bombali	Pig farm	Swine	West African Duwarf	Community close to farm bush	Semi Intensive Care	Nomadic	Teko village	Male	Grower	Serum
187	SLE/1087/17 SW			0,13	neg	Northern/Bombali	Pig farm	Swine	West African Duwarf	Community close to farm bush	Semi Intensive Care	Nomadic	Teko village	Male	Grower	Serum
188	SLE/1088/17 SW	Pool 138	neg	0,13	neg	Northern/Bombali	Pig farm	Swine	West African Duwarf	Community close to farm bush	Semi Intensive Care	Nomadic	Teko village	Male	Grower	Serum
189	SLE/1089/17 SW			0,11	neg	Northern/Bombali	Pig farm	Swine	West African Duwarf	Community close to farm bush	Semi Intensive Care	Nomadic	Teko village	Male	Grower	Serum
190	SLE/1090/17 SW			0,10	neg	Northern/Bombali	Pig farm	Swine	West African Duwarf	Community close to farm bush	Semi Intensive Care	Nomadic	Teko village	Female	Adult	Serum
191	SLE/1091/17 SW			0,12	neg	Northern/Bombali	Pig farm	Swine	West African Duwarf	Community close to farm bush	Semi Intensive Care	Nomadic	Teko village	Female	Adult	Serum
192	SLE/1092/17 SW			0,11	neg	Northern/Bombali	Pig farm	Swine	West African Duwarf	Community close to farm bush	Semi Intensive Care	Nomadic	Teko village	Female	Gilt	Serum
193	SLE/1093/17 SW	Pool 139	neg	0,15	neg	Northern/Bombali	Pig farm	Swine	West African Duwarf	Community close to farm bush	Semi Intensive Care	Nomadic	Teko village	Female	Gilt	Serum
194	SLE/1094/17 SW			0,15	neg	Northern/Bombali	Pig farm	Swine	West African Duwarf	Community close to farm bush	Semi Intensive Care	Nomadic	Teko village	Female	Gilt	Serum
195	SLE/1095/17 SW			0,11	neg	Northern/Bombali	Pig farm	Swine	West African Duwarf	Community close to farm bush	Semi Intensive Care	Nomadic	Teko village	Female	Gilt	Serum
196	SLE/1096/17 SW			0,12	neg	n/a	n/a	n/a	n/a	n/a	n/a	Nomadic	Teko village	Female	Gilt	Serum
197	SLE/1097/17 SW			0,09	neg	n/a	n/a	n/a	n/a	n/a	n/a	Nomadic	Teko village	Female	Gilt	Serum
198	SLE/1098/17 SW	Pool 140	neg	0,14	neg	n/a	n/a	n/a	n/a	n/a	n/a	Nomadic	Teko village	Female	Gilt	Serum
199	SLE/1099/17 SW			0,08	neg	n/a	n/a	n/a	n/a	n/a	n/a	Nomadic	Teko village	Female	Gilt	Serum
200	SLE/1100/17 SW			0,10	neg	n/a	n/a	n/a	n/a	n/a	n/a	Nomadic	Teko village	Female	Gilt	Serum

Nr	Serum pools (Nr)	Findings (PCR)	OD405	Finding	*Region*	District	*Sampling Unit/Town/Village*	*Animal Species*	*Breed*	*Habitat*	*Housing/stable/Pasture/ Stationary/nomadic*	*Herd/Flock/Farm*	*Date of sample collection*	*History of migration*	*Gender*	*Age in month*
SL 2017-401			0,23	neg	South	BO	Tikonko Town	Swine	Large white	Farm bush	Stable	Herd	26.05.2017	Sunga	F	18
SL 2017-402			0,28	neg	South	BO	Tikonko Town	Swine	Large white	Farm bush	Stable	Herd	26.05.2017	Sunga	F	18
SL 2017-403	Pool 1	neg	0,21	neg	South	BO	Tikonko Town	Swine	Large white	Farm bush	Stable	Herd	26.05.2017	Sunga	F	24
SL 2017-404			0,30	neg	South	BO	Tikonko Town	Swine	Large white	Farm bush	Stable	Herd	26.05.2017	Sunga	F	24
SL 2017-405			0,15	neg	South	BO	Tikonko Town	Swine	Large white	Farm bush	Stable	Herd	26.05.2017	Sunga	F	6
SL 2017-406			0,19	neg	South	BO	Tikonko Town	Swine	Large white	Farm bush	Stable	Herd	26.05.2017	Sunga	F	6
SL 2017-407			0,20	neg	South	BO	Tikonko Town	Swine	Large white	Farm bush	Stable	Herd	26.05.2017	Sunga	F	24
SL 2017-408	Pool 2	neg	0,22	neg	South	BO	Tikonko Town	Swine	Large white	Farm bush	Stable	Herd	26.05.2017	Sunga	F	24
SL 2017-409			0,28	neg	South	BO	Tikonko Town	Swine	Large white	Farm bush	Stable	Herd	26.05.2017	Sunga	F	18
SL 2017-410			0,25	neg	South	BO	Tikonko Town	Swine	Large white	Farm bush	Stable	Herd	26.05.2017	Sunga	F	36
SL 2017-411			0,26	neg	South	BO	Tikonko Town	Swine	Large white	Farm bush	Stable	Herd	26.05.2017	Sunga	F	24
SL 2017-412			0,17	neg	South	BO	Tikonko Town	Swine	Large white	Farm bush	Stable	Herd	26.05.2017	Sunga	M	12
SL 2017-413	Pool 3	neg	0,16	neg	South	BO	Tikonko Town	Swine	Large white	Farm bush	Stable	Herd	26.05.2017	Sunga	F	12
SL 2017-414			0,20	neg	South	BO	Tikonko Town	Swine	Large white	Farm bush	Stable	Herd	26.05.2017	Sunga	F	14
SL 2017-415			0,19	neg	South	BO	Tikonko Town	Swine	Large white	Farm bush	Stable	Herd	26.05.2017	Sunga	F	6
SL 2017-416			0,16	neg	South	BO	Tikonko Town	Swine	Large white	Farm bush	Stable	Herd	26.05.2017	Sunga	F	6
SL 2017-417			0,20	neg	South	BO	Tikonko Town	Swine	Large white	Farm bush	Stable	Herd	26.05.2017	Sunga	F	6
SL 2017-418	Pool 4	neg	0,33	neg	South	BO	Tikonko Town	Swine	Large white	Farm bush	Stable	Herd	26.05.2017	Bo city	F	6
SL 2017-419			0,12	neg	South	BO	Tikonko Town	Swine	Large white	Farm bush	Stable	Herd	26.05.2017	Bo city	F	8
SL 2017-420			0,19	neg	South	BO	Tikonko Town	Swine	Large white	Farm bush	Stable	Herd	26.05.2017	Bo city	F	8
SL 2017-421			0,16	neg	South	BO	Tikonko Town	Swine	Large white	Farm bush	Stable	Herd	26.05.2017	Bo city	F	8
SL 2017-422			0,21	neg	South	BO	Tikonko Town	Swine	Large white	Farm bush	Stable	Herd	26.05.2017	Bo city	F	8
SL 2017-423	Pool 5	neg	0,24	neg	South	BO	Tikonko Town	Swine	Large white	Farm bush	Stable	Herd	26.05.2017	Bo city	F	8
SL 2017-424			0,15	neg	South	BO	Tikonko Town	Swine	Large white	Farm bush	Stable	Herd	26.05.2017	Bo city	M	6
SL 2017-425			0,17	neg	South	BO	Tikonko Town	Swine	Large white	Farm bush	Stable	Herd	26.05.2017	Bo city	M	6
SL 2017-426			0,21	neg	South	BO	Tikonko Town	Swine	Large white	Farm bush	Stable	Herd	26.05.2017	Bo city	M	6
SL 2017-427			------	neg	South	BO	Tikonko Town	Swine	Large white	Farm bush	Stable	Herd	26.05.2017	Bo city	M	6
SL 2017-428	Pool 6	neg	0,21	neg	South	BO	Tikonko Town	Swine	Large white	Farm bush	Stable	Herd	26.05.2017	Bo city	F	18
SL 2017-429			0,25	neg	South	BO	Tikonko Town	Swine	Large white	Farm bush	Stable	Herd	26.05.2017	Bo city	F	24
SL 2017-430			0,25	neg	South	BO	Tikonko Town	Swine	Large white	Farm bush	Stable	Herd	26.05.2017	Bo city	F	14
SL 2017-431			0,15	neg	South	BO	Lembema	Swine	Large white	Forest	Stable	Flock	27.05.2017	Bo city	F	3
SL 2017-432			0,16	neg	South	BO	Lembema	Swine	Large white	Forest	Stable	Flock	27.05.2017	Bo city	F	3
SL 2017-433	Pool 7	neg	0,19	neg	South	BO	Lembema	Swine	Large white	Forest	Stable	Flock	27.05.2017	Bo city	F	3
SL 2017-434			0,18	neg	South	BO	Lembema	Swine	Large white	Forest	Stable	Flock	27.05.2017	Bo city	F	3
SL 2017-435			0,17	neg	South	BO	Lembema	Swine	Large white	Forest	Stable	Flock	27.05.2017	Bo city	F	3
SL 2017-436			0,27	neg	South	BO	Lembema	Swine	Large white	Forest	Stable	Flock	27.05.2017	Bo city	F	18
SL 2017-437			0,18	neg	South	BO	Lembema	Swine	Large white	Forest	Stable	Flock	27.05.2017	Bo city	F	3
SL 2017-438	Pool 8	neg	0,17	neg	South	BO	Lembema	Swine	Large white	Forest	Stable	Flock	27.05.2017	Bo city	F	18
SL 2017-439			0,17	neg	South	BO	Lembema	Swine	Large white	Forest	Stable	Flock	27.05.2017	Bo city	F	18
SL 2017-440			0,24	neg	South	BO	Lembema	Swine	Large white	Forest	Stable	Flock	27.05.2017	Bo city	F	16
SL 2017-441			0,21	neg	South	BO	Lembema	Swine	West Africa Duroc	Forest	Stable	Flock	27.05.2017	Bo city	M	6
SL 2017-442			0,17	neg	South	BO	Lembema	Swine	West Africa Duroc	Forest	Stable	Flock	27.05.2017	Bo city	M	12
SL 2017-443	Pool 9	neg	0,28	neg	South	BO	Lembema	Swine	West Africa Duroc	Forest	Stable	Flock	27.05.2017	Bo city	F	18
SL 2017-444			0,15	neg	South	BO	Lembema	Swine	West Africa Duroc	Forest	Stable	Flock	27.05.2017	Bo city	F	3
SL 2017-445			0,25	neg	South	BO	Lembema One	Swine	West Africa Duroc	Forest	Stable	Flock	27.05.2017	Bo city	F	18
SL 2017-446			0,17	neg	South	BO	Lembema One	Swine	West Africa Duroc	Forest	Stable	Flock	27.05.2017	Bo city	F	6
SL 2017-447			0,14	neg	South	BO	Lembema One	Swine	West Africa Duroc	Forest	Stable	Flock	27.05.2017	Bo city	M	6
SL 2017-448	Pool 10	neg	0,17	neg	South	BO	Lembema One	Swine	West Africa Duroc	Forest	Stable	Flock	27.05.2017	Bo city	M	6
SL 2017-449			0,19	neg	South	BO	Lembema One	Swine	West Africa Duroc	Farm bush	Stable	Herd	28.05.2017	Bo city	F	18
SL 2017-450			0,14	neg	South	BO	Lembema One	Swine	West Africa Duroc	Farm bush	Stable	Herd	28.05.2017	Bo city	F	3
SL 2017-451			0,13	neg	South	BO	Lembema One	Swine	West Africa Duroc	Farm bush	Stable	Herd	28.05.2017	Bo city	M	3
SL 2017-452			0,14	neg	South	BO	Lembema One	Swine	West Africa Duroc	Farm bush	Stable	Herd	28.05.2017	Bo city	M	3
SL 2017-453	Pool 11	neg	0,14	neg	South	BO	Lembema One	Swine	West Africa Duroc	Farm bush	Stable	Herd	28.05.2017	Bo city	M	3
SL 2017-454			----------	neg	South	BO	Nyandehun	Swine	Duroc	Farm bush	Stable	Herd	30.05.2017	Bo city	F	18
SL 2017-455			0,19	neg	South	BO	Nyandehun	Swine	Duroc	Farm bush	Stable	Herd	30.05.2017	Bo city	F	28
SL 2017-456			0,14	neg	South	BO	Nyandehun	Swine	Duroc	Farm bush	Stable	Herd	30.05.2017	Bo city	M	4
SL 2017-457			0,12	neg	South	BO	Nyandehun	Swine	Duroc	Farm bush	Stable	Herd	30.05.2017	Bo city	F	4
SL 2017-458	Pool 12	neg	0,14	neg	South	BO	Nyandehun	Swine	Duroc	Farm bush	Stable	Herd	30.05.2017	Bo city	F	18
SL 2017-459			0,21	neg	South	BO	Nyandehun	Swine	Duroc	Farm bush	Stable	Herd	30.05.2017	Bo city	F	20
SL 2017-460			0,21	neg	South	BO	Nyandehun	Swine	Large white	Farm bush	Stable	Herd	30.05.2017	Bo city	M	22

SL 2017-461			0,15	neg	*South*	*BO*	*Nyandehun*	*Swine*	*Large white*	*Farm bush*	*Stable*	*Herd*	*30.05.2017*	*Bo city*	*M*	*8*
SL 2017-462			0,19	neg	*South*	*BO*	*Nyandehun*	*Swine*	*Large white*	*Farm bush*	*Stable*	*Herd*	*30.05.2017*	*Bo city*	*F*	*8*
SL 2017-463	Pool 13	neg	0,17	neg	*South*	*BO*	*Nyandehun*	*Swine*	*Large white*	*Farm bush*	*Stable*	*Herd*	*30.05.2017*	*Bo city*	*M*	*8*
SL 2017-464			0,18	neg	*South*	*BO*	*Nyandehun*	*Swine*	*Large white*	*Farm bush*	*Stable*	*Herd*	*30.05.2017*	*Bo city*	*F*	*18*
SL 2017-465			0,20	neg	*South*	*BO*	*Nyandehun*	*Swine*	*Large white*	*Farm bush*	*Stable*	*Herd*	*30.05.2017*	*Bo city*	*F*	*18*
SL 2017-466			0,18	neg	*South*	*BO*	*Nyandehun*	*Swine*	*Large white*	*Farm bush*	*Stable*	*Herd*	*30.05.2017*	*Bo city*	*F*	*14*
SL 2017-467			0,29	neg	*South*	*BO*	*Bo Town*	*Swine*	*Large white*	*Farm bush*	*Stable*	*Farm*	*30.05.2017*	*Bo city*	*F*	*10*
SL 2017-468	Pool 14	neg	0,18	neg	*South*	*BO*	*Bo Town*	*Swine*	*Large white*	*Farm bush*	*Stable*	*Farm*	*30.05.2017*	*Bo city*	*F*	*10*
SL 2017-469			0,18	neg	*South*	*BO*	*Bo Town*	*Swine*	*Large white*	*Farm bush*	*Stable*	*Farm*	*30.05.2017*	*Bo city*	*F*	*10*
SL 2017-470			0,16	neg	*South*	*BO*	*Bo Town*	*Swine*	*Large white*	*Farm bush*	*Stable*	*Farm*	*30.05.2017*	*Bo city*	*F*	*10*
SL 2017-471			0,15	neg	*South*	*BO*	*Bo Town*	*Swine*	*Large white*	*Farm bush*	*Stable*	*Farm*	*30.05.2017*	*Bo city*	*F*	*9*
SL 2017-472			0,14	neg	*South*	*BO*	*Bo Town*	*Swine*	*Duroc*	*Farm bush*	*Stable*	*Farm*	*30.05.2017*	*Bo city*	*F*	*9*
SL 2017-473	Pool 15	neg	0,17	neg	*South*	*BO*	*Bo Town*	*Swine*	*Duroc*	*Farm bush*	*Stable*	*Farm*	*30.05.2017*	*Bo city*	*F*	*9*
SL 2017-474			0,20	neg	*South*	*BO*	*Bo Town*	*Swine*	*Duroc*	*Farm bush*	*Stable*	*Farm*	*30.05.2017*	*Bo city*	*M*	*8*
SL 2017-475			0,16	neg	*South*	*BO*	*Bo Town*	*Swine*	*Duroc*	*Farm bush*	*Stable*	*Farm*	*30.05.2017*	*Bo city*	*M*	*10*
SL 2017-476			0,33	neg	*South*	*BO*	*Bo Town*	*Swine*	*Large white*	*Farm bush*	*Stable*	*Farm*	*30.05.2017*	*Bo city*	*M*	*10*
SL 2017-477			0,18	neg	*South*	*BO*	*Bo Town*	*Swine*	*Duroc*	*Farm bush*	*Stable*	*Farm*	*30.05.2017*	*Bo city*	*M*	*10*
SL 2017-478	Pool 16	neg	0,28	neg	*South*	*BO*	*Bo Town*	*Swine*	*Large white*	*Farm bush*	*Stable*	*Farm*	*30.05.2017*	*Bo city*	*M*	*18*
SL 2017-479			0,22	neg	*South*	*BO*	*Bo Town*	*Swine*	*Large white*	*Farm bush*	*Stable*	*Farm*	*30.05.2017*	*Bo city*	*M*	*6*
SL 2017-480			0,15	neg	*South*	*BO*	*Bo Town*	*Swine*	*Large white*	*Farm bush*	*Stable*	*Farm*	*30.05.2017*	*Bo city*	*M*	*10*
SL 2017-481			0,21	neg	*South*	*BO*	*Bo Town*	*Swine*	*Large white*	*Farm bush*	*Stable*	*Herd*	*31.05.2017*	*Freetown*	*F*	*14*
SL 2017-482			0,17	neg	*South*	*BO*	*Bo Town*	*Swine*	*Large white*	*Farm bush*	*Stable*	*Herd*	*31.05.2017*	*Freetown*	*M*	*3*
SL 2017-483	Pool 17	neg	0,15	neg	*South*	*BO*	*Bo Town*	*Swine*	*Duroc*	*Farm bush*	*Stable*	*Herd*	*31.05.2017*	*Freetown*	*F*	*3*
SL 2017-484			0,14	neg	*South*	*BO*	*Bo Town*	*Swine*	*Duroc*	*Farm bush*	*Stable*	*Herd*	*31.05.2017*	*Freetown*	*M*	*3*
SL 2017-485			0,13	neg	*South*	*BO*	*Bo Town*	*Swine*	*Duroc*	*Farm bush*	*Stable*	*Herd*	*31.05.2017*	*Freetown*	*M*	*3*
SL 2017-486			0,14	neg	*South*	*BO*	*Bo Town*	*Swine*	*Duroc*	*Farm bush*	*Stable*	*Herd*	*31.05.2017*	*Freetown*	*F*	*3*
SL 2017-487			0,15	neg	*South*	*BO*	*Bo Town*	*Swine*	*Duroc*	*Farm bush*	*Stable*	*Herd*	*31.05.2017*	*Freetown*	*F*	*3*
SL 2017-488	Pool 18	neg	0,17	neg	*South*	*BO*	*Bo Town*	*Swine*	*Large white*	*Farm bush*	*Stable*	*Herd*	*31.05.2017*	*Freetown*	*M*	*3*
SL 2017-489			0,16	neg	*South*	*BO*	*Bo Town*	*Swine*	*Large white*	*Farm bush*	*Stable*	*Herd*	*31.05.2017*	*Freetown*	*F*	*20*
SL 2017-490			0,14	neg	*South*	*BO*	*Bo Town*	*Swine*	*Large white*	*Farm bush*	*Stable*	*Herd*	*31.05.2017*	*Freetown*	*F*	*2*
SL 2017-491			0,23	neg	*South*	*BO*	*Bo Town*	*Swine*	*Large white*	*Farm bush*	*Stable*	*Herd*	*31.05.2017*	*Freetown*	*M*	*2*
SL 2017-492			0,17	neg	*South*	*BO*	*Bo Town*	*Swine*	*Large white*	*Farm bush*	*Stable*	*Herd*	*31.05.2017*	*Freetown*	*M*	*2*
SL 2017-493	Pool 19	neg	0,14	neg	*South*	*BO*	*Bo Town*	*Swine*	*Large white*	*Farm bush*	*Stable*	*Herd*	*31.05.2017*	*Freetown*	*F*	*18*
SL 2017-494			0,18	neg	*South*	*BO*	*Bo Town*	*Swine*	*Duroc*	*Farm bush*	*Stable*	*Herd*	*31.05.2017*	*Freetown*	*F*	*3*
SL 2017-495			0,14	neg	*South*	*BO*	*Bo Town*	*Swine*	*Large white*	*Farm bush*	*Stable*	*Herd*	*31.05.2017*	*Freetown*	*M*	*3*
SL 2017-496			0,14	neg	*South*	*BO*	*Bo Town*	*Swine*	*Large white*	*Farm bush*	*Stable*	*Herd*	*31.05.2017*	*Freetown*	*F*	*3*
SL 2017-497			0,14	neg	*South*	*BO*	*Bo Town*	*Swine*	*Large white*	*Farm bush*	*Stable*	*Herd*	*31.05.2017*	*Freetown*	*F*	*3*
SL 2017-498	Pool 20	neg	0,24	neg	*South*	*BO*	*Bo Town*	*Swine*	*Large white*	*Farm bush*	*Stable*	*Herd*	*31.05.2017*	Holland	F	*8*
SL 2017-499			0,17	neg	*South*	*BO*	*Bo Town*	*Swine*	*Large white*	*Farm bush*	*Stable*	*Herd*	*31.05.2017*	Holland	M	*8*
SL 2017-500			0,18	neg	*South*	*BO*	*Bo Town*	*Swine*	*Large white*	*Farm bush*	*Stable*	*Herd*	*31.05.2017*	Holland	F	*8*
SL 2017-501			0,13	neg	*South*	*BO*	*Bo Town*	*Swine*	*Large white*	*Farm bush*	*Stable*	*Herd*	*31.05.2017*	Holland	M	*8*
SL 2017-502			0,15	neg	*South*	*BO*	*Bo Town*	*Swine*	Duroc	*Farm bush*	*Stable*	*Herd*	*31.05.2017*	Holland	F	*8*
SL 2017-503	Pool 21	neg	0,19	neg	*South*	*BO*	*Bo Town*	*Swine*	Duroc	*Farm bush*	*Stable*	*Herd*	*31.05.2017*	Holland	F	*8*
SL 2017-504			0,16	neg	*South*	*BO*	*Bo Town*	*Swine*	*Large white*	*Farm bush*	*Stable*	*Herd*	*01.06.2017*	n/a	F	*22*
SL 2017-505			0,20	neg	*South*	*BO*	*Bo Town*	*Swine*	*Large white*	*Farm bush*	*Stable*	*Herd*	*01.06.2017*	n/a	F	*22*
SL 2017-506			0,26	neg	*South*	*BO*	*Bo Town*	*Swine*	*Large white*	*Farm bush*	*Stable*	*Herd*	*01.06.2017*	n/a	F	*18*
SL 2017-507			0,14	neg	*South*	*BO*	*Bo Town*	*Swine*	*Duroc*	*Farm bush*	*Stable*	*Herd*	*01.06.2017*	n/a	F	*3*
SL 2017-508	Pool 22	neg	0,16	neg	*South*	*BO*	*Bo Town*	*Swine*	*Large white*	*Farm bush*	*Stable*	*Herd*	*01.06.2017*	n/a	M	*3*
SL 2017-509			0,16	neg	*South*	*BO*	*Bo Town*	*Swine*	*Large white*	*Farm bush*	*Stable*	*Herd*	*01.06.2017*	n/a	F	*3*
SL 2017-510			0,25	neg	*South*	*BO*	*Bo Town*	*Swine*	*Large white*	*Farm bush*	*Stable*	*Herd*	*01.06.2017*	n/a	F	*20*
SL 2017-511			0,14	neg	*South*	*BO*	*Bo Town*	*Swine*	*Duroc*	*Farm bush*	*Stable*	*Herd*	*01.06.2017*	n/a	M	*3*
SL 2017-512			0,15	neg	*South*	*BO*	*Bo Town*	*Swine*	*Large white*	*Farm bush*	*Stable*	*Herd*	*01.06.2017*	n/a	M	*3*
SL 2017-513	Pool 23	neg	0,18	neg	*South*	*BO*	*Bo Town*	*Swine*	*Large white*	*Farm bush*	*Stable*	*Herd*	*01.06.2017*	n/a	M	*3*
SL 2017-514			0,18	neg	*South*	*BO*	*Bo Town*	*Swine*	*Large white*	*Farm bush*	*Stable*	*Herd*	*01.06.2017*	n/a	F	*3*
SL 2017-515			0,16	neg	*South*	*BO*	*Bo Town*	*Swine*	*Large white*	*Farm bush*	*Stable*	*Herd*	*01.06.2017*	n/a	M	*3*
SL 2017-516			0,16	neg	*South*	*BO*	*Bo Town*	*Swine*	*Large white*	*Farm bush*	*Stable*	*Herd*	*01.06.2017*	n/a	M	*3*
SL 2017-517			0,17	neg	*South*	*BO*	*Bo Town*	*Swine*	*Large white*	*Farm bush*	*Stable*	*Herd*	*01.06.2017*	n/a	M	*3*
SL 2017-518	Pool 24	neg	0,20	neg	*South*	*BO*	*Bo Town*	*Swine*	*Duroc*	*Farm bush*	*Stable*	*Herd*	*01.06.2017*	n/a	M	*24*
SL 2017-519			0,23	neg	*South*	*BO*	*Bo Town*	*Swine*	*Large white*	*Farm bush*	*Stable*	*Herd*	*01.06.2017*	n/a	M	*3*
SL 2017-520			0,14	neg	*South*	*BO*	*Bo Town*	*Swine*	*Large white*	*Farm bush*	*Stable*	*Herd*	*01.06.2017*	n/a	F	*24*
SL 2017-521			0,21	neg	*South*	*BO*	*Bo Town*	*Swine*	*Large white*	*Farm bush*	*Stable*	*Herd*	*01.06.2017*	n/a	F	*24*
SL 2017-522			0,26	neg	*South*	*BO*	*Bo Town*	*Swine*	*Large white*	*Farm bush*	*Stable*	*Herd*	*01.06.2017*	n/a	F	*24*
SL 2017-523	Pool 25	neg	0,21	neg	*South*	*BO*	*Bo Town*	*Swine*	*Large white*	*Farm bush*	*Stable*	*Herd*	*01.06.2017*	n/a	F	*24*
SL 2017-524			0,22	neg	*South*	*BO*	*Bo Town*	*Swine*	*Duroc*	*Farm bush*	*Stable*	*Herd*	*01.06.2017*	n/a	F	*24*
SL 2017-525			0,26	neg	*South*	*BO*	*Bo Town*	*Swine*	*Large white*	*Farm bush*	*Stable*	*Herd*	*01.06.2017*	n/a	F	*24*
SL 2017-526			0,14	neg	*South*	*BO*	*Bo Town*	*Swine*	*Large white*	*Farm bush*	*Stable*	*Herd*	*01.06.2017*	n/a	F	*3*
SL 2017-527			0,14	neg	*South*	*BO*	*Bo Town*	*Swine*	*Large white*	*Farm bush*	*Stable*	*Herd*	*01.06.2017*	n/a	M	*3*
SL 2017-528	Pool 26	neg	0,14	neg	*South*	*BO*	*Bo Town*	*Swine*	*Large white*	*Farm bush*	*Stable*	*Herd*	*01.06.2017*	n/a	F	*24*
SL 2017-529			0,21	neg	*South*	*BO*	*Bo Town*	*Swine*	*Large white*	*Farm bush*	*Stable*	*Herd*	*01.06.2017*	n/a	F	*3*
SL 2017-530			0,18	neg	*South*	*BO*	*Bo Town*	*Swine*	*Duroc*	*Farm bush*	*Stable*	*Herd*	*01.06.2017*	n/a	M	*3*
SL 2017-531			0,17	neg	*South*	*BO*	*Bo Town*	*Swine*	*Duroc*	*Farm bush*	*Stable*	*Herd*	*01.06.2017*	n/a	F	*3*
SL 2017-532			0,19	neg	*South*	*BO*	*Bo Town*	*Swine*	*Duroc*	*Farm bush*	*Stable*	*Herd*	*01.06.2017*	n/a	F	*3*
SL 2017-533	Pool 27	neg	0,17	neg	*South*	*BO*	*Bo Town*	*Swine*	*Large white*	*Farm bush*	*Stable*	*Herd*	*01.06.2017*	n/a	M	*3*
SL 2017-534			0,17	neg	*South*	*BO*	*Bo Town*	*Swine*	*Large white*	*Farm bush*	*Stable*	*Herd*	*01.06.2017*	n/a	M	*3*
SL 2017-535			0,16	neg	*South*	*BO*	*Bo Town*	*Swine*	*Large white*	*Farm bush*	*Stable*	*Herd*	*01.06.2017*	n/a	M	*3*
SL 2017-536			0,16	neg	*South*	*BO*	*Bo Town*	*Swine*	*Large white*	*Farm bush*	*Stable*	*Herd*	*01.06.2017*	n/a	F	*3*
SL 2017-537			0,17	neg	*South*	*BO*	*Bo Town*	*Swine*	*Large white*	*Farm bush*	*Stable*	*Herd*	*01.06.2017*	n/a	M	*3*
SL 2017-538	Pool 28	neg	0,24	neg	*South*	*BO*	*Bo Town*	*Swine*	*Large white*	*Farm bush*	*Stable*	*Herd*	*01.06.2017*	n/a	F	*3*
SL 2017-539			0,17	neg	*South*	*BO*	*Bo Town*	*Swine*	*Large white*	*Farm bush*	*Stable*	*Herd*	*01.06.2017*	n/a	F	*3*
SL 2017-540			0,18	neg	*South*	*BO*	*Bo Town*	*Swine*	*Large white*	*Farm bush*	*Stable*	*Herd*	*01.06.2017*	n/a	M	*3*
SL 2017-541			0,14	neg	*South*	*BO*	*Torwama Town*	*Swine*	*Large white*	*Farm bush*	*Stable*	*Herd*	*02.06.2017*	*Bumpeh*	M	*8*
SL 2017-542			0,17	neg	*South*	*BO*	*Torwama Town*	*Swine*	*Large white*	*Farm bush*	*Stable*	*Herd*	*02.06.2017*	*Bumpeh*	M	*8*
SL 2017-543	Pool 29	neg	0,12	neg	*South*	*BO*	*Torwama Town*	*Swine*	*Large white*	*Farm bush*	*Stable*	*Herd*	*02.06.2017*	*Bumpeh*	M	*3*
SL 2017-544			0,15	neg	*South*	*BO*	*Torwama Town*	*Swine*	*Duroc*	*Farm bush*	*Stable*	*Herd*	*02.06.2017*	*Bumpeh*	F	*3*
SL 2017-545			0,14	neg	*South*	*BO*	*Torwama Town*	*Swine*	*Duroc*	*Farm bush*	*Stable*	*Herd*	*02.06.2017*	*Bumpeh*	F	*3*
SL 2017-546			0,25	neg	*South*	*BO*	*Torwama Town*	*Swine*	*Large white*	*Farm bush*	*Stable*	*Herd*	*02.06.2017*	*Bumpeh*	F	*3*
SL 2017-547			0,21	neg	*South*	*BO*	*Torwama Town*	*Swine*	*Large white*	*Farm bush*	*Stable*	*Herd*	*02.06.2017*	*Bumpeh*	F	*24*
SL 2017-548	Pool 30	neg	0,15	neg	*South*	*BO*	*Torwama Town*	*Swine*	*Large white*	*Farm bush*	*Stable*	*Herd*	*02.06.2017*	*Bumpeh*	F	*24*
SL 2017-549			0,21	neg	*South*	*BO*	*Torwama Town*	*Swine*	*Large white*	*Farm bush*	*Stable*	*Herd*	*02.06.2017*	*BO*	F	*8*

SL 2017-550			0,12	neg	South	BO	Torwama Town	Swine	Large white	Farm bush	Stable	Herd	02.06.2017	BO	F	8
SL 2017-551			0,23	neg	South	BO	Torwama Town	Swine	Large white	Farm bush	Stable	Herd	02.06.2017	BO	F	16
SL 2017-552			0,25	neg	South	BO	Torwama Town	Swine	Large white	Farm bush	Stable	Herd	02.06.2017	BO	F	16
SL 2017-553	Pool 31	neg	0,24	neg	South	BO	Torwama Town	Swine	Large white	Farm bush	Stable	Herd	02.06.2017	BO	F	14
SL 2017-554			0,22	neg	South	BO	Torwama Town	Swine	Large white	Farm bush	Stable	Herd	02.06.2017	BO	F	14
SL 2017-555			0,31	neg	South	BO	Torwama Town	Swine	Large white	Farm bush	Stable	Herd	02.06.2017	BO	M	3
SL 2017-556			0,19	neg	South	BO	Torwama Town	Swine	Large white	Farm bush	Stable	Herd	02.06.2017	BO	M	3
SL 2017-557			0,21	neg	South	BO	Torwama Town	Swine	Duroc	Farm bush	Stable	Herd	02.06.2017	BO	M	3
SL 2017-558	Pool 32	neg	0,22	neg	South	BO	Torwama Town	Swine	Duroc	Farm bush	Stable	Herd	02.06.2017	BO	M	3
SL 2017-559			0,17	neg	South	BO	Torwama Town	Swine	Duroc	Farm bush	Stable	Herd	02.06.2017	BO	M	3
SL 2017-560			0,25	neg	South	BO	Tikonko Town	Swine	Large white	Farm bush	Stable	Herd	02.06.2017	BO	M	3
SL 2017-561			0,17	neg	South	BO	Tikonko Town	Swine	Large white	Farm bush	Stable	Herd	02.06.2017	BO	M	3
SL 2017-562			0,14	neg	South	BO	Bo Town	Swine	Duroc	Farm bush	Stable	Herd	03.06.2017	BO	F	7
SL 2017-563	Pool 33	neg	0,22	neg	South	BO	Bo Town	Swine	Duroc	Farm bush	Stable	Herd	03.06.2017	BO	F	7
SL 2017-564			0,13	neg	South	BO	Bo Town	Swine	Duroc	Farm bush	Stable	Herd	03.06.2017	BO	F	7
SL 2017-565			0,17	neg	South	BO	Bo Town	Swine	Duroc	Farm bush	Stable	Herd	03.06.2017	BO	F	7
SL 2017-566			0,18	neg	South	BO	Bo Town	Swine	Duroc	Farm bush	Stable	Herd	03.06.2017	BO	F	12
SL 2017-567			0,21	neg	South	BO	Bo Town	Swine	Large white	Farm bush	Stable	Herd	03.06.2017	BO	F	12
SL 2017-568	Pool 34	neg	0,18	neg	South	BO	Bo Town	Swine	Duroc	Farm bush	Stable	Herd	03.06.2017	BO	F	12
SL 2017-569			0,16	neg	South	BO	Bo Town	Swine	Large white	Farm bush	Stable	Herd	03.06.2017	BO	F	12
SL 2017-570			0,19	neg	South	BO	Bo Town	Swine	Large white	Farm bush	Stable	Herd	03.06.2017	BO	F	14
SL 2017-571			0,16	neg	South	BO	Bo Town	Swine	Large white	Farm bush	Stable	Herd	03.06.2017	BO	F	8
SL 2017-572			0,17	neg	South	BO	Bo Town	Swine	Large white	Farm bush	Stable	Herd	03.06.2017	BO	F	12
SL 2017-573	Pool 35	neg	0,18	neg	South	BO	Bo Town	Swine	Large white	Farm bush	Stable	Herd	03.06.2017	BO	F	12
SL 2017-574			0,14	neg	South	BO	Bo Town	Swine	Duroc	Farm bush	Stable	Herd	03.06.2017	BO	F	3
SL 2017-575			0,15	neg	South	BO	Bo Town	Swine	Duroc	Farm bush	Stable	Herd	03.06.2017	BO	M	3
SL 2017-576			0,15	neg	South	BO	Bo Town	Swine	Duroc	Farm bush	Stable	Herd	03.06.2017	BO	M	3
SL 2017-577			0,16	neg	South	BO	Bo Town	Swine	Duroc	Farm bush	Stable	Herd	03.06.2017	BO	F	3
SL 2017-578	Pool 36	neg	0,17	neg	South	BO	Bo Town	Swine	Duroc	Farm bush	Stable	Herd	03.06.2017	BO	M	3
SL 2017-579			0,22	neg	South	BO	Bo Town	Swine	Duroc	Farm bush	Stable	Herd	03.06.2017	BO	M	3
SL 2017-580			0,15	neg	South	BO	Bo Town	Swine	Duroc	Farm bush	Stable	Herd	03.06.2017	BO	M	3
SL 2017-581			0,16	neg	South	BO	Bo Town	Swine	Duroc	Farm bush	Stable	Herd	03.06.2017	BO	F	3
SL 2017-582			0,15	neg	South	BO	Bo Town	Swine	Duroc	Farm bush	Stable	Herd	03.06.2017	BO	M	3
SL 2017-583	Pool 37	neg	0,23	neg	South	BO	Bo Town	Swine	Duroc	Farm bush	Stable	Herd	03.06.2017	BO	F	24
SL 2017-584			0,18	neg	South	BO	Bo Town	Swine	Duroc	Farm bush	Stable	Herd	03.06.2017	BO	M	6
SL 2017-585			0,21	neg	South	BO	Bo Town	Swine	Duroc	Farm bush	Stable	Herd	03.06.2017	BO	M	6
SL 2017-586			0,19	neg	South	BO	Bo Town	Swine	Duroc	Farm bush	Stable	Herd	03.06.2017	BO	M	6
SL 2017-587			0,26	neg	South	BO	Bo Town	Swine	Duroc	Farm bush	Stable	Herd	03.06.2017	BO	M	6
SL 2017-588	Pool 38	neg	0,13	neg	South	BO	Bo Town	Swine	Duroc	Farm bush	Stable	Herd	03.06.2017	BO	M	6
SL 2017-589			0,13	neg	South	BO	Bo Town	Swine	Duroc	Farm bush	Stable	Herd	03.06.2017	BO	M	6
SL 2017-590			0,13	neg	South	BO	Bo Town	Swine	Duroc	Farm bush	Stable	Herd	03.06.2017	BO	M	6
SL 2017-591			0,16	neg	South	BO	Bo Town	Swine	Large white	Farm bush	Stable	Herd	03.06.2017	BO	M	7
SL 2017-592			0,16	neg	South	BO	Bo Town	Swine	Large white	Farm bush	Stable	Herd	03.06.2017	BO	M	7
SL 2017-593	Pool 39	neg	0,16	neg	South	BO	Bo Town	Swine	Large white	Farm bush	Stable	Herd	03.06.2017	BO	M	7
SL 2017-594			0,16	neg	South	BO	Bo Town	Swine	Large white	Farm bush	Stable	Herd	03.06.2017	BO	M	7
SL 2017-595			0,14	neg	South	BO	Bo Town	Swine	Large white	Farm bush	Stable	Herd	03.06.2017	BO	M	7
SL 2017-596			0,18	neg	South	BO	Bo Town	Swine	Large white	Farm bush	Stable	Herd	03.06.2017	BO	M	7
SL 2017-597			0,14	neg	South	BO	Bo Town	Swine	Large white	Farm bush	Stable	Herd	03.06.2017	BO	M	7
SL 2017-598	Pool 40	neg	0,13	neg	South	BO	Bo Town	Swine	Large white	Farm bush	Stable	Herd	03.06.2017	BO	M	7
SL 2017-599			0,13	neg	South	BO	Bo Town	Swine	Large white	Farm bush	Stable	Herd	03.06.2017	BO	F	8
SL 2017-600			0,14	neg	South	BO	Bo Town	Swine	Large white	Farm bush	Stable	Herd	03.06.2017	BO	F	8
SL 2017-601			0,18	neg	South	BO	Bo Town	Swine	Large white	Farm bush	Stable	Herd	03.06.2017	BO	F	8
SL 2017-602			0,14	neg	South	BO	Bo Town	Swine	Large white	Farm bush	Stable	Herd	03.06.2017	BO	F	8
SL 2017-603	Pool 41	neg	0,16	neg	South	BO	Bo Town	Swine	Duroc	Farm bush	Stable	Herd	03.06.2017	BO	F	3
SL 2017-604			0,14	neg	South	BO	Bo Town	Swine	Duroc	Farm bush	Stable	Herd	03.06.2017	BO	F	3
SL 2017-605			0,13	neg	South	BO	Bo Town	Swine	Large white	Farm bush	Stable	Herd	03.06.2017	BO	M	3
SL 2017-606			0,14	neg	South	BO	Bo Town	Swine	Large white	Farm bush	Stable	Herd	03.06.2017	BO	F	3
SL 2017-607			0,16	neg	South	BO	Bo Town	Swine	Large white	Farm bush	Stable	Herd	03.06.2017	BO	F	3
SL 2017-608	Pool 42	neg	0,16	neg	South	BO	Bo Town	Swine	Large white	Farm bush	Stable	Herd	03.06.2017	BO	M	3
SL 2017-609			0,14	neg	South	BO	Bo Town	Swine	Large white	Farm bush	Stable	Herd	03.06.2017	BO	F	3
SL 2017-610			0,14	neg	South	BO	Bo Town	Swine	Duroc	Farm bush	Stable	Herd	03.06.2017	BO	F	38
SL 2017-611			0,38	neg	South	BO	Bo Town	Swine	Large white	Farm bush	Stable	Herd	03.06.2017	BO	F	14
SL 2017-612			0,22	neg	South	BO	Bo Town	Swine	Large white	Farm bush	Stable	Herd	03.06.2017	BO	F	12
SL 2017-613	Pool 43	neg	0,15	neg	South	BO	Bo Town	Swine	Large white	Farm bush	Stable	Herd	03.06.2017	BO	F	12
SL 2017-614			0,17	neg	South	BO	Bo Town	Swine	Large white	Farm bush	Stable	Herd	03.06.2017	BO	F	24
SL 2017-615			0,11	neg	South	BO	Bo Town	Swine	Large white	Farm bush	Stable	Herd	03.06.2017	BO	F	12
SL 2017-616			0,44	pos	South	BO	Bo Town	Swine	Large white	Farm bush	Stable	Herd	03.06.2017	BO	F	12
SL 2017-617			0,23	neg	South	BO	Bo Town	Swine	Large white	Farm bush	Stable	Herd	03.06.2017	BO	F	12
SL 2017-618	Pool 44	neg	0,20	neg	South	BO	Bo Town	Swine	Large white	Farm bush	Stable	Herd	03.06.2017	BO	F	14
SL 2017-619			0,26	neg	South	BO	Bo Town	Swine	Large white	Farm bush	Stable	Herd	03.06.2017	BO	F	20
SL 2017-620			0,20	neg	South	BO	Bo Town	Swine	Large white	Farm bush	Stable	Herd	03.06.2017	BO	F	20
SL 2017-621			0,19	neg	South	BO	Bo Town	Swine	Large white	Farm bush	Stable	Herd	03.06.2017	BO	F	20
SL 2017-622			0,14	neg	South	BO	Bo Town	Swine	Large white	Farm bush	Stable	Herd	03.06.2017	BO	M	10
SL 2017-623	Pool 45	neg	0,17	neg	South	BO	Bo Town	Swine	Large white	Farm bush	Stable	Herd	03.06.2017	BO	F	10
SL 2017-624			0,18	neg	South	BO	Bo Town	Swine	Large white	Farm bush	Stable	Herd	03.06.2017	BO	F	10
SL 2017-625			0,16	neg	South	BO	Bo Town	Swine	Large white	Farm bush	Stable	Herd	03.06.2017	BO	F	10
SL 2017-626			0,15	neg	South	BO	Bo Town	Swine	Large white	Farm bush	Stable	Herd	03.06.2017	BO	F	10
SL 2017-627			0,20	neg	South	BO	Bo Town	Swine	Duroc	Farm bush	Stable	Herd	03.06.2017	BO	M	26
SL 2017-628	Pool 46	neg	0,21	neg	South	BO	Bo Town	Swine	Duroc	Farm bush	Stable	Herd	03.06.2017	BO	M	30
SL 2017-629			0,19	neg	South	BO	Bo Town	Swine	Large white	Farm bush	Stable	Herd	03.06.2017	BO	F	30
SL 2017-630			0,18	neg	South	BO	Bo Town	Swine	Large white	Farm bush	Stable	Herd	04.06.2017	BO	M	18
SL 2017-631			0,24	neg	South	BO	Bo Town	Swine	Large white	Farm bush	Stable	Herd	04.06.2017	BO	M	21
SL 2017-632			0,17	neg	South	BO	Bo Town	Swine	Large white	Farm bush	Stable	Herd	04.06.2017	BO	F	21
SL 2017-633	Pool 47	neg	0,21	neg	South	BO	Bo Town	Swine	Large white	Farm bush	Stable	Herd	04.06.2017	BO	F	21
SL 2017-634			0,18	neg	South	BO	Bo Town	Swine	Large white	Farm bush	Stable	Herd	04.06.2017	BO	F	21
SL 2017-635			0,17	neg	South	BO	Bo Town	Swine	Large white	Farm bush	Stable	Herd	04.06.2017	BO	F	12
SL 2017-636			0,21	neg	South	BO	Bo Town	Swine	Large white	Farm bush	Stable	Herd	04.06.2017	BO	M	8
SL 2017-637			0,25	neg	South	BO	Bo Town	Swine	Large white	Farm bush	Stable	Herd	04.06.2017	Pujehun	M	18
SL 2017-638	Pool 48	neg	0,15	neg	South	BO	Bo Town	Swine	Large white	Farm bush	Stable	Herd	04.06.2017	BO	M	18

SL 2017-639			0,29	neg	South	BO	Bo Town	Swine	Large white	Farm bush	Stable	Herd	04.06.2017	BO	F	17
SL 2017-640			0,17	neg	South	BO	Bo Town	Swine	Large white	Farm bush	Stable	Herd	04.06.2017	BO	F	12
SL 2017-641			0,21	neg	South	BO	Bo Town	Swine	Large white	Farm bush	Stable	Herd	04.06.2017	BO	F	19
SL 2017-642			0,15	neg	South	BO	Bo Town	Swine	Large white	Farm bush	Stable	Herd	04.06.2017	BO	F	20
SL 2017-643	Pool 49	neg	0,12	neg	South	BO	Bo Town	Swine	Large white	Farm bush	Stable	Herd	04.06.2017	BO	F	8
SL 2017-644			0,16	neg	South	BO	Bo Town	Swine	Large white	Farm bush	Stable	Herd	04.06.2017	BO	F	8
SL 2017-645			0,13	neg	South	BO	Bo Town	Swine	Duroc	Farm bush	Stable	Herd	04.06.2017	BO	M	9
SL 2017-646			0,13	neg	South	BO	Bo Town	Swine	Duroc	Farm bush	Stable	Herd	04.06.2017	BO	M	9
SL 2017-647			0,12	neg	South	BO	Bo Town	Swine	Duroc	Farm bush	Stable	Herd	04.06.2017	BO	M	9
SL 2017-648	Pool 50	neg	0,13	neg	South	BO	Bo Town	Swine	Large white	Farm bush	Stable	Herd	04.06.2017	BO	M	10
SL 2017-649			0,15	neg	South	BO	Bo Town	Swine	Duroc	Farm bush	Stable	Herd	04.06.2017	BO	M	10
SL 2017-650			0,15	neg	South	BO	Bo Town	Swine	Duroc	Farm bush	Stable	Herd	04.06.2017	BO	M	10
SL 2017-661			0,30	neg	South	Moyamba	Bambobu	Swine	Large white	Farm bush	Stable	Herd	12.06.2017		F	60
SL 2017-662			0,18	neg	South	Moyamba	Bambobu	Swine	Large white	Farm bush	Stable	Herd	12.06.2017		F	60
SL 2017-663	Pool 51	neg	0,23	neg	South	Moyamba	Mojaka	Swine	Large white	Farm bush	Stable	Herd	12.06.2017		F	36
SL 2017-664			0,22	neg	South	Moyamba	Mojaka	Swine	Large white	Farm bush	Stable	Herd	12.06.2017		F	24
SL 2017-665			0,20	neg	South	Moyamba	Mojaka	Swine	Large white	Farm bush	Stable	Herd	12.06.2017		F	24
SL 2017-666			0,15	neg	South	Moyamba	Mojaka	Swine	Large white	Farm bush	Stable	Herd	12.06.2017		F	72
SL 2017-667			0,21	neg	South	Moyamba	Mojaka	Swine	Large white	Farm bush	Stable	Herd	12.06.2017		F	72
SL 2017-668	Pool 52	neg	0,19	neg	South	Moyamba	Levuma WAD	Swine	Large white	Farm bush	Stable	Herd	12.06.2017		F	12
SL 2017-669			0,15	neg	South	Moyamba	Levuma WAD	Swine	Large white	Farm bush	Stable	Herd	12.06.2017		F	20
SL 2017-670			0,15	neg	South	Moyamba	Levuma WAD	Swine	Large white	Farm bush	Stable	Herd	12.06.2017		M	16
SL 2017-671			0,13	neg	South	Moyamba	Levuma WAD	Swine	Large white	Farm bush	Stable	Herd	12.06.2017		M	19
SL 2017-672			0,14	neg	South	Moyamba	Levuma WAD	Swine	Large white	Farm bush	Stable	Herd	12.06.2017		M	21
SL 2017-673	Pool 53	neg	0,19	neg	South	Moyamba	Levuma WAD	Swine	Large white	Farm bush	Stable	Herd	12.06.2017		M	26
SL 2017-674			0,31	neg	South	Moyamba	Levuma WAD	Swine	Large white	Farm bush	Stable	Herd	12.06.2017		F	40
SL 2017-675			0,38	neg	South	Moyamba	Levuma WAD	Swine	Large white	Farm bush	Stable	Herd	12.06.2017		F	36
SL 2017-676			0,34	neg	South	Moyamba	Levuma WAD	Swine	Large white	Farm bush	Stable	Herd	12.06.2017		F	36
SL 2017-677			0,28	neg	South	Moyamba	Levuma WAD	Swine	Large white	Farm bush	Stable	Herd	12.06.2017		F	48
SL 2017-678	Pool 54	neg	0,23	neg	South	Moyamba	Levuma WAD	Swine	Large white	Farm bush	Stable	Herd	12.06.2017		F	36
SL 2017-679			0,16	neg	South	Moyamba	Levuma WAD	Swine	Large white	Farm bush	Stable	Herd	12.06.2017		F	12
SL 2017-680			0,18	neg	South	Moyamba	Levuma WAD	Swine	Large white	Farm bush	Stable	Herd	12.06.2017		M	9
SL 2017-681			0,19	neg	South	Moyamba	Levuma WAD	Swine	Large white	Farm bush	Stable	Herd	12.06.2017		F	12
SL 2017-682			0,16	neg	South	Moyamba	Levuma WAD	Swine	Large white	Farm bush	Stable	Herd	12.06.2017		M	12
SL 2017-683	Pool 55	neg	0,14	neg	South	Moyamba	Levuma WAD	Swine	Large white	Farm bush	Stable	Herd	12.06.2017		F	7
SL 2017-684			0,18	neg	South	Moyamba	Levuma WAD	Swine	Large white	Farm bush	Stable	Herd	12.06.2017		M	7
SL 2017-685			0,17	neg	South	Moyamba	Levuma WAD	Swine	Large white	Farm bush	Stable	Herd	12.06.2017		M	12
SL 2017-686			0,19	neg	South	Moyamba	Levuma WAD	Swine	Large white	Farm bush	Stable	Herd	12.06.2017		F	36
SL 2017-687			0,20	neg	South	Moyamba	Levuma WAD	Swine	Large white	Farm bush	Stable	Herd	12.06.2017		F	18
SL 2017-688	Pool 56	neg	0,11	neg	South	Moyamba	Lungi	Swine	Large white	Farm bush	Stable	Herd	12.06.2017		M	7
SL 2017-689			0,25	neg	South	Moyamba	Lungi	Swine	Large white	Farm bush	Stable	Herd	12.06.2017		F	12
SL 2017-690			0,16	neg	South	Moyamba	Lungi	Swine	Large white	Farm bush	Stable	Herd	12.06.2017		M	8
SL 2017-691			0,15	neg	South	Moyamba	Lungi	Swine	Large white	Farm bush	Stable	Herd	12.06.2017		M	6
SL 2017-692			0,25	neg	South	Moyamba	Lungi	Swine	Large white	Farm bush	Stable	Herd	12.06.2017		M	6
SL 2017-693	Pool 57	neg	0,27	neg	South	Moyamba	Lungi	Swine	Large white	Farm bush	Stable	Herd	12.06.2017		F	12
SL 2017-694			0,18	neg	South	Moyamba	Lungi	Swine	Large white	Farm bush	Stable	Herd	12.06.2017		F	9
SL 2017-695			0,77	pos	South	Moyamba	Lungi	Swine	Large white	Farm bush	Stable	Herd	12.06.2017		F	24
SL 2017-696			0,21	neg	South	Moyamba	Lungi	Swine	Large white	Farm bush	Stable	Herd	12.06.2017		F	18
SL 2017-697			0,14	neg	South	Moyamba	Lungi	Swine	Large white	Farm bush	Stable	Herd	12.06.2017		M	24
SL 2017-698	Pool 58	neg	0,25	neg	South	Moyamba	Lungi	Swine	Large white	Farm bush	Stable	Herd	12.06.2017		F	24
SL 2017-699			0,24	neg	South	Moyamba	Lungi	Swine	Large white	Farm bush	Stable	Herd	12.06.2017		F	24
SL 2017-700			0,20	neg	South	Moyamba	Lungi	Swine	Large white	Farm bush	Stable	Herd	12.06.2017		F	24
SL 2017-701			0,13	neg	South	Moyamba	Lungi	Swine	Large white	Farm bush	Stable	Herd	12.06.2017		F	6
SL 2017-702			0,14	neg	South	Moyamba	Lungi	Swine	Large white	Farm bush	Stable	Herd	12.06.2017		F	6
SL 2017-703	Pool 59	neg	0,24	neg	South	Moyamba	Lungi	Swine	Large white	Farm bush	Stable	Herd	12.06.2017		F	18
SL 2017-704			0,21	neg	South	Moyamba	Lungi	Swine	Large white	Farm bush	Stable	Herd	12.06.2017		F	18
SL 2017-705			0,15	neg	South	Moyamba	Moyamba Town	Swine	Large white	Farm bush	Stable	Herd	12.06.2017		F	6
SL 2017-706			0,11	neg	South	Moyamba	Moyamba Town	Swine	Large white	Farm bush	Stable	Herd	12.06.2017		M	8
SL 2017-707			0,14	neg	South	Moyamba	Moyamba Town	Swine	Large white	Farm bush	Stable	Herd	12.06.2017		M	8
SL 2017-708	Pool 60	neg	0,17	neg	South	Moyamba	Moyamba Town	Swine	Large white	Farm bush	Stable	Herd	12.06.2017		M	6
SL 2017-709			0,14	neg	South	Moyamba	Moyamba Town	Swine	Large white	Farm bush	Stable	Herd	12.06.2017		M	6
SL 2017-710			0,13	neg	South	Moyamba	Moyamba Town	Swine	Large white	Farm bush	Stable	Herd	12.06.2017		M	6
SL 2017-711			0,11	neg	South	Moyamba	Moyamba Town	Swine	Large white	Farm bush	Stable	Herd	12.06.2017		F	6
SL 2017-712			0,13	neg	South	Moyamba	Moyamba Town	Swine	Large white	Farm bush	Stable	Herd	12.06.2017		M	6
SL 2017-713	Pool 61	neg	0,12	neg	South	Moyamba	Moyamba Town	Swine	Large white	Farm bush	Stable	Herd	12.06.2017		F	8
SL 2017-714			0,12	neg	South	Moyamba	Moyamba Town	Swine	Large white	Farm bush	Stable	Herd	12.06.2017		F	8
SL 2017-715			0,12	neg	South	Moyamba	Moyamba Town	Swine	Large white	Farm bush	Stable	Herd	12.06.2017		F	8
SL 2017-716			0,11	neg	South	Moyamba	Moyamba Town	Swine	Large white	Farm bush	Stable	Herd	12.06.2017		F	12
SL 2017-717			0,12	neg	South	Moyamba	Moyamba Town	Swine	Large white	Farm bush	Stable	Herd	12.06.2017		F	12
SL 2017-718	Pool 62	neg	0,12	neg	South	Moyamba	Moyamba Town	Swine	Large white	Farm bush	Stable	Herd	12.06.2017		F	6
SL 2017-719			0,11	neg	South	Moyamba	Moyamba Town	Swine	Large white	Farm bush	Stable	Herd	12.06.2017		F	12
SL 2017-720			0,19	neg	South	Moyamba	Moyamba Town	Swine	Large white	Farm bush	Stable	Herd	12.06.2017		F	12
SL 2017-721			0,13	neg	South	Moyamba	Moyamba Town	Swine	Large white	Farm bush	Stable	Herd	12.06.2017		F	6
SL 2017-722			0,15	neg	South	Moyamba	Moyamba Town	Swine	Large white	Farm bush	Stable	Herd	12.06.2017		F	6
SL 2017-723	Pool 63	neg	0,12	neg	South	Moyamba	Moyamba Town	Swine	Large white	Farm bush	Stable	Herd	12.06.2017		F	8
SL 2017-724			0,17	neg	South	Moyamba	Moyamba Town	Swine	Large white	Farm bush	Stable	Herd	12.06.2017		F	8
SL 2017-725			0,15	neg	South	Moyamba	Moyamba Town	Swine	Large white	Farm bush	Stable	Herd	12.06.2017		F	9
SL 2017-726			0,15	neg	South	Moyamba	Moyamba Town	Swine	Large white	Farm bush	Stable	Herd	12.06.2017		F	9
SL 2017-727			0,11	neg	South	Moyamba	Moyamba Town	Swine	Large white	Farm bush	Stable	Herd	12.06.2017		F	12
SL 2017-728	Pool 64	neg	0,11	neg	South	Moyamba	Moyamba Town	Swine	Large white	Farm bush	Stable	Herd	12.06.2017		F	12
SL 2017-729			0,12	neg	South	Moyamba	Moyamba Town	Swine	Large white	Farm bush	Stable	Herd	12.06.2017		M	12
SL 2017-730			0,11	neg	South	Moyamba	Moyamba Town	Swine	Large white	Farm bush	Stable	Herd	12.06.2017		M	12
SL 2017-731			0,10	neg	South	Moyamba	Moyamba Town	Swine	Large white	Farm bush	Stable	Herd	12.06.2017		M	12
SL 2017-732			0,16	neg	South	Moyamba	Moyamba Town	Swine	Large white	Farm bush	Stable	Herd	12.06.2017		M	6
SL 2017-733	Pool 65	neg	0,17	neg	South	Moyamba	Moyamba Town	Swine	Large white	Farm bush	Stable	Herd	12.06.2017		M	6
SL 2017-734			0,14	neg	South	Moyamba	Moyamba Town	Swine	Large white	Farm bush	Stable	Herd	12.06.2017		M	6
SL 2017-735			0,12	neg	South	Moyamba	Moyamba Town	Swine	Large white	Farm bush	Stable	Herd	12.06.2017		M	6
SL 2017-736			0,14	neg	South	Moyamba	Moyamba Town	Swine	Large white	Farm bush	Stable	Herd	12.06.2017	Bonthe	F	6
SL 2017-737			0,15	neg	South	Moyamba	Moyamba Town	Swine	Large white	Farm bush	Stable	Herd	12.06.2017	Bonthe	F	6

SL 2017-738	Pool 66	neg	0,14	neg	*South*	*Moyamba*	*Moyamba Town*	*Swine*	*Large white*	*Farm bush*	*Stable*	*Herd*	*12.06.2017*	*Bonthe*	*F*	*6*
SL 2017-739			0,14	neg	*South*	*Moyamba*	*Moyamba Town*	*Swine*	*Large white*	*Farm bush*	*Stable*	*Herd*	*12.06.2017*	*Bonthe*	*M*	*6*
SL 2017-740			0,13	neg	*South*	*Moyamba*	*Moyamba Town*	*Swine*	*Large white*	*Farm bush*	*Stable*	*Herd*	*12.06.2017*	*Bonthe*	*M*	*8*
SL 2017-741			0,12	neg	*South*	*Moyamba*	*Moyamba Town*	*Swine*	*Large white*	*Farm bush*	*Stable*	*Herd*	*12.06.2017*	*Bonthe*	*F*	*7*
SL 2017-742			0,13	neg	*South*	*Moyamba*	*Moyamba Town*	*Swine*	*Large white*	*Farm bush*	*Stable*	*Herd*	*12.06.2017*	*Bonthe*	*F*	*8*
SL 2017-743	Pool 67	neg	0,22	neg	*South*	*Moyamba*	*Moyamba Town*	*Swine*	*Large white*	*Farm bush*	*Stable*	*Herd*	*12.06.2017*	*Bonthe*	*F*	*7*
SL 2017-744			0,13	neg	*South*	*Moyamba*	*Moyamba Town*	*Swine*	*Large white*	*Farm bush*	*Stable*	*Herd*	*12.06.2017*	*Bonthe*	*F*	*7*
SL 2017-745			0,12	neg	*South*	*Moyamba*	*Moyamba Town*	*Swine*	*Large white*	*Farm bush*	*Stable*	*Herd*	*12.06.2017*	*Bonthe*	*M*	*7*
SL 2017-746			0,15	neg	*South*	*Moyamba*	*Moyamba Town*	*Swine*	*Large white*	*Farm bush*	*Stable*	*Herd*	*12.06.2017*	*Bonthe*	*M*	*7*
SL 2017-747			0,15	neg	*South*	*Moyamba*	*Moyamba Town*	*Swine*	*Large white*	*Farm bush*	*Stable*	*Herd*	*12.06.2017*	*Bonthe*	*F*	*6*
SL 2017-748	Pool 68	neg	0,14	neg	*South*	*Moyamba*	*Moyamba Town*	*Swine*	*Large white*	*Farm bush*	*Stable*	*Herd*	*12.06.2017*	*Bonthe*	*F*	*6*
SL 2017-749			0,13	neg	*South*	*Moyamba*	*Moyamba Town*	*Swine*	*Large white*	*Farm bush*	*Stable*	*Herd*	*12.06.2017*	*Bonthe*	*F*	*12*
SL 2017-750			0,13	neg	*South*	*Moyamba*	*Moyamba Town*	*Swine*	*Large white*	*Farm bush*	*Stable*	*Herd*	*12.06.2017*	*Bonthe*	*F*	*12*
SL 2017-751			0,13	neg	*South*	*Moyamba*	*Moyamba Town*	*Swine*	*Large white*	*Farm bush*	*Stable*	*Herd*	*12.06.2017*	*Bonthe*	*M*	*21*
SL 2017-752			0,17	neg	*South*	*Moyamba*	*Moyamba Town*	*Swine*	*Large white*	*Farm bush*	*Stable*	*Herd*	*12.06.2017*	*Bonthe*	*F*	*24*
SL 2017-753	Pool 69	neg	0,13	neg	*South*	*Moyamba*	*Moyamba Town*	*Swine*	*Large white*	*Farm bush*	*Stable*	*Herd*	*12.06.2017*	*Bonthe*	*F*	*24*
SL 2017-754			0,15	neg	*South*	*Moyamba*	*Moyamba Town*	*Swine*	*Large white*	*Farm bush*	*Stable*	*Herd*	*12.06.2017*	*Bonthe*	*M*	*24*
SL 2017-755			0,14	neg	*South*	*Moyamba*	*Moyamba Town*	*Swine*	*Large white*	*Farm bush*	*Stable*	*Herd*	*12.06.2017*	*Bonthe*	*M*	*18*
SL 2017-756			0,14	neg	*South*	*Moyamba*	*Moyamba Town*	*Swine*	*Large white*	*Farm bush*	*Stable*	*Herd*	*12.06.2017*	*Bonthe*	*M*	*12*
SL 2017-757			0,19	neg	*South*	*Moyamba*	*Moyamba Town*	*Swine*	*Large white*	*Farm bush*	*Stable*	*Herd*	*12.06.2017*	*Bonthe*	*F*	*12*
SL 2017-758	Pool 70	neg	0,18	neg	*South*	*Moyamba*	*Moyamba Town*	*Swine*	*Large white*	*Farm bush*	*Stable*	*Herd*	*12.06.2017*	*Bonthe*	*F*	*12*
SL 2017-759			0,14	neg	*South*	*Moyamba*	*Moyamba Town*	*Swine*	*Large white*	*Farm bush*	*Stable*	*Herd*	*12.06.2017*	*Bonthe*	*F*	*12*
SL 2017-760			0,19	neg	*South*	*Moyamba*	*Moyamba Town*	*Swine*	*Large white*	*Farm bush*	*Stable*	*Herd*	*12.06.2017*	*Bonthe*	*F*	*18*
SL 2017-761			0,15	neg	*South*	*Moyamba*	*Moyamba Town*	*Swine*	*Large white*	*Farm bush*	*Stable*	*Herd*	*12.06.2017*	*Bonthe*	*M*	*18*
SL 2017-762			0,11	neg	*South*	*Moyamba*	*Moyamba Town*	*Swine*	*Large white*	*Farm bush*	*Stable*	*Herd*	*12.06.2017*	*Bonthe*	*M*	*18*
SL 2017-763	Pool 71	neg	0,18	neg	*South*	*Moyamba*	*Moyamba Town*	*Swine*	*Large white*	*Farm bush*	*Stable*	*Herd*	*12.06.2017*	*Bonthe*	*F*	*6*
SL 2017-764			0,13	neg	*South*	*Moyamba*	*Moyamba Town*	*Swine*	*Large white*	*Farm bush*	*Stable*	*Herd*	*12.06.2017*	*Bonthe*	*F*	*6*
SL 2017-765			0,22	neg	*South*	*Moyamba*	*Moyamba Town*	*Swine*	*Large white*	*Farm bush*	*Stable*	*Herd*	*12.06.2017*	*Bonthe*	*F*	*6*
SL 2017-766			0,56	neg	*South*	*Moyamba*	*Moyamba Town*	*Swine*	*Large white*	*Farm bush*	*Stable*	*Herd*	*12.06.2017*	*Bonthe*	*M*	*6*
SL 2017-767			0,35	neg	*South*	*Moyamba*	*Moyamba Town*	*Swine*	*Large white*	*Farm bush*	*Stable*	*Herd*	*12.06.2017*	*Bonthe*	*F*	*6*
SL 2017-768	Pool 72	neg	0,18	neg	*South*	*Moyamba*	*Moyamba Town*	*Swine*	*Large white*	*Farm bush*	*Stable*	*Herd*	*12.06.2017*	*Bonthe*	*F*	*12*
SL 2017-769			0,23	neg	*South*	*Moyamba*	*Mokorewo*	*Swine*	*West Africa Duroc*	*Farm bush*	*stationary*	*Herd*	*15.07.2017*	*Bauya town*	*F*	*3*
SL 2017-770			0,35	neg	*South*	*Moyamba*	*Mokorewo*	*Swine*	*West Africa Duroc*	*Farm bush*	*stationary*	*Herd*	*15.07.2017*	*Bauya town*	*F*	*3*
SL 2017-771			0,36	neg	*South*	*Moyamba*	*Mokorewo*	*Swine*	*West Africa Duroc*	*Farm bush*	*stationary*	*Herd*	*15.07.2017*	*Bauya town*	*F*	*3*
SL 2017-772			0,20	neg	*South*	*Moyamba*	*Mokorewo*	*Swine*	*West Africa Duroc*	*Farm bush*	*stationary*	*Herd*	*15.07.2017*	*Bauya town*	*M*	*3*
SL 2017-773	Pool 73	neg	0,22	neg	*South*	*Moyamba*	*Mokorewo*	*Swine*	*West Africa Duroc*	*Farm bush*	*stationary*	*Herd*	*15.07.2017*	*Bauya town*	*F*	*3*
SL 2017-774			0,19	neg	*South*	*Moyamba*	*Mokorewo*	*Swine*	*West Africa Duroc*	*Farm bush*	*stationary*	*Herd*	*15.07.2017*	*Bauya town*	*F*	*3*
SL 2017-775			0,18	neg	*South*	*Moyamba*	*Mokorewo*	*Swine*	*West Africa Duroc*	*Farm bush*	*stationary*	*Herd*	*15.07.2017*	*Bauya town*	*F*	*3*
SL 2017-776			0,20	neg	*South*	*Moyamba*	*Mokorewo*	*Swine*	*West Africa Duroc*	*Farm bush*	*stationary*	*Herd*	*15.07.2017*	*Bauya town*	*M*	*3*
SL 2017-777			0,22	neg	*South*	*Moyamba*	*Mokorewo*	*Swine*	*West Africa Duroc*	*Farm bush*	*stationary*	*Herd*	*15.07.2017*	*Bauya town*	*F*	*3*
SL 2017-778	Pool 74	neg	0,19	neg	*South*	*Moyamba*	*Mokorewo*	*Swine*	*West Africa Duroc*	*Farm bush*	*stationary*	*Herd*	*15.07.2017*	*Bauya town*	*M*	*3*
SL 2017-779			0,26	neg	*South*	*Moyamba*	*Mokorewo*	*Swine*	*West Africa Duroc*	*Farm bush*	*stationary*	*Herd*	*15.07.2017*	*Bauya town*	*F*	*8*
SL 2017-780			0,37	neg	*South*	*Moyamba*	*Mokorewo*	*Swine*	*West Africa Duroc*	*Farm bush*	*stationary*	*Herd*	*15.07.2017*	*Bauya town*	*F*	*8*
SL 2017-781			0,41	neg	*South*	*Moyamba*	*Mokorewo*	*Swine*	*Large white*	*Farm bush*	*stationary*	*Herd*	*15.07.2017*	*Bauya town*	*M*	*8*
SL 2017-782			0,33	neg	*South*	*Moyamba*	*Mokorewo*	*Swine*	*Large white*	*Farm bush*	*stationary*	*Herd*	*15.07.2017*	*Bauya town*	*M*	*24*
SL 2017-783	Pool 75	neg	0,18	neg	*South*	*Moyamba*	*Moyamba Town*	*Swine*	*Large white*	*Farm bush*	*stationary*	*Herd*	*15.07.2017*	*Bauya town*	*F*	*3*
SL 2017-784			0,19	neg	*South*	*Moyamba*	*Moyamba Town*	*Swine*	*Large white*	*Farm bush*	*stationary*	*Herd*	*15.07.2017*	*Bauya town*	*F*	*3*
SL 2017-785			0,19	neg	*South*	*Moyamba*	*Moyamba Town*	*Swine*	*Large white*	*Farm bush*	*stationary*	*Herd*	*15.07.2017*	*Bauya town*	*M*	*3*
SL 2017-786			0,19	neg	*South*	*Moyamba*	*Moyamba Town*	*Swine*	*Large white*	*Farm bush*	*stationary*	*Herd*	*15.07.2017*	*Bauya town*	*M*	*3*
SL 2017-787			0,21	neg	*South*	*Moyamba*	*Moyamba Town*	*Swine*	*Large white*	*Farm bush*	*stationary*	*Herd*	*15.07.2017*	*Bauya town*	*M*	*3*
SL 2017-788	Pool 76	neg	0,34	neg	*South*	*Moyamba*	*Moyamba Town*	*Swine*	*Large white*	*Farm bush*	*stationary*	*Herd*	*15.07.2017*	*Bauya town*	*M*	*24*
SL 2017-789			0,45	neg	*South*	*Moyamba*	*Moyamba Town*	*Swine*	*Large white*	*Farm bush*	*stationary*	*Herd*	*15.07.2017*	*Bauya town*	*F*	*24*
SL 2017-790			0,22	neg	*South*	*Moyamba*	*Moyamba Town*	*Swine*	*Large white*	*Farm bush*	*stationary*	*Herd*	*15.07.2017*	*Bauya town*	*F*	*24*
SL 2017-791			0,17	neg	*South*	*Moyamba*	*Moyamba Town*	*Swine*	*Large white*	*Farm bush*	*stationary*	*Herd*	*15.07.2017*	*Bauya town*	*M*	*5*
SL 2017-792			0,18	neg	*South*	*Moyamba*	*Moyamba Town*	*Swine*	*Large white*	*Farm bush*	*stationary*	*Herd*	*15.07.2017*	*Bauya town*	*M*	*3*
SL 2017-793	Pool 77	neg	0,18	neg	*South*	*Moyamba*	*Moyamba Town*	*Swine*	*Large white*	*Farm bush*	*stationary*	*Herd*	*15.07.2017*	*Bauya town*	*M*	*3*
SL 2017-794			0,18	neg	*South*	*Moyamba*	*Moyamba Town*	*Swine*	*Large white*	*Farm bush*	*stationary*	*Herd*	*15.07.2017*	*Bauya town*	*F*	*3*
SL 2017-795			0,18	neg	*South*	*Moyamba*	*Moyamba Town*	*Swine*	*Large white*	*Farm bush*	*stationary*	*Herd*	*15.07.2017*	*Bauya town*	*F*	*3*
SL 2017-796			0,25	neg	*South*	*Moyamba*	*Moyamba Town*	*Swine*	*Large white*	*Farm bush*	*stationary*	*Herd*	*15.07.2017*	*Bauya town*	*F*	*24*
SL 2017-797			0,45	neg	*South*	*Moyamba*	*Mokojo*	*Swine*	*West Africa Duroc*	*Farm bush*	*stationary*	*Herd*	*15.07.2017*	*Bauya town*	*F*	*8*
SL 2017-798	Pool 78	neg	0,24	neg	*South*	*Moyamba*	*Mokojo*	*Swine*	*West Africa Duroc*	*Farm bush*	*stationary*	*Herd*	*15.07.2017*	*Bauya town*	*M*	*8*
SL 2017-799			0,33	neg	*South*	*Moyamba*	*Mokojo*	*Swine*	*West Africa Duroc*	*Farm bush*	*stationary*	*Herd*	*15.07.2017*	*Bauya town*	*M*	*8*
SL 2017-800			0,32	neg	*South*	*Moyamba*	*Mokojo*	*Swine*	*West Africa Duroc*	*Farm bush*	*stationary*	*Herd*	*15.07.2017*	*Bauya town*	*F*	*8*
SL 2017-801			0,78	pos	*South*	*Moyamba*	*Mokojo*	*Swine*	*West Africa Duroc*	*Farm bush*	*stationary*	*Herd*	*15.07.2017*	*Bauya town*	*F*	*8*
SL 2017-802			0,22	neg	*South*	*Moyamba*	*Mokojo*	*Swine*	*West Africa Duroc*	*Farm bush*	*stationary*	*Herd*	*15.07.2017*	*Bauya town*	*M*	*8*
SL 2017-803	Pool 79	neg	0,26	neg	*South*	*Moyamba*	*Mokojo*	*Swine*	*West Africa Duroc*	*Farm bush*	*stationary*	*Herd*	*15.07.2017*	*Bauya town*	*F*	*18*
SL 2017-804			0,26	neg	*South*	*Moyamba*	*Mokojo*	*Swine*	*West Africa Duroc*	*Farm bush*	*stationary*	*Herd*	*15.07.2017*	*Bauya town*	*M*	*8*
SL 2017-805			0,26	neg	*South*	*Moyamba*	*Mokojo*	*Swine*	*West Africa Duroc*	*Farm bush*	*stationary*	*Herd*	*15.07.2017*	*Bauya town*	*F*	*8*
SL 2017-806			0,28	neg	*South*	*Moyamba*	*Mokojo*	*Swine*	*West Africa Duroc*	*Farm bush*	*stationary*	*Herd*	*15.07.2017*	*Bauya town*	*F*	*8*
SL 2017-807			0,29	neg	*South*	*Moyamba*	*Mokojo*	*Swine*	*West Africa Duroc*	*Farm bush*	*stationary*	*Herd*	*15.07.2017*	*Bauya town*	*F*	*8*
SL 2017-808	Pool 80	neg	0,20	neg	*South*	*Moyamba*	*Mortemne*	*Swine*	*West Africa Duroc*	*Farm bush*	*stationary*	*Herd*	*15.07.2017*	*Yoyema*	*F*	*5*
SL 2017-809			0,24	neg	*South*	*Moyamba*	*Mortemne*	*Swine*	*West Africa Duroc*	*Farm bush*	*stationary*	*Herd*	*15.07.2017*	*Yoyema*	*M*	*7*
SL 2017-810			0,29	neg	*South*	*Moyamba*	*Mortemne*	*Swine*	*West Africa Duroc*	*Farm bush*	*stationary*	*Herd*	*15.07.2017*	*Yoyema*	*F*	*6*
SL 2017-811			0,20	neg	*South*	*Moyamba*	*Moyamba Town*	*Swine*	*West Africa Duroc*	*Farm bush*	*stationary*	*Herd*	*15.07.2017*	*Njagbahun*	*F*	*6*
SL 2017-812			0,20	neg	*South*	*Moyamba*	*Moyamba Town*	*Swine*	*West Africa Duroc*	*Farm bush*	*stationary*	*Herd*	*15.07.2017*	*Njagbahun*	*F*	*8*
SL 2017-813	Pool 81	neg	0,36	neg	*South*	*Moyamba*	*Moyamba Town*	*Swine*	*West Africa Duroc*	*Farm bush*	*stationary*	*Herd*	*15.07.2017*	*Njagbahun*	*F*	*24*
SL 2017-814			0,22	neg	*South*	*Moyamba*	*Limba Corner*	*Swine*	*West Africa Duroc*	*Farm bush*	*stationary*	*Herd*	*16.07.2017*	*Wonde Mokonde*	*F*	*8*
SL 2017-815			0,37	neg	*South*	*Moyamba*	*Limba Corner*	*Swine*	*West Africa Duroc*	*Farm bush*	*stationary*	*Herd*	*16.07.2017*	*Wonde Mokonde*	*F*	*8*
SL 2017-816			0,41	neg	*South*	*Moyamba*	*Limba Corner*	*Swine*	*West Africa Duroc*	*Farm bush*	*stationary*	*Herd*	*16.07.2017*	*Wonde Mokonde*	*M*	*8*
SL 2017-817			0,24	neg	*South*	*Moyamba*	*Limba Corner*	*Swine*	*West Africa Duroc*	*Farm bush*	*stationary*	*Herd*	*16.07.2017*	*Wonde Mokonde*	*F*	*6*
SL 2017-818	Pool 82	neg	0,23	neg	*South*	*Moyamba*	*Limba Corner*	*Swine*	*West Africa Duroc*	*Farm bush*	*stationary*	*Herd*	*16.07.2017*	*Wonde Mokonde*	*F*	*10*
SL 2017-819			0,25	neg	*South*	*Moyamba*	*Limba Corner*	*Swine*	*West Africa Duroc*	*Farm bush*	*stationary*	*Herd*	*16.07.2017*	*Wonde Mokonde*	*F*	*10*
SL 2017-820			0,21	neg	*South*	*Moyamba*	*Limba Corner*	*Swine*	*West Africa Duroc*	*Farm bush*	*stationary*	*Herd*	*16.07.2017*	*Wonde Mokonde*	*F*	*12*
SL 2017-821			0,29	neg	*South*	*Moyamba*	*Limba Corner*	*Swine*	*West Africa Duroc*	*Farm bush*	*stationary*	*Herd*	*16.07.2017*	*Wonde Mokonde*	*M*	*10*
SL 2017-822			0,31	neg	*South*	*Moyamba*	*Limba Corner*	*Swine*	*West Africa Duroc*	*Farm bush*	*stationary*	*Herd*	*16.07.2017*	*Wonde Mokonde*	*M*	*3*
SL 2017-823	Pool 83	neg	0,28	neg	*South*	*Moyamba*	*Limba Corner*	*Swine*	*West Africa Duroc*	*Farm bush*	*stationary*	*Herd*	*16.07.2017*	*Wonde Mokonde*	*M*	*3*
SL 2017-824			0,15	neg	*South*	*Moyamba*	*Limba Corner*	*Swine*	*West Africa Duroc*	*Farm bush*	*stationary*	*Herd*	*16.07.2017*	*Wonde Mokonde*	*M*	*3*
SL 2017-825			0,29	neg	*South*	*Moyamba*	*Limba Corner*	*Swine*	*West Africa Duroc*	*Farm bush*	*stationary*	*Herd*	*16.07.2017*	*Wonde Mokonde*	*F*	*3*
SL 2017-826			0,18	neg	*South*	*Moyamba*	*Limba Corner*	*Swine*	*West Africa Duroc*	*Farm bush*	*stationary*	*Herd*	*16.07.2017*	*Wonde Mokonde*	*M*	*3*

SL 2017-827	Pool 84	neg	0,20	neg	South	Moyamba	Limba Corner	Swine	West Africa Duroc	Farm bush	stationary	Herd	16.07.2017	Wonde Mokonde	F	3
SL 2017-828			0,23	neg	South	Moyamba	Mowoto	Swine	West Africa Duroc	Farm bush	stationary	Herd	16.07.2017	Mowoto	M	5
SL 2017-829			0,31	neg	South	Moyamba	Mowoto	Swine	West Africa Duroc	Farm bush	stationary	Herd	16.07.2017	Mowoto	F	5
SL 2017-830			0,24	neg	South	Moyamba	Mowoto	Swine	West Africa Duroc	Farm bush	stationary	Herd	16.07.2017	Mowoto	F	6
SL 2017-831	Pool 85	neg	0,41	neg	South	Moyamba	Mowoto	Swine	West Africa Duroc	Farm bush	stationary	Herd	16.07.2017	Mowoto	F	6
SL 2017-832			0,22	neg	South	Moyamba	Mowoto	Swine	West Africa Duroc	Farm bush	stationary	Herd	16.07.2017	Mowoto	F	12
SL 2017-833			0,29	neg	South	Moyamba	Mowoto	Swine	West Africa Duroc	Farm bush	stationary	Herd	16.07.2017	Mowoto	M	6
SL 2017-834			0,30	neg	South	Moyamba	Mowoto	Swine	West Africa Duroc	Farm bush	stationary	Herd	16.07.2017	Mowoto	F	6
SL 2017-835			0,65	neg	South	Moyamba	Mowoto	Swine	West Africa Duroc	Farm bush	stationary	Herd	16.07.2017	Mowoto	F	12
SL 2017-836	Pool 86	neg	0,25	neg	South	Moyamba	Mowoto	Swine	West Africa Duroc	Farm bush	stationary	Herd	16.07.2017	Mowoto	F	14
SL 2017-837			0,29	neg	South	Moyamba	Mowoto	Swine	West Africa Duroc	Farm bush	stationary	Herd	16.07.2017	Mowoto	F	14
SL 2017-838			0,24	neg	South	Moyamba	Mowoto	Swine	West Africa Duroc	Farm bush	stationary	Herd	16.07.2017	Mowoto	F	3
SL 2017-839			0,41	neg	South	Moyamba	Mowoto	Swine	West Africa Duroc	Farm bush	stationary	Herd	16.07.2017	Mowoto	F	12
SL 2017-840			0,22	neg	South	Moyamba	Mowoto	Swine	West Africa Duroc	Farm bush	stationary	Herd	16.07.2017	Mowoto	F	3
SL 2017-841	Pool 87	neg	0,29	neg	South	Moyamba	Mowoto	Swine	West Africa Duroc	Farm bush	stationary	Herd	16.07.2017	Mowoto	F	12
SL 2017-842			0,30	neg	South	Moyamba	Mowoto	Swine	West Africa Duroc	Farm bush	stationary	Herd	16.07.2017	Mowoto	F	12
SL 2017-843			0,65	pos	South	Moyamba	Mowoto	Swine	West Africa Duroc	Farm bush	stationary	Herd	16.07.2017	Mowoto	M	10
SL 2017-844			0,25	neg	South	Moyamba	Mowoto	Swine	West Africa Duroc	Farm bush	stationary	Herd	16.07.2017	Mowoto	F	12
SL 2017-845			0,29	neg	South	Moyamba	Mowoto	Swine	West Africa Duroc	Farm bush	stationary	Herd	16.07.2017	Mowoto	M	3
SL 2017-846	Pool 88	neg	0,33	neg	South	Moyamba	Mowoto	Swine	West Africa Duroc	Farm bush	stationary	Herd	16.07.2017	Mowoto	M	3
SL 2017-847			0,26	neg	South	Moyamba	Mowoto	Swine	West Africa Duroc	Farm bush	stationary	Herd	16.07.2017	Mowoto	F	3
SL 2017-848			0,36	neg	South	Moyamba	Mowoto	Swine	West Africa Duroc	Farm bush	stationary	Herd	16.07.2017	Mowoto	M	3
SL 2017-849			0,19	neg	South	Moyamba	Mowoto	Swine	West Africa Duroc	Farm bush	stationary	Herd	16.07.2017	Mowoto	F	6
SL 2017-850			0,19	neg	South	Moyamba	Mowoto	Swine	West Africa Duroc	Farm bush	stationary	Herd	16.07.2017	Mowoto	M	3
SL 2017-851	Pool 89	neg	0,51	neg	South	Moyamba	Mowoto	Swine	West Africa Duroc	Farm bush	stationary	Herd	16.07.2017	Mowoto	M	6
SL 2017-852			0,30	neg	South	Moyamba	Mowoto	Swine	West Africa Duroc	Farm bush	stationary	Herd	16.07.2017	Mowoto	M	12
SL 2017-853			0,24	neg	South	Moyamba	Mowoto	Swine	West Africa Duroc	Farm bush	stationary	Herd	16.07.2017	Mowoto	F	14
SL 2017-854			0,35	neg	South	Moyamba	Mowoto	Swine	West Africa Duroc	Farm bush	stationary	Herd	16.07.2017	Mowoto	M	6
SL 2017-855			0,32	neg	South	Moyamba	Mowoto	Swine	West Africa Duroc	Farm bush	stationary	Herd	16.07.2017	Mowoto	M	6
SL 2017-856	Pool 90	neg	0,22	neg	South	Moyamba	Mowoto	Swine	West Africa Duroc	Farm bush	stationary	Herd	16.07.2017	Mowoto	M	6
SL 2017-857			0,26	neg	South	Moyamba	Mowoto	Swine	West Africa Duroc	Farm bush	stationary	Herd	16.07.2017	Mowoto	M	6
SL 2017-858			0,21	neg	South	Moyamba	Mowoto	Swine	West Africa Duroc	Farm bush	stationary	Herd	16.07.2017	Mowoto	M	6
SL 2017-859			0,38	neg	South	Moyamba	Mowoto	Swine	West Africa Duroc	Farm bush	stationary	Herd	16.07.2017	Mowoto	F	3
SL 2017-860			0,29	neg	South	Moyamba	Mowoto	Swine	West Africa Duroc	Farm bush	stationary	Herd	16.07.2017	Mowoto	M	3
SL 2017-861	Pool 91	neg	0,35	neg	South	Moyamba	Mowoto	Swine	West Africa Duroc	Farm bush	stationary	Herd	16.07.2017	Mowoto	M	3
SL 2017-862			0,29	neg	South	Moyamba	Mowoto	Swine	West Africa Duroc	Farm bush	stationary	Herd	16.07.2017	Mowoto	M	3
SL 2017-863			0,25	neg	South	Moyamba	Mowoto	Swine	West Africa Duroc	Farm bush	stationary	Herd	16.07.2017	Mowoto	M	3
SL 2017-864			0,24	neg	South	Moyamba	Mowoto	Swine	West Africa Duroc	Farm bush	stationary	Herd	16.07.2017	Mowoto	M	3
SL 2017-865			0,37	neg	South	Moyamba	Mowoto	Swine	West Africa Duroc	Farm bush	stationary	Herd	16.07.2017	Mowoto	F	6
SL 2017-866	Pool 92	neg	0,27	neg	South	Moyamba	Mowoto	Swine	West Africa Duroc	Farm bush	stationary	Herd	16.07.2017	Mowoto	F	6
SL 2017-867			0,25	neg	South	Moyamba	Mowoto	Swine	West Africa Duroc	Farm bush	stationary	Herd	16.07.2017	Mowoto	F	6
SL 2017-868			0,37	neg	South	Moyamba	Mowoto	Swine	West Africa Duroc	Farm bush	stationary	Herd	16.07.2017	Mowoto	F	6
SL 2017-869			0,34	neg	South	Moyamba	Mowoto	Swine	West Africa Duroc	Farm bush	stationary	Herd	16.07.2017	Mowoto	F	6
SL 2017-870			0,42	neg	South	Moyamba	Mowoto	Swine	West Africa Duroc	Farm bush	stationary	Herd	16.07.2017	Mowoto	F	6
SL 2017-871	Pool 93	neg	0,36	neg	South	Moyamba	Mowoto	Swine	West Africa Duroc	Farm bush	stationary	Herd	16.07.2017	Mowoto	F	3
SL 2017-872			0,73	pos	South	Moyamba	Mowoto	Swine	West Africa Duroc	Farm bush	stationary	Herd	16.07.2017	Mowoto	F	6
SL 2017-873			0,68	pos	South	Moyamba	Mowoto	Swine	West Africa Duroc	Farm bush	stationary	Herd	16.07.2017	Mowoto	F	10
SL 2017-874			0,45	neg	South	Moyamba	Mowoto	Swine	West Africa Duroc	Farm bush	stationary	Herd	16.07.2017	Mowoto	F	12
SL 2017-875			0,34	neg	South	Moyamba	Mowoto	Swine	West Africa Duroc	Farm bush	stationary	Herd	16.07.2017	Mowoto	F	12
SL 2017-876	Pool 94	neg	0,54	neg	South	Moyamba	Mowoto	Swine	West Africa Duroc	Farm bush	stationary	Herd	16.07.2017	Mowoto	F	6
SL 2017-877			0,24	neg	South	Moyamba	Mowoto	Swine	West Africa Duroc	Farm bush	stationary	Herd	16.07.2017	Mowoto	M	3
SL 2017-878			0,44	neg	South	Moyamba	Mowoto	Swine	West Africa Duroc	Farm bush	stationary	Herd	16.07.2017	Mowoto	F	3
SL 2017-879			0,24	neg	South	Moyamba	Mowoto	Swine	West Africa Duroc	Farm bush	stationary	Herd	16.07.2017	Mowoto	F	12
SL 2017-880			0,27	neg	South	Moyamba	Mowoto	Swine	West Africa Duroc	Farm bush	stationary	Herd	16.07.2017	Mowoto	F	6
SL 2017-881	Pool 95	neg	1,01	pos	South	Moyamba	Mowoto	Swine	West Africa Duroc	Farm bush	stationary	Herd	16.07.2017	Mowoto	M	6
SL 2017-882			0,49	neg	South	Moyamba	Mowoto	Swine	West Africa Duroc	Farm bush	stationary	Herd	16.07.2017	Mowoto	M	6
SL 2017-883			0,55	neg	South	Moyamba	Mowoto	Swine	West Africa Duroc	Farm bush	stationary	Herd	16.07.2017	Mowoto	F	18
SL 2017-884			0,42	neg	South	Moyamba	Mowoto	Swine	West Africa Duroc	Farm bush	stationary	Herd	16.07.2017	Mowoto	F	12
SL 2017-885			0,59	neg	South	Moyamba	Mowoto	Swine	West Africa Duroc	Farm bush	stationary	Herd	16.07.2017	Mowoto	F	12
SL 2017-886	Pool 96	neg	1,14	pos	South	Moyamba	Mowoto	Swine	West Africa Duroc	Farm bush	stationary	Herd	16.07.2017	Mowoto	F	12
SL 2017-887			1,07	pos	South	Moyamba	Kawela	Swine	West Africa Duroc	Farm bush	stationary	Herd	28.07.2017	Foya	M	12
SL 2017-888			0,78	pos	South	Moyamba	Kawela	Swine	West Africa Duroc	Farm bush	stationary	Herd	28.07.2017	Foya	F	36
SL 2017-889			0,68	pos	South	Moyamba	Kawela	Swine	West Africa Duroc	Farm bush	stationary	Herd	28.07.2017	Foya	F	36
SL 2017-890			0,68	pos	South	Moyamba	Kawela	Swine	West Africa Duroc	Farm bush	stationary	Herd	28.07.2017	Foya	F	6
SL 2017-891	Pool 97	neg	0,31	neg	South	Moyamba	Kawela	Swine	West Africa Duroc	Farm bush	stationary	Herd	28.07.2017	Foya	F	6
SL 2017-892			0,35	neg	South	Moyamba	Kawela	Swine	West Africa Duroc	Farm bush	stationary	Herd	28.07.2017	Foya	F	6
SL 2017-893			0,47	neg	South	Moyamba	Kawela	Swine	West Africa Duroc	Farm bush	stationary	Herd	28.07.2017	Foya	M	7
SL 2017-894			1,56	pos	South	Moyamba	Kawela	Swine	West Africa Duroc	Farm bush	stationary	Herd	28.07.2017	Foya	F	6
SL 2017-895			0,74	pos	South	Moyamba	Momenga	Swine	West Africa Duroc	Farm bush	stationary	Herd	09.09.2017	Momenga	F	60
SL 2017-896	Pool 98	neg	1,60	pos	South	Moyamba	Momenga	Swine	West Africa Duroc	Farm bush	stationary	Herd	09.09.2017	Momenga	F	48
SL 2017-897			0,89	pos	South	Moyamba	Momenga	Swine	West Africa Duroc	Farm bush	stationary	Herd	09.09.2017	Momenga	F	48
SL 2017-898			0,97	pos	South	Moyamba	Momenga	Swine	West Africa Duroc	Farm bush	stationary	Herd	09.09.2017	Momenga	M	48
SL 2017-899			0,98	pos	South	Moyamba	Momenga	Swine	West Africa Duroc	Farm bush	stationary	Herd	09.09.2017	Momenga	F	36
SL 2017-900			3,07	pos	South	Moyamba	Momenga	Swine	West Africa Duroc	Farm bush	stationary	Herd	09.09.2017	Momenga	F	36

Supplemental Table S2 HEV ELISA Validation

Sample number	pretested finding/result	OD (p239)	relative OD (6A2)	Sample number	pretested finding/result	OD (p239)	relative OD (6A2)	Sample number	pretested finding/result	OD (p239)	relative OD (6A2)
1	neg	0,14	13,41	89	positiv	1,81	177,78	166	neg	0,11	8,85
2	neg	0,09	8,57	90	positiv	3,61	353,46	167	neg	0,12	9,64
3	neg	0,11	10,57	91	positiv	3,07	300,86	168	neg	0,09	7,18
4	neg	0,14	13,11	92	positiv	3,58	351,06	169	neg	0,09	7,00
5	neg	0,09	8,78	93	positiv	3,65	357,53	170	neg	0,08	6,64
6	neg	0,08	8,19	94	positiv	2,93	287,58	171	neg	0,10	7,77
7	neg	0,09	8,27	95	positiv	3,36	329,42	172	neg	0,10	7,88
8	neg	0,10	9,82	96	positiv	2,45	240,20	173	neg	0,09	7,50
9	neg	0,13	12,84	97	positiv	0,37	36,46	174	neg	0,10	8,41
10	neg	0,11	11,13	98	positiv	3,61	353,60	175	neg	0,09	7,19
11	borderline	0,38	36,49	99	neg	0,10	9,57	176	neg	0,08	6,73
12	neg	0,11	10,51	100	positiv	3,56	348,91	177	neg	0,10	7,85
13	neg	0,11	10,33	101	positiv	3,48	341,34	178	neg	0,09	7,65
14	neg	0,08	7,29	102	positiv	3,50	342,80	179	neg	0,09	6,95
15	neg	0,14	13,38	103	positiv	3,34	327,81	180	neg	0,09	7,07
16	neg	0,11	10,73	104	positiv	3,55	348,25	181	neg	0,09	7,65
17	neg	0,09	9,14	105	positiv	3,72	364,76	182	neg	0,20	16,07
18	neg	0,13	12,63	106	neg	0,17	16,64	183	neg	0,09	7,10
19	neg	0,10	9,87	107	neg	0,31	30,22	184	neg	0,08	6,73
20	neg	0,23	22,10	108	positiv	1,02	99,98	185	neg	0,10	7,82
21	neg	0,10	9,85	109	neg	0,12	11,47	186	neg	0,08	6,65
22	pos	1,49	144,85	110	positiv	3,30	323,94	187	neg	0,10	8,06
23	neg	0,11	10,72	111	positiv	1,38	134,97	188	neg	0,09	6,96
24	neg	0,33	32,00	112	positiv	3,01	294,83	189	neg	0,09	7,40
25	neg	0,09	9,04	113	positiv	2,73	267,78	190	neg	0,11	8,56
26	neg	0,15	14,83	114	neg	0,60	58,80	191	neg	0,08	6,59
27	pos	2,63	255,56	115	neg	0,09	8,74	192	neg	0,08	6,43
28	neg	0,10	9,99	116	neg	0,09	8,56	193	neg	0,09	6,90
29	neg	2,74	266,00	117	neg	0,08	7,75	194	neg	0,08	6,52
30	neg	0,10	9,98	118	neg	0,09	8,33	195	neg	0,08	6,50
31	pos	0,56	54,62	119	neg	0,08	7,55	196	neg	0,09	7,23
32	neg	0,08	8,09	120	neg	0,09	8,88	197	neg	0,17	13,54
33	neg	0,10	9,47	121	neg	0,10	10,25	198	neg	0,17	13,64
34	positiv	2,98	288,95	122	neg	0,08	7,93	199	neg	0,15	12,23
35	neg	0,08	7,79	123	neg	0,09	8,51	200	neg	0,12	9,52
36	neg	0,75	73,00	124	neg	0,09	8,40	201	neg	0,15	12,35
37	neg	0,08	8,13	125	neg	0,08	7,85	pos control (mAB 6A2)		1,24	
38	neg	0,09	9,06	126	neg	0,08	7,66	negative control		0,12	

39	neg	0,09	8,55
40	neg	0,19	18,32
41	neg	0,09	8,96
42	neg	0,08	8,04
43	positiv	1,85	179,25
44	positiv	2,32	224,96
45	positiv	2,88	279,55
46	positiv	0,23	22,57
47	positiv	2,39	231,76
48	fraglich	0,25	24,28
49	neg	0,09	8,35
50	neg	0,09	8,69
51	positiv	2,93	284,60
52	positiv	3,01	291,93
53	positiv	3,15	305,46
54	positiv	3,09	300,31
55	positiv	1,71	166,34
56	positiv	2,36	229,05
57	positiv	2,86	277,57
58	positiv	3,08	299,19
59	positiv	2,91	282,88
60	positiv	1,50	145,96
61	positiv	2,71	263,31
62	positiv	2,84	275,40
63	positiv	1,72	167,44
64	neg	0,26	24,88
65	neg	0,15	14,39
66	positiv	2,33	225,76
67	positiv	3,07	297,99
68	positiv	2,55	247,49
69	positiv	3,27	317,88
70	positiv	2,84	275,53
71	positiv	3,12	303,30
72	positiv	3,01	292,41
73	positiv	3,10	300,56
74	positiv	1,15	112,10
75	positiv	3,19	310,04
76	positiv	3,08	298,99
77	positiv	0,08	7,65
78	positiv	3,13	303,63
79	positiv	1,95	189,71
80	positiv	2,88	279,71
81	positiv	3,24	314,34
82	positiv	3,00	291,52

127	neg	0,08	7,88
128	neg	0,10	9,76
129	neg	0,09	8,87
130	neg	0,08	7,99
131	neg	0,08	7,98
132	neg	0,08	7,98
133	neg	0,09	8,91
134	neg	0,09	9,03
135	neg	0,09	8,83
136	neg	0,29	28,64
137	neg	0,12	11,55
138	positiv	1,72	168,59
139	neg	0,09	8,94
140	positiv	0,42	41,23
141	neg	0,08	8,19
142	positiv	2,78	272,48
143	neg	0,07	7,25
144	neg	0,09	8,68
145	neg	0,10	9,75
146	neg	0,08	8,32
147	positiv	1,18	115,77
148	neg	0,09	8,78
149	neg	0,08	7,95
150	positiv	0,53	51,92
151	positiv	0,68	66,20
152	neg	0,14	13,85
153	neg	0,09	9,15
154	positiv	0,54	52,78
155	neg	0,23	22,69
156	neg	0,09	9,31
157	neg	0,11	11,06
158	positiv	0,46	45,05
159	neg	0,51	49,97
160	neg	0,14	13,50
161	neg	0,33	32,79
162	neg	0,13	13,13
163	neg	0,10	9,41
164	neg	0,22	21,71
165	neg	0,08	7,86
pos control (mAb 6A2)		1,06	
negative control		0,09	

83	positiv	3,30	320,56
84	positiv	3,13	304,22
85	positiv	3,28	318,68
86	positiv	3,24	314,21
87	positiv	3,21	311,20
88	positiv	3,09	300,11
pos control (mab 6A2)		1,03	
negative control		0,09	

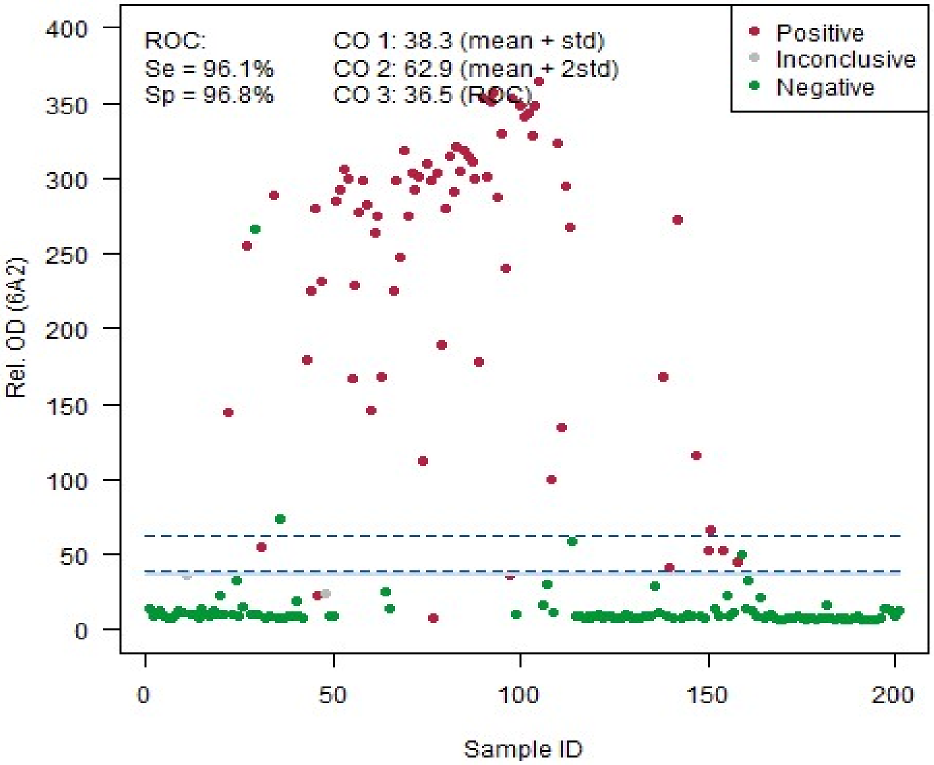

Supplement: Supplementary file 1 [file viruses-16-00558-s001.zip › viruses-2934300-supplementary.pdf]
